# A scoping review of the role of the arts in enhancing data literacy

**DOI:** 10.1371/journal.pone.0337582

**Published:** 2025-12-10

**Authors:** Helen Phelan, Fran Garry, Ailish Hannigan

**Affiliations:** 1 Irish World Academy of Music and Dance, University of Limerick, Limerick, Ireland; 2 Health Research Institute, University of Limerick, Limerick, Ireland; 3 Participatory Health Research Unit, WHO Collaborating Centre for Participatory Health Research with Refugees and Migrants, University of Limerick, Limerick, Ireland; 4 Department of Mathematics and Statistics, University of Limerick, Limerick, Ireland; Universidade Federal do Tocantins, BRAZIL

## Abstract

With the growing use of personal and public data in everyday life, data literacy is increasing in importance. There is a significant body of research indicating the role of artistic practice in the regulation of our response to information and experience, particularly as these relate to experiences of trust and social bonding. However, there is a gap in the literature concerning a review of current publications investigating the use of artistic practices to enhance data literacy. The aim of this study was to conduct a scoping review to identify and map the extent of evidence available on this topic. Specifically, the review aims to contribute to a better understanding of which artistic practices and art forms were being used, the profile of population groups and settings, and the rationale provided for using the arts. Our review utilized Arksey and O’Malley’s methodological framework. The review includes an updated search, conducted in April 2025, to provide a comprehensive overview of recent studies. Following abstract and full text screening, a total of 51 publications were deemed eligible for review. The findings indicate the use of a broad spectrum of artistic practices, with visual arts and storytelling being the most prevalent. Most of the research to date has been conducted within educational settings. The arts were identified as key tools in enhancing accessibility, engagement and critical thinking when engaging with data. Limits in the value of harnessing artistic practices for data literacy were noted, including the sometimes competing demands of artistic freedom and scientific exactitude. Further research is needed to more fully understand the importance of cultural context in how the arts are deployed in the dynamic and rapidly changing world of data.

## Introduction

Increased use of personal and public data is a growing reality in the daily lives of most people. This ‘datafication’ of everyday life is spurred by technological developments, with our dependency on technology heightened in recent times by global phenomena such as the COVID-19 pandemic. As discussed in the published protocol for this review, available here: https://doi.org/10.1371/journal.pone.0281749, this has also resulted in a growing gap between those who are data literate and those who are not, and this is linked to issues of education, socio-economic status, and discrimination [1].

There is no singular definition of data literacy. Bhargava & D’Ignazio [2] define it as “the ability to read, work with, analyze and argue with data” (p. 1). Wolff et al. [3] define data literacy as “the ability to ask and answer real world questions from large and small data sets through an inquiry process with consideration of ethical use of data” (p. 23). Definitions are linked to and influenced by the field of studies from which they emerge. Gray et al. [4] note the significant relationship between data and statistical literacy, for example, including the ability to engage critically and ethically with data sources. Being able to assess the trustworthiness and evidence-base for data has been particularly highlighted with reference to health literacy [5]. Common to these definitions is the recognition that data literacy is an important tool in empowering, informing and supporting people in their navigation of the world of information.

The marked increase in the generation and consumption of data is mirrored by the growth of misinformation and disinformation [6], particularly related to vulnerable groups. The challenges this presents are often framed as online problems with online solutions. This oversimplifies the issue as interaction with data is strongly influenced by sociocultural identities and positionalities. Such contexts influence what data we access, where it comes from, and whether or not we are likely to trust the source and content [7].

There is a growing body of research indicating the role of artistic practices in the regulation of our response to information and experience. Specific examples include using the arts as a point of entry into data literacy [8,9], using creativity to empower people in ‘non-technical fields’ to engage with data [10] or to creatively embody data [11]. This is linked to the ability of artistic practices such as drama, music, singing, dancing, drawing etc. to engage the whole-body sensorium [12], evoking physiological, affective and cognitive responses. Personal connection to data can be a strong motivator for enhanced engagement [13] and artistic practices can provide important points of emotional and experiential connection. Trust in content is often linked to trust in the source, and we are more likely to trust a source with which we feel some sense of connection or kinship [4]. The arts, particularly music and singing, have been shown to be among our most effective tools of trust-building [14]. Music plays an important role in developing interpersonal attunement [15,16], for example, and one of the evolutionary functions of singing in human societies is developing and strengthening social bonds in large and diverse groups [17]. Significantly, as a social species, bonding activities are one of the key methods humans use to build trust [15,18,19]. Thus, the potential of the arts to nurture trust and provide a strong foundation for developing data literacy has become a growing area of investigation. Bhargava [20] argues that data is often viewed as a means of finding answers and “[t]he core myth of Big Data is that if you have enough data, answers will simply emerge”. In contrast, art provokes questioning as “artistic pieces use a language of sensory experience to evoke emotional responses and reflection” (p.10). Art uses tools and techniques that help people to see things from new perspectives and to ask the right questions, while “the world of data offers a rhetorical power of legitimacy and truth” (p. 10).

Despite this, there is a gap in the literature concerning a review of current publications investigating the use of artistic processes and practices in the context of data literacy. Therefore, the key aim of this scoping review is to address this gap by providing an overview of the literature pertaining to the role of the arts in developing data literacy.

This review is underpinned by two working definitions of the arts. One draws on the 2019 review on the role of the arts in improving health and wellbeing [21] which acknowledges the challenges in providing a singular definition of the arts, while proposing a number of cross-cultural characteristics of art. These include the value of the art object in its own right, its role in the provision of imaginative experiences, and the ability to provoke an emotional response. Additionally, it is often characterized by novelty or originality, requiring specialist skills, and drawing on recognized traditions of form, composition or expression. The other, refers to the Government of Ireland Arts Act of 2003 [22] which defines the arts as “any creative or interpretative expression (whether traditional or contemporary) in whatever form, and includes visual arts, theatre, literature, music, dance, opera, film, circus and architecture” (p.3). Some creative practices such as storytelling are art forms in their own right but also span multiple other art forms [23,24]. In addition, the growing world of digital design is expanding understandings of the boundaries of art and design [25].

This review aims to identify and map the extent of current evidence so as to better understand the potential role of the arts in enhancing data literacy. As outlined in a published protocol for this study [1], this scoping review focuses on the following three key objectives:

- Identify which art forms (or combination of forms) are being reported in the published literature.
- Identify which population groups and settings are represented in the published literature.
- Analyze the findings to increase understanding of the rationale for the use of the arts to enhance data literacy.

## Methods

A scoping review was identified as the most appropriate methodology for the purpose of our study as a key purpose of a scoping review is to provide an overview of the available evidence relating to a particular topic [26–28].

As outlined in the protocol [1], our scoping review follows Arksey & O’Malley’s [26] five-stage framework and includes the sixth consultation stage recommended as necessary by Levac et al. [27]. Our scoping review adheres to the Preferred Reporting Items for Systematic Reviews and Meta-analyses extension for Scoping Reviews (PRISMA-ScR). (See S1 File for PRISMA checklist). The six-stage framework is described in the following sections.

### Stage 1: Identifying the research question

Our research question is: what is the role of the arts in enhancing data literacy? This reflects our aim to provide an overview of the literature pertaining to the role of the arts in developing data literacy.

### Stage 2: Identifying relevant studies

#### Eligibility criteria.

All publications, dated between January 2002 and April 2025, inclusive, discussing the arts in the context of data literacy were eligible for inclusion. January 2002 was chosen as the baseline as we could not find any references to data literacy prior to that. All methodologies, including qualitative, quantitative or mixed methods, and all journal articles, conference papers, reviews, books and book chapters, theses and dissertations, and grey literature were eligible for inclusion in the review. All population groups were included. Only studies in the English language were included as the researchers’ first language is English.

#### Search strategy.

Our search strategy was iteratively developed by our interdisciplinary team, with expertise in the arts (HP and FG) and data science (AH), in order to identify a body of literature appropriate to our research question. To test the effectiveness of our proposed search strategy, preliminary searches were carried out in Scopus and Web of Science Core Collection in January 2023. These test searches, incorporating minor adaptations following the recommendations of our protocol reviewers, informed our final search strategy.

For the purposes of this scoping review, we systematically searched six databases: Academic Search Complete, Dissertations & Theses A&I (ProQuest), ERIC (ProQuest), Scopus, Sociological Abstracts (ProQuest), Web of Science Core Collection, and the Google Scholar search engine, using the search string below:

(“data literacy” OR “statistical literacy”) AND (arts OR poem OR poetry OR “creative writing” OR novel* OR story* OR stories OR photography OR film OR video OR drawing OR collage OR painting OR graffiti OR textile* OR mosaic* OR masks OR artefact OR artifact OR sculpture OR singing OR song* OR music* OR danc* OR drama OR theatre OR theater OR puppetry OR “live art” OR “body art” OR “performance art” OR STEAM OR ArtScience OR SciArt).

The original search was conducted in July 2023. To capture new publications and to provide a comprehensive overview of recent studies, an updated search was conducted in late April 2025 using our original search strategy. In all searches, minor adaptations were made for varying field headings and search formats in specific databases. In Google Scholar, the first ten pages of results were screened. It was evident from scanning additional pages that there were no relevant results after the first ten pages (see S2 File for full search strategy). Google Scholar was utilized as a back-up search and predominantly yielded duplicate results. The final part of our search strategy involved searching the reference lists of included studies for any potential additional relevant publications.

### Stage 3: Study selection

Following the completion of the original database and Google Scholar searches in 2023, all citations were uploaded to Endnote 20 bibliographic software and subsequently uploaded to the Covidence systematic review software. Once duplicates were removed, authors HP & FG independently screened publications by title and abstract. Full texts of potentially relevant publications were then screened according to the inclusion criteria. Labels were created to document reasons for exclusion, and any disagreements or queries were resolved by a third reviewer (AH).

Following the completion of the updated search in April 2025, search results were exported to Endnote where duplicates were removed. To eliminate overlap (January to July 2023), results were cross-checked to remove additional duplicates. Abstracts were exported directly from database search results and authors HP and FG followed the same procedures as previously, independently screening the exported abstracts in MS Word instead of Covidence systematic review software. The authors’ experience of the initial search and screening processes informed this decision. A text-mining abstract screening application was deemed an unnecessary step at this point, as (a) it has been acknowledged that screening abstracts directly exported from the reference manager can be more efficient in smaller reviews with 300–500 results, and (b) screening involved careful reading of each abstract to make inferences from the texts [29]. The title and abstract screening stage was followed by full text screening of potentially relevant publications in line with our inclusion criteria. There were no disagreements or queries in this phase.

### Stage 4: Charting the data

Data were extracted using the MS Word data extraction form devised in our study protocol [1]. Standard recommended charting categorisations such as publication details, aims or purpose, methodological design and methods, and study population [30] were used to chart the key characteristics of the reviewed literature. In addition, categories specific to our scoping review were included as a guide for the extraction process. These additional categories were used to inform the reporting of the results, data analysis, and applying meaning to the results in line with our aims and objectives.

The data extraction form was trialled independently on the first ten studies by authors HP & FG using the MS Word data extraction form. It was agreed that the data extraction form did not require any adaptation. Following the pilot trial, authors HP & FG independently charted the remaining studies which were cross-checked to verify the data and ensure consistency of data charting (see S3 File). Once the extraction process was completed for all publications, all extracted data was transferred to MS Excel to facilitate the summarising and reporting of the results.

### Stage 5: Collating, summarising and reporting the results

The data extraction form was used to map the key study characteristics and, subsequently, to identify and group common patterns in the extracted data. This process of thematic construction [27,31] informed a narrative summary of the review findings in line with our research question, aims and objectives [30] in addition to a numerical summary of key study characteristics presented in the results section.

### Stage 6: Consultation

The protocol for this scoping review was developed within the PART-IM (Participatory and Arts-Based Methods for Involving Migrants in Health Research) research cluster, now reconstituted as the Participatory Health Research (PHR) Unit: https://www.ul.ie/ehs/medicine/research/research-groups/public-and-patient-involvement-ppi. Members of the PHR Unit working in the area of music and data literacy were consulted with a view to discussing preliminary findings and developing a dissemination strategy.

## Results

The original search, spanning 2002 to July 2023, yielded 477 results. Following the removal of duplicates, 333 abstracts were independently screened by authors HP and FG. 89 texts potentially meeting the inclusion criteria were read in full by both authors. 50 studies were excluded, at this point: 47 publications did not discuss the use of the arts in the context of data literacy, one was a book review, and two were not in English. A subsequent search alert from Web of Science yielded one additional study. The reference lists of included studies were searched for any additional studies not retrieved in the initial searches. This yielded two additional studies meeting the inclusion criteria, bringing the total number of included studies to 39.

**Fig 1:**
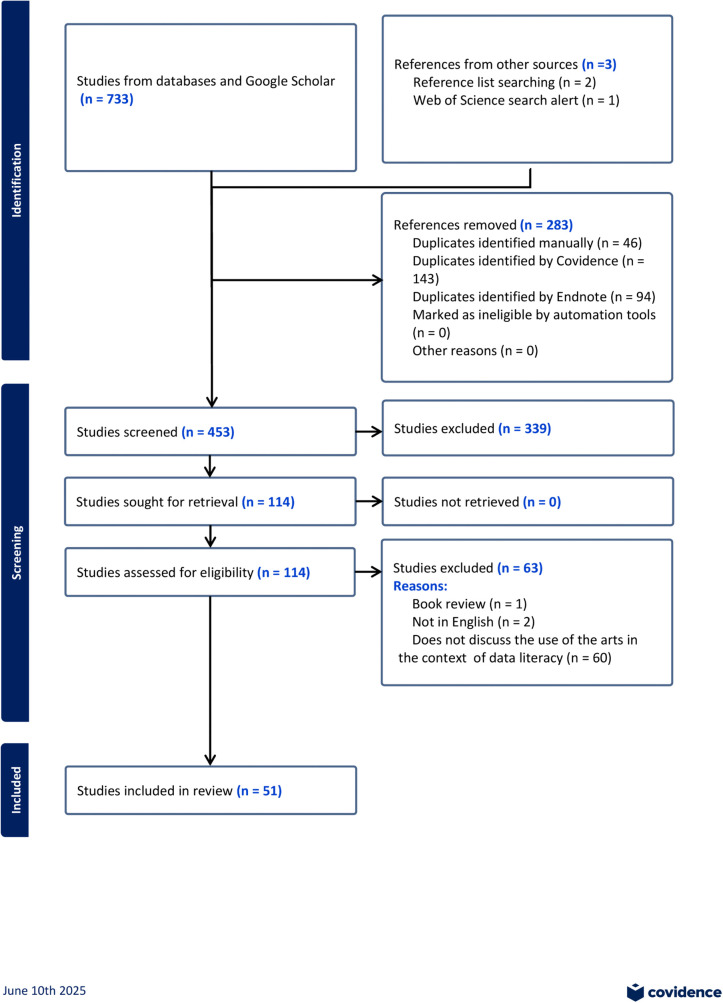
Prisma flow diagram.

The updated search, spanning January 2023 to April 2025 inclusive, yielded 256 results. Duplicates were removed in Endnote and results were manually cross-checked to identify studies that had been previously excluded or included. 42 were identified in this way and removed. Following the removal of all duplicates, 120 abstracts were independently screened by authors HP and FG, followed by full text screening of 25 publications. 13 publications were excluded at this point, as they did not discuss the use of the arts in the context of data literacy. 12 additional studies were included, bringing the total number of included studies to 51 (see Fig 1). The reference lists of additional included studies did not yield any further relevant publications. All 51 studies have been amalgamated in the following summary of results.

### Study characteristics

All 51 studies were published between 2015 and 2025, with the majority published between 2020 and 2025 (n = 41, 80%). Tables 1 and 2 provide the key study characteristics of all included studies. In the majority of studies, the first authors are based in the USA, (n = 38, 75%). In one USA study, the first author is based in the UK (n = 1, 2%). The remaining studies are from the United Kingdom (n = 2, 4%), Austria (n = 2, 4%), Belgium (n = 1, 2%), Brazil (n = 1, 2%), Denmark (n = 1, 2%), Finland (n = 2, 4%), Germany (n = 1, 2%), The Netherlands (n = 1, 2%) and Sweden (n = 1, 2%). Publication types include journal articles (n = 29, 57%), conference papers (n = 19, 37%), book chapters (n = 2, 4%) and one short paper (n = 1, 2%). We identified authors and co-authors with multiple papers, all emanating from the USA. These include Anna Amato (n = 10), Joy Bertling (n = 5), Rahul Bhargava (n = 6), Kayla DesPortes (n = 11), Catherine D’Ignazio (n = 5), Amanda Galbraith (n = 4), Shiyan Jiang (n = 4), Jennifer Kahn, J. (n = 4), Camillia Matuk (n = 10), Megan Silander (n = 9), Marian Tes (n = 9), Ralph Vacca (n = 10), and Peter J. Woods UK/ US based studies (n = 10).

**Table 1:** Study characteristics: Publication details, methods and study population.

Author(s)/Year/Country	Methodological Design and Methods	Study Population and Sample Size
Akshay & Minces, 2023 [32] USA (Study location: India)	An 18-hour long pilot data literacy workshop over three months. **Mixed Methods:** Data literacy workshop and pre- and post-workshop data literacy test to evaluate pilot workshop.	9^th^ grade students in a middle school in India. 36 students aged 14–15 years 27 students (18 girls and 9 boys) participated in a data literacy test.
Amato et al. 2022 [33] USA	“arts-based methodology” (p. 1493). Co-design of an education project. **Methods:** Qualitative. Photovoice and “photo-walking” (p. 1493) combined with ethnographic interviews, observational field notes [online], photography and artifacts produced by the 2 students interviewed; post-implementation teacher interviews. Evaluation of intervention: Analysis of two students’ experiences using a case study approach is the focus of this article.	23 eighth grade students in a private Catholic middle school. Location described as “large urban area with a predominantly Latinx and Black or African American population (85%). About 70% of students are on a free or reduced price lunch program” (p.1494).
Amato et al. 2023 [34] USA	The paper reports on the co-design of a data storytelling unit using photography and storytelling and includes qualitative analysis of photo-essays. **Methods:** Qualitative - “20 student photo-series, artist statements, data journals…semi-structured interview with 3 students […] one post-implementation interview with the art teacher” (p. 539). Evaluation of intervention: “qualitative analysis of 20 photo-essays” (p. 537) is the focus of this article.	20 8^th^ grade students “from a private middle school in a large urban area with predominantly Latine and Black or African American population (85%) […] 70% of students…are eligible for a free-or-reduced-price lunch” (p. 538).
Ambrosini and Meyer 2022 [35] Sweden	A research through design approach (RtD). “RtD is an approach where designers explore potential futures and express the knowledge gained about such possibilities through designed artifacts” (p. 11). **Methods:** Qualitative. Initial discussions, literature review and interviews informed the design of the project – a “prototype of an activity intended for use in primary schools” (p.13).	Primary school teachers. Designed for educators who work with students between the ages of 10–12 (p.12)
Arastoopour Irgens et al. 2023 [36] USA	Narrative Case Study (a single case over one year). **Methods:** Qualitative – including co-design through Research-Practice Partnerships (RPP). Data collection included: “in-person and video observations, reflective journals, artifacts, and interviews” (p. 262). The datascience unit photos were utilized “to assist in documenting the process, and students final data stories” (p.270)	5th grade students: Riverside Elementary School. Sample size not specified.
Bergner et al. 2021 [37] USA	Design research: “an iterative process often tested with a small number of participants […]“Thus, design research is necessarily collaborative. In participatory design, learners and/or teachers are consulted throughout each design cycle. In co-design, participant are further empowered to take leading roles in the design process.” (p.2018). **Methods:** Qualitative. Design research, semi-structured interviews and developing “learning environment prototypes” (p. 2019).	Teachers, coaches and students from “The Brooklyn Catholic High School (BCHS; pseudonym) …a private, all-girls, Catholic high school that serves urban, predominantly African-American and Latina students” (p.2019). Total sample size not reported.
Bertling et al. 2024 [38] USA	Design based research (DBR). **Mixed Methods:** Data collection: “student questionnaires, observations, reviews of student assessment, pre- and post-drawing exercises, and group interviews” (p. 7) Daily “exit tickets” and “retrospective post questionnaires” (p. 7). Evaluation of curriculum design intervention: researcher analysis of all data - interview data, questionnaires and field notes; semiotic analysis of imagery; and statistical representations of quantitative data.	8^th^ grade students in two suburban middle schools in southeastern USA. School population described as: “White (School A: 60%; School B: 78%, Black (School A: 23%; School B: 6%, and Hispanic/Lantinx (School A: 14%; School B: 12%, with less than 5% of students identifying as “other”” (p. 7). Students who qualify for free or reduced rate lunch, School A: 32% and School B: 11%. School A, STEM class: 16 students School B, art class 1: 16 students School B, art class 2: 20 students Total: 52 students
Bertling et al. 2025 [39] USA	Design Based Research (as per Bertling et al. 2024). **Methods:** Qualitative. The data sources for this article were confined to “predominantly to summative student assessments” (p. 57). Data collection: “students’ data visualizations and… artist statements […] field notes and student postgroup interviews” (p. 57). Group work – Art class 1: 12 visualizations; Art class 2: 17 visualizations; STEM class: 6 visualizations Researcher analysis of data visualizations, artist statements, interviews, and field notes.	8^th^ grade students in two art classes and one STEM class, as part of the larger design based study, as per Bertling et al. 2024 (above).
Bhargava et al. 2022 [8] USA	Case study. **Mixed methods**. **Qualitative:** arts-based theatre, interviews, reflection, dialogue. Use of **quantitative** data sets. The group developed movements to represent both quantitative and qualitative data in the handouts they received.	Graduate students and theatre undergraduates (p. 97). Workshop 1-Exploring Movement: 15 graduate students (in-person). Workshop 2-Prototype Data Theatre: 10 theatre undergraduates (on Zoom). Workshop 3-In-Class Data Theatre: 12 theatre undergraduates (p.97)
Bhargava and D’Ignazio 2015 [2] USA	Case study. **Mixed methods:** Literature review, development of a tool and activities. Quantitative text analysis using WordCounter.	Undergraduate students and graduates. “We used WordCounter and the accompanying lyrics analysis activity with two sets of undergraduate and graduate learners in data storytelling courses. One group included undergraduate journalism majors, while the other included students from a mix of degree programs at both undergraduate and graduate levels” (p. 4). Sample size not specified.
Bhargava et al., 2016 [40] Brazil	Case study. **Mixed methods**.Qualitative: arts-based methods (data sculpture, data murals, storytelling), informal interviews, revisiting the field for more longitudinal findings. Quantitative data sets (e.g., population numbers – school).	Students aged 16–21 from Plug Minas school in Belo Horizonte “…a state sponsored school that promotes education through innovative educational methodologies, guided by values like leadership and digital literacy […] a complement to the regular public school system” (p.202). “The story-finding and design workshops involved almost 20 student participants, the two researchers, three Plug staff, and three Escritorio staff… Approximately 50 people participated in the mural painting” (p.203).
Bhargava & D’Ignazio 2017 [9] USA	Discussion of three case studies – Case by case descriptions based on hands-on experience in multiple learning settings over 10 years. **Methods:** Qualitative – case studies, data sculptures. “First, we discuss doing short 5-minute building activities in professional development workshops with non-expert audiences. Second, we look at creations made by undergraduate and graduate students in week-long data sculpture “sketching” exercises. Third, we look at some nascent work introducing data sculptures to elementary school students in classroom settings” (pp. 2-3).	The authors refer to three specific groups: corporate staff, higher education students and elementary school students. Sample size not specified.
Blackburn 2015 [41] Germany.	Reflection on University of Queensland experiences. **Mixed methods.** Evaluation of educational intervention: Quantitative (pre and post questionnaire) and qualitative data gathering and analysis (p.466).	Participants: 385 undergraduate first-year university students in an introductory statistics course (p.464).
Bowler et al. 2020 [42] USA	The authors “report on *Exploring Data Worlds at the Public Library*, a three-year empirical research study that took place at a public library in the United States and which looked at the state of library programmes to support data literacy” (p. 2). **Methods:** qualitative - “..observation notes and feedback forms from the Exploring Data Worlds research project: (1) the observation notes from 27 data literacy workshops on 11 unique data topics, held at the public library with 95 teens, and, (2) feedback forms completed by teen participants following each workshop” (p.9).	“Approximately 95 teens [aged 14 to 17] participated in 27 after-school, drop-in workshops … [in a] mid-sized city in the North-Eastern United States, and each was designed and facilitated by members of the Exploring Data Worlds research team, with library staff in attendance” (p.9).
DesPortes et al. 2022 [11] USA	Case study. **Methods:** Qualitative – co-design of dance created from data with a maths teacher and a dance teacher. Data collection: Student group interviews, dance planning materials, and interviews with both teachers. Students chose a topic, and created and performed dances through interpretation and analysis of data sources related to the chosen topic.	A mathematics and a dance teacher and students in a public charter school, Midwestern USA. “The teachers taught the data-dance unit to eleven 7th grade students who were in both the math and dance classes (p. 306). No total sample size specified.
D’Ignazio & Bhargava 2016 [43] USA	Analysis and evaluation of three digital tools and their accompanying activities aimed at fostering data literacy. **Mixed methods:** “pre- and post-surveys, observation and analysis of generated artifacts to critically assess DataBasic” (p. 95). Workshops.	“undergraduate students and adult learners from non-profit organizations, news organizations and community advocacy groups” (p. 95). Sample size listed for the evaluation workshop only: 25 people. Age range: 20–65 with the majority aged 20–35.
D’Ignazio and Bhargava 2018 [44] USA	The paper presents a description of novel approaches to creative data literacy. **Methods:** Qualitative narrative description of learning approaches	“undergraduate and graduate students at “MIT and Emerson College,” (p. 4). No sample size specified.
Forster et al. 2018 [45] USA	This paper reports on the implementation of ‘Data Jams’ in schools but does not engage in an analysis or evaluation of these projects. Qualitative description. Vignettes were used to “illustrate how students successfully and creatively interpreted their data” (p.52) combined with direct quotes from teachers.	High School students in the USA No sample size specified. The authors note that students tend to work in groups of three when working on a data project.
Fotopoulou 2021 [46] U.K.	Empirical research drawn from a pilot training intervention. **Methods:** Qualitative: “(a)Tool-based and capacity-building work consisting of the development of the data analytics training instrument and workshops […] (b)Participant observation and a focus group interview with workshop participants. (c)Desk research consisting of mapping existing projects in the field of data literacy” (p. 1654).	Sample: 8 participants from seven organisations: “The empirical research entailed the development of a training instrument for critical data literacies, which was piloted in training workshops with seven civil society organisations in the South East of England” (p. 1642).
Galbraith et al. 2024 [47] USA (Linked to Bertling et al. studies)	Description of curriculum design, and implementation of the “Arts-Based Data-Visualization Project” (p. 8). Unit lessons included story-finding and storytelling. Qualitative description. Data gathering and analysis is not reported in this article.	Three groups of 8th grade students in two schools in a midsize city in the southern USA. STEM class: 16 students Two media arts classes: 31 and 32 students.
Giamellaro et al. 2020 [48] USA	Case Study. Qualitative – interviews, focus groups and field notes	“Thirty-three math, science, and elementary teacher participants from public schools in the Western U.S.” (p.409).
Kahn & Jiang, 2021 [13] USA	Study is described as a “micro-analysis of youth interactions with large complex, socioeconomic datasets and data visualization tools” (p. 128). Qualitative. Data collection included audio and visual recording of activities; field notes and artifacts (family data storylines) (pp. 131–132)	“Middle and high school youth” (p. 130). 17 participants. 6M and 11F (p. 130).
Kahn & Jiang 2024 [49] USA	Comparative analysis of middle and high school student cases from a design-based study merging family migration histories and large-scale data in a summer workshop. Three cases were selected for analysis. Methods: qualitative. Data gathering: analytic memos consisting of screenshots and video recordings, primarily documenting challenges in the storytelling process encountered during workshop time. Researcher analysis.	Participants are described as “…diverse middle and high school youth” in a city library in southern USA (p. 1131). Participants: 17 students
Lee et al. 2021 [50] USA	Development of a theoretical framework. Two case study examples. Qualitative description.	Case study 1: “elementary school students” (p. 667) Case study two: “middle school youths” (p. 669)
Lesser et al. 2019 [51] USA	Description of educational tool development and two pilot studies using interactive educational songs. **Mixed methods**: Evaluation of educational intervention: survey using a Likert scale and open-ended questions. “Data were also collected on students’ actions with the interface (e.g., clicking on a button or entering a response”)(p. 244).	13 college students and 77 university students.
Lesser 2025 [52] USA	The author shares and discusses “a project and activities used in workshops or a college statistical literacy course” (p. 139). Qualitative description. The paper sets out educational workshop and activity suggestions/templates for teaching statistics.	College students. Sample size not reported.
Li et al. 2023 [53] USA	“The study consisted of two parts: classroom observations and a field experiment” (p. 6). **Mixed Methods:** questionnaire and classroom observation	221 undergraduate students – across 4 universities in the USA
McDowell & Turk 2024 [54] USA	A case study of two data storytelling courses with a focus on critical data literacy (p. 322). Methods: qualitative. The researchers analyzed data gathered from 8 semesters of teaching and development on the two data storytelling courses. This included “133 project topics and 73 completed rubrics with instructor feedback” (p. 331).	College students and their instructors. Sample sizes not reported.
Markham 2020 [55] Denmark	“Experimentation, Remix Methods and an Iterative Research Design” […] The MoRM [Museum of Random Memory] was created using a “remix methods approach” (p. 230) (building meaning through varied repetition).	The general public. No sample size specified. The exhibition/ installation was open to the public in multiple countries: Denmark, Italy, Spain, Canada, and the UK (p. 227).
Matuk et al.2021 [56] USA	Co-design, implementation and evaluation of an “arts-integrated unit in a grade 7 classroom” (p.681). **Mixed methods:** Interviews (2 students), student artwork analysis, pre- and post-surveys. Researcher reflections.	Middle school students and their teachers. One art teacher [female]. One math teacher [male] and their 25 7^th^ grade students.
Matuk et al. 2022a [57] USA	Symposium: 8 posters demonstrating “the roles of storytelling in data literacy education” (p. 1780). **Mixed Methods:** Interaction between presenters and audience. Pre- and post-survey data.	Posters included project examples ranging from K-12 to university settings. > 200 statistics students in multiple high schools. Description: large proportion of “Blacks and Latinos” (p. 1783).
Matuk et al. 2022b [58] USA	Co-design and implementation of a “four data-art inquiry curriculum units: Dance, Photo-essays, Comics, and Collages” (p.1162). **Mixed methods:** classroom data, participant interviews and co-design meeting notes. Student artefacts (artworks and written responses), classroom observations, pre- and post- surveys.	10 teachers in four schools. Exact student numbers not discussed.
Matuk et al. 2024 [59] USA	Co-design and evaluation of data-art interventions. **Mixed methods:** Interview (students and teachers), teacher-generated artifacts, student generated artifacts.	Students and teachers. “The four schools served diverse student populations that varied from 2 to 65% White, 25–95% non-White students, and 40–86%
Nguyen & Parameswaran 2023 [60] USA	The study goal was “to explore how content creators engage in critical data literacies on TikTok” p. 149. The authors searched hashtags and “created a corpus of videos discussing environmental and climate action” and qualitatively coded the videos” (p. 149). **Qualitative.**	Content creators: 66% between 16 and 30 years old and 10% older than 30. Population sample size not described. 410 videos included in the sample (p. 150).
Ortega et al. 2023 [61] Netherlands.	Qualitative research design. Mixed methods: a qualitative exploration of the data comic creation process and “a quantitative between-subject study with 34 participants” (Ortega et al. 2023, sec 1) using an online survey to ascertain how the data comics were perceived by members of the public. Data comic creation sessions on Zoom platform using Miro [online whiteboard tool]: audio recording and participants’ independent comic creations over three time periods: a day, a week, a month. Qualitative analysis of recorded audio transcripts of comic creation sessions and data comics using reflexive thematic analysis combined with analysis of quantitative survey data measuring effectiveness and engagement with the data comics.	Women in their early 30s and 34 online participants [the general public] in the evaluation study, ranging in age from 21 to 59, 17 women and 17 men. Study 1 (creation of data comics) 3 women (the authors acknowledge the small sample size but specify that the women shared intimate rich personal data about their pregnancies). Study 2: Evaluation survey – 34 participants Total: 37
Otani, 2022 [62] Austria	“Interaction Design” is mentioned as a concept (p. 629). Design of a performance experience – puppet show. Described as “artistic/scientific research”. **Mixed methods:** A micro-puppet show – “While seeing the show inside a box through a peephole, the spectator activates sensors that take data to make the stars shine, blow the wind, and move the waves” (p. 629).	“[T]he puppet show reached 71 children and 158 adult spectators in 2020-2021” (p. 631). The show “Mainly addresses pre-schoolers and elementary school children” but “also seeks to involve children’s adult companions such as parents and teachers” (p. 632).
Otani 2024 [63] Austria	Artistic installation prototype development. **Mixed methods:** The interactive installation explored three research questions: “How can children recognize themselves as data subjects? How to make children’s data visible to them creatively? How can children be shown that their generated data is valuable?” (p. 970). The *Beehive* installation used sensors and motors to convert “children’s physical movements and play into digital interactions […] transforming these playful activities into online quantitative data” (p. 971).	N/A. Prototype development only. Future target group, children aged 3–5 years.
Payne et al. 2021 [64] USA	danceOn is described as part of a collaborative Design-Based Research Investigation combining dance and STEM. **Mixed methods:** Co-design – 11 formal interviews and 20 design meetings with SFD participants (Section 3.2). Design meetings, semi-structured interviews, thematic analysis. Computer programming.	Students described as “young women of color” (Section 1). 11 learners (2 dropped out) in camp 1 and 10 learners in camp 2 (Section 5.1). Total sample size unclear.
Sanei et al. 2024 [65] USA	The study is described as a [pilot] “design-based research project” (p. 162). **Mixed methods**: Data gathering methods: recordings of Zoom sessions, breakout rooms and learning activities, surveys and interviews.	High school teenagers in the U.S: Five teenagers “2 male, 3 female, ages 14-17” (p. 163).
Spence et al. 2021 [66] USA	This “user experience case study” (p. 610) describes the development of an online prototype educational tool. **Mixed methods:** “Empathy Interviews, Task Analysis, Ideation, Prototype Art, and Test Interactive Prototype” (p. 610). Evaluation: an online usability test consisting of “quantitative and qualitative questions. The system usability scale (SUS) was used to quantify the usability of the prototype” (p. 617). “A 7-point Likert scale was used to assess user interaction with the online tool” (pp. 617–618).	A founder of a data-literacy start-up, three data scientists, one art teacher, one STEAM parent, and four professional artists (p. 611).
Stornaiuolo 2020 [67] USA	Described as a “social design research study” (p. 81). **Mixed Methods**: Participant observation, artifacts, interviews, field notes, and audio recordings of classroom sessions. Pre- and post-surveys.	Adolescent students in an urban public high school with 252 students (2017−18) identifying as: “African American (75%) and LatinX (17%), as well as mixed race (3%), White (3%), Asian (1%), and Native American (1%)” (p. 87). **Sample size**: 31
Sylvan 2018 [68] USA	The article describes a series of educational initiatives designed to engage young students in data storytelling based on their own experiences. “This project combines traditional scientific research techniques and innovative new technology with its use of paper and electronics” (p. 1).	Young students and their educators, focusing on, but not limited to, the 10–14 age group.
Tylosky et al. 2021 [69] Finland	Description of proposed arts-based and data literacy workshops. **Methods:** Qualitative. Arts-based methods are described in a proposal for a hybrid workshop. The workshop also incorporates a research exchange at the start of the workshop.	Workshop open to: academics and professionals, artists, researchers and practitioners. Sample size recommended as “no more than 30 participants” (p. 338).
Vacca et al. 2022a [70] USA	Qualitative analysis of a data comics design project. **Methods:** The students “practiced data analysis skills, like graph reading and statistical reasoning, while creating digital comics to communicate stories about and with the data” (p. 2). Student interviews.	Students – with input from teachers. 33 seventh graders, two middle school teachers, and four student participants.
Vacca et al. 2022b [71] USA	“This work is part of a larger cross-curricular co-design project called Data Literacy Through the Arts (DLTA)” (p. 213). **Methods:** Qualitative. Researchers and middle school teachers co-design curriculum and resources aimed at supporting artistic data literacy for students. The DataMeme tool is described.	56 middle school students and two teachers from two schools. School 1: population described as White, Black, Hispanic and Asian students, “students with disabilities and students learning English” (p. 214). School 2: Described as a private Catholic urban middle school with a population comprising 85 per cent “Latinx and Black or African American population” (p. 214).
Van Den Bosch et al. 2022 [72] Belgium.	Design of a “video-based data storytelling application” (p. 1) aimed at interpreting weather data. “Empirical study” is mentioned in keywords but not elsewhere. Comparative study described. **Mixed methods:** app design, interviews and questionnaire (Likert scale). Controlled comparative study and “in the wild” study [observing and describing people’s actions and engaging in conversations at exhibition] (p. 4).	Exhibition: 19 adults (alone or accompanying children of particular ages). In the wild study: 30 groups and 8 individuals. Sample total not clear.
Wei 2024 [73] USA	The author reviews data visualization as a method in art education research and practice. Qualitative description. An examination of “examples of data visualization in business, marketing, social studies, and art education to understand the similarities and differences between scientific and artistic perspectives” (p. 30). Examples include literature on the topic, software programmes, art work, and art projects with the author’s students.	The author refers to art scholars and students in general.
Wolff et al. 2019 [74] U.K.	The chapter reports on the Urban Data School initiative. The UDS is described as an approach. No specific methodological design is referred to. Qualitative description.	“Lesson plans based on these data sets have been trialled in four schools—one primary school (year 5–9/10 years) and three secondary schools (2 with year 9–13/14 years,1 with year 7–11/12 years)—in Milton Keynes” (p. 162).
Wolff et al. 2021 [75] Finland	Case Study – SciberPunk. Qualitative. The authors mention a “multiple methods approach” within the arts-based section. This is explained as “combining different art genres” (p. 2)	Youth education students. No sample size given.
Woods et al. 2024 [76] (First author is U.K. – study conducted in the USA).	Co-design methodology “working alongside four US based middle school math and arts teachers with the aim of developing ‘data-art inquiry curriculum units’ or projects that simultaneously engaged students in data science and art making practices” (p. 448). Qualitative.	US middle school teachers and students. Sample size not reported.
Zhao et al. 2024 [77] USA	Design based research. **Mixed methods.** Design of a “13 week data-art inquiry programme called MVP (Mathematizing, Visualizing, and Power)” (Zhao et al. 2024, 3^rd^ page, n.p.) Evaluation of programme: Post-programme interviews. Epistemic network analysis (ENA) was used to investigate data-art connections (combines quantitative and qualitative analysis).	Student data-artists [after school programme], age range: 13–17 years. MVP Programme: 27 data artists Six participated in interviews: 4 females 2 males, described as: 1 Asian; 5 white

**Table 2:** Study characteristics: Publication details, country, art forms and settings.

Author(s) & Year	Country of Origin	Art Forms and Project Activities	Setting
Akshay & Minces, 2023 [32]	USA (Study location: India)	Visual arts, sound and music Data literacy curriculum activities: sound exploration, hands-on making, image manipulation and data visualizations	Educational setting
Amato et al. 2022 [33]	USA	Photography/storytelling Photo-walks, artist statements	Educational setting
Amato et al. 2023 [34]	USA	Photography and storytelling – photo-walks, artist statements, and the creation of photo-essays	Educational setting
Ambrosini and Meyer 2022 [35]	Sweden	* Creative/ fictional storytelling *Developing a fictitious narrative – the classroom becomes a “fictionalized spacecraft” (p. 12)	Educational setting
Arastoopour Irgens et al. 2023 [36]	USA	Singing, dancing, music listening, storytelling	Educational setting
Bergner et al. 2021 [37]	USA	Dance, body percussion, music	Educational setting
Bertling et al. 2024 [38]	USA	Visual art: digital collages; data visualizations using papier-mâché, fabric and recycled materials; data visualization installation using images and video; and examination of “professional arts-based data visualizations” (p. 12).	Educational setting
Bertling et al. 2025 [39]	USA	Visual arts. Digital collages, traditional art with found objects, papier-mâché, tempera paint. Performance art: “simulating water quality systems using found and constructed props” (p. 59).	Educational setting
Bhargava et al. 2022 [26]	USA	Participatory theatre: embodiment, gesture, mime, movement/dance, storytelling and “puppeting” (p.98)	Educational setting
Bhargava and D’Ignazio 2015 [2]	USA	Song lyrics analysis, songwriting, drawing/sketching	Educational setting
Bhargava et al., 2016 [40]	Brazil	Visual (mural, canvas painting, data sculpture) storytelling, music (background), film (of process)	Educational setting *Some community outreach – community members helped to paint the mural
Bhargava & D’Ignazio 2017 [9]	USA	Data sculptures Data sculpture sketches	Educational and Community Settings
Blackburn 2015 [41]	Germany (Reflects on experiences of University of Queensland, Australia).	Visual arts/fictional storytelling Pictures (freaky fish, seagull, pelican)	Educational setting
Bowler et al. 2020 [78]	USA	Data visualizations; arts and crafts	Educational and Community Settings
DesPortes et al. 2022 [11]	USA	Dance	Educational setting
D’Ignazio & Bhargava 2016 [43]	USA	Visual art: sketches Music: sketches created to represent song lyrics and songwriting	Educational and community settings
D’Ignazio and Bhargava 2018 [44]	USA	Visual art and data storytelling. Developing a storybook with visualizations created using crayons and markers	Educational setting
Forster et al. 2018 [45]	USA	Visual arts, music, theatre, literature, film and dance (with an emphasis on student choice)	Educational setting
Fotopoulou 2021 [46]	U.K.	Storytelling	Community setting
Galbraith et al. 2024 [47]	USA	Visual arts, story-finding and storytelling, data sculpture, media installation; creating data visualizations working with “two and three dimensional formats… using various traditional art materials such as papier-mâché, paint, modelling clay, and found objects” (Galbraith et al. 2024, p. 10). One class created a large multimedia installation to represent “mercury levels in local fish populations” (p. 11).	Educational setting
Giamellaro et al. 2020 [48]	USA	Storytelling (personal stories – real and imagined).	Educational setting
Kahn & Jiang 2021 [13]	USA	Data stories/storytelling Data visualizations represent family migration stories and oral histories	Educational and community settings
Kahn & Jiang 2024 [49]	USA	Storytelling – family migration stories	Community setting –public library summer project with educational focus.
Lee et al. 2021 [50]	USA	Storytelling and visualization	Educational setting
Lesser et al. 2019 [51]	USA	Music: Songwriting and singing	Educational setting
Lesser 2025 [52]	USA	Poetry	Educational setting
Li et al. 2023 [53]	USA	Storytelling	Educational setting
McDowell & Turk 2024 [54]	USA	Storytelling: categorized as formal, informal, and folk.	Educational setting
Markham 2020 [55]	Denmark	Visual arts, music, “architecture, filmmaking, printmaking, photography, computational art” (p. 227).	Community setting
Matuk et al.2021 [56]	USA	Data drawing and data sculpture	Educational setting
Matuk et al. 2022 [57]	USA	Storytelling	Educational setting
Matuk et al. 2022 [58]	USA	Visual arts and dance, photography, comics and collage	Educational setting
Matuk et al. 2024 [59]	USA	Dance, “photoessays”, comics and collages (p.7).	Educational setting
Nguyen & Parameswaran 2023 [60]	USA	Video images/photography Video music/audio	Online social media video focused platform - Tik Tok
Ortega et al. 2023 [61]	The Netherlands	Visual arts: digital art, data comics, storytelling	Community Setting [online]
Otani, 2022 [62]	Austria	Puppetry (as part of a theatrical show). Narrative (storytelling) and poetry. “Handmade art work/handcrafted scenography” (p. 631).	Educational and community
Otani 2024 [63]	Austria	An interactive artistic playground installation	Educational and community
Payne et al. 2021 [64]	USA	Dance	Online educational setting – Summer camp
Sanei et al. 2024 [65]	USA	Storytelling	Online educational setting – Summer project
Spence et al. 2021 [66]	USA	Visual art	Educational setting
Stornaiuolo 2020 [67]	USA	Visual arts: drawings, art and design activities, primarily T-shirts designed with the students’ data art	Educational setting
Sylvan 2018 [68]	USA	Visual arts, English language arts – narrative/storytelling	Educational setting
Tylosky et al. 2021 [69]	Finland	Proposed arts-based activities: “…roleplaying, improvisation, embodiment, sonification, physcalisation, narrativity, [storytelling] and so on […] sketches, short videos, clickable mockups or similar to convey their ideas” (p. 338).	Educational setting
Vacca et al. 2022a [70]	USA	Visual arts, Data storytelling Data comics – visual and textual elements	Educational setting
Vacca et al. 2022b [76]	USA	Visual arts, storytelling (memes)	Educational setting
Van Den Bosch et al. 2022 [72]	Belgium	Personalised video storytelling	Community and online settings
Wei 2024 [73]	USA	Visual art	Educational setting
Wolff et al. 2019 [74]	U.K.	Visual arts – hand drawings/visualizations	Educational setting
Wolff et al. 2021 [79]	Finland	Visual arts: sketches, props, photographs and videos.	Educational setting [online]
Woods et al. 2024 [76]	UK (study conducted in the USA)	Digital collage, data comics, photography, dance	Educational setting
Zhao et al. 2024 [77]	USA	Visual art – artistic data visualizations	Educational and community

Research methods for the evaluation of educational and/or community interventions are primarily qualitative (n = 29, 57%) with nine of these studies specifically referring to arts-based methodological approaches. The remaining studies utilize mixed methods approaches, primarily with a combination of ethnographic methods and questionnaires and/or surveys (n = 22, 43%).

The majority of studies comprise students (n = 39, 76%), teachers (n = 12, 24%), artists (n = 3, 6%), researchers (n = 2, 4%), the general public (n = 2, 4%), school coach (n = 1, 2%), school staff (n = 1, 2%), corporate staff (n = 1, 2%), library staff (n = 1, 2%). Where stated, ages ranged, across all studies, from 7 to 65, and participant sample numbers ranged from 3 to 221 (See Table 1).

The majority of studies utilize a combination of art forms, with visual arts (n = 35, 69%) and storytelling (n = 27, 53%) predominating, followed by music (n = 10, 20%), dance (n = 8, 16%), and theatre arts (including puppetry, role playing, improvisation, mime) (n = 4, 8%). The research settings, where stated, are primarily educational (n = 37, 73%), with three of those having taken place online. The remainder are a mix of educational and community settings (n = 7, 14%), community settings (n = 4, 8%), community combined with online (n = 2, 4%), and an online social media platform (TikTok) (n = 1, 2%) (See Table 2).

### Summary of findings

The following section summarizes the key findings in line with our research question regarding the role of the arts in enhancing data literacy. It does so with reference to our three stated objectives concerning the identification of the art forms being used; the population groups and settings in which the work is taking place; and the rationale for the use of the arts to enhance data literacy. With reference to the first objective, a summary of the art forms being used reveals an expansion of what counts as ‘the arts’ in a data context. It also notes how different definitions and understandings of data literacy impact how the arts are understood and implemented. The second section summarizes the findings regarding the populations and settings within which the work is taking place, and how the use of the arts is evaluated in these contexts. The third section presents the rationale for the use of the arts to enhance data literacy under four headings: accessibility, engagement, critical thinking and data justice.

### Identified art forms

#### Expanding understandings of what counts as the arts and data literacy.

Most of the publications within this review named artistic practices which correlate with the working definitions presented in the introduction section above [21,22]. A wide spectrum of artistic practices were referenced, with visual arts and storytelling as the most dominant across the publications. Additional creative practices included designing comics [58,61,70], creating memes [71] and writing poetry [52]. Several artistic practices were coupled with the term “data” to propose an artistic practice with a specific relationship to data, for example data stories [33] data murals [40], data sculptures [9], data theatre [8], data drawing [56], data art [38,47,67,76,77], data comics [61,70] and data memes [72]. These raise interesting questions regarding the value of the art in its own right versus its value in the context of data and whether “data art” should be considered its own category of artistic practice [56].

Related to this is the prevalent use of terms such as “data visualization” (e.g., [13,38,39,47,50,61,65,66,73,77]) and “data storytelling” (e.g., [13,36,47,49,54,72]). Data visualization draws on graphics as a means of communicating data [39]. These encompass a wide spectrum of graphics from simple charts, graphs and tables to more complex infographics and animations. These may be hand drawn as well as digital, as evidenced in this review. Many utilize characteristics common to visual arts practices such as attention to shape, line, form, space and colour. Similarly, data storytelling employs literary techniques which may include narrative, plot and character development. These may be fictionally or factually based. Distinguishing between data visualizations and data stories which may or may not be considered “art” challenges traditional understandings of what constitutes an artistic practice.

Several studies employed an expansive, multi-arts approach (e.g., [2,8,32,36,37,40,41,43–45,58–60,62,68,71,76,79]), a further reminder that the boundaries between artistic practices are often fluid and dynamic. Matuk et al. [57] also noted that “the arts” are not a generic practice and that different artistic media may support different data practices more or less effectively. Reflecting on their own work, they observed that, “narrative-based media (comics and creative writing) supported students’ introspection on their personal experiences relative to broader data trends. Movement-based art (dance) supported embodied sense making of the shape and meaning of data. Meanwhile, photography highlighted how data should be interpreted within the limits of operationalization and sampling decisions, and how images can reveal contextual details that tend to be hidden in statistics” (p.1174). Forster et al. [45] noted that artistic choice is also key: “Giving students the choice to work on what they want is important” (p. 52).

In any study on the role of the arts in data literacy, data practices themselves may invite a re-evaluation of what is understood as an artistic practice. Conversely, studies may also push the boundaries of what is understood as data literacy or data practices. Formal definitions of data literacy were provided in twenty-one publications. Bhargava and D’Ignazio’s [2] definition, as referenced in the introduction above, i.e., “the ability to read, work with, analyze and argue with data” (p. 1) was utilized by Bhargava and D’Ignazio across three additional papers [40,43,44] and in three other studies authored by Amato et al. [34], Bowler et al. [78] and Vacca et al. [70]. In another study, Bhargava et al. [8] referred to this definition but expanded it to define data literacy as, “engaging components related to acquiring data, processing and analyzing data, representing data, and storytelling with data for some purpose” (p.93). Nguyen & Parameswaran [60] referenced Bhargava & D’Ignazio’s [2] definition but also included a description from the Oceans of Data Institute which defined data literacy as including the ability to “identify, collect, evaluate, analyze, interpret, present, and protect data” (p.150). They added a further definition of *critical* data literacies which, “entail the ability to read and reason with data, critique the implications of data use, and communicate to external audiences in ways that contextualize data in personal and social settings” (p.149). Vacca et al. [71] drew on definitions from both D’Ignazio & Bhargava [43] and Wolff et al. [3]. In their 2019 paper, Wolff et al. [74] referenced their 2016 definition [3] which was also referenced by Li et al. [53]. Akshay & Minces [32] noted that “[b]eing data literate requires gaining a broad set of skills to comprehend, manipulate and utilize data to solve real world problems” (p.1). Markham [55] proposed that “[d]ata literacy is a type of awareness and curiosity that leads to developing competencies needed to grapple with the complex impacts of digital transformation on individual and cultural wellbeing” (p. 229). Stornaiuolo [67] pointed to the need for more critical perspectives in defining data literacy: “More critical perspectives on data literacy are needed to challenge approaches that position data as objective and neutral measures of the social world rather than highlighting their situated, ideological, and racialized nature” (p.82). Payne et al. [64] listed data literacy as one of the project design goals which included the provision of “an accessible interface to empower learners to understand, use, and manipulate their own data for the purposes of making art” (p. 4). Galbraith et al. [47] drew on a number of authors for their definition: “Data literacy describes how students interact with data through analysis, interpretation, evaluation (Shreiner, 2020), and examination of the source(s) and processes used to collect the data (Calabrese Barton et al., 2021; Gould, 2017)” (p. 8). McDowell & Turk [54] drew on Getz & Brodsky [80] to define data literacy as: “a subset of information literacy that teaches students to access, interpret, critically assess and “ethically use data”” (p. 324). In addition, they referred to Ridsdale et al. [81], Gummer and Mandinach [82], and Bhargava et al. [83], saying that their study “draws on all of these definitions of data literacy as a fundamental and evolving 21^st^ century information skill” (p. 324). Ortega et al. [61] drew on Gray et al. [4] and Wolff et al. [3] for their definition: “This term [data literacy] has different meanings in different contexts, but broadly it is about the knowledge and skills a person must have to successfully manage and navigate (personal) data ecosystems of which she is part” (section 1). Woods et al. [76] drew on Matuk et al. [58] and Stornaiuolo [67] to define data literacy as: “Data literacy […] includes skills related to gathering, constructing meaning from, and telling stories with data” (p. 443). Understanding data literacy as a creative process itself expands an understanding of what counts as art in this context.

### Population groups and settings

#### Population groups.

The amount of detailed information provided regarding population groups varied across studies. Thirty-four studies provided some details concerning the number of participants involved. These range from three in a single setting [61] to 221 [53] in multiple settings. Amato et al. [33], for example, focused on the analysis of two student experiences from a group of 23 participants. Li et al. [53] included data from 221 undergraduate students across four universities. Ten studies provided information on the specific age range of the participants [32,35,40,43,61,65,68,74,77,78]. In the majority of studies, age ranges were implied according to class ‘grade’ listings, primarily those in elementary, middle or high school settings. Eight studies provided details of gender [13,32,37,56,61,64,65,77] and nine studies provided information on ethnicity [33,34,37,38,57,59,67,71,77]. Akshay & Minces [32] noted that, of the 36 students between 14 and 15 years of age, 27 agreed to participate in a data literacy test. This included 18 girls and 9 boys. Amato et al. [33] described the students as follows: “Participants were 23 eighth graders from a private Catholic middle school located in a large urban area with a predominantly Latinx and Black or African American population (85%). About 70% of students are on a free or reduced price lunch program” (p.1494). In this case, the high percentage of students on free or reduced price lunches was used as a socioeconomic indicator. A similar indicator was used by DesPortes et al. [11] in introducing the setting for the study as a public charter school in the Midwestern USA, “[t]he middle school student population is 65% White, 19% Hispanic, and 6% Black—46% of the student population qualifies for free or reduced lunch” (p. 306). Bertling et al. [38] also used similar indicators in their school population descriptions: “White (School A: 60%; School B: 78%, Black (School A: 23%; School B: 6%, and Hispanic/Lantinx (School A: 14%; School B: 12%, with less than 5% of students identifying as “other”)” (p. 7). Students who qualify for free or reduced rate lunch, School A: 32% and School B: 11% (p.7).

Bowler et al. [78] provided gender data as follows: “Approximately 95 teens [aged 14 to 17] participated in 27 after-school, drop-in workshops […] Of the 95 participants, 52 identified as female and 37 as male, with six leaving the open-ended question about gender blank (‘I identify my gender as…’). The workshops were held in a teen services area in an urban, mid-sized city in the North-Eastern United States” (p. 9). Kahn & Jiang [13] described a study with 17 middle and high school participants, noting that six were male and 11 female who “self-identified as 13 African-American; 3 White; 1 Asian” (p. 130). Sanei et al. [65] described participants as “five teens (2 male, 3 female, ages 14-17, identifying as White, Asian, Latinx, and African American) with little to no coding background, from different high schools across two states in Southeastern USA” (p. 163). Zhao et al. [77] described participants as: 27 student data artists, aged 13−17, with six interview participants described as 4 females and two males: 1 Asian and 5 white. Matuk et al. [57] described the presentation of a symposium with eight poster presentations exploring storytelling in data literacy education with examples from K-12 to university settings, including “over 200 students in non-AP statistics classes from seven high schools with high proportions of Blacks and Latinos” (p. 1783). Vacca et al. [71] was one of the few studies to report on population profiles related to disabilities, noting the participation of “[f]ifty-six middle school students and two teachers across two schools …In one school, the student population was: 33% White, 32% Black, 16% Hispanic, and 16% Asian. 46% of the student population qualifies for free or reduced lunch, 18% of students have disabilities, and 4% are English Language Learners. The other school was a private Catholic middle school located in a large urban area with a predominantly Latinx and Black or African American population (85%)” (p. 214). Population ages were more likely to be provided for younger participants (elementary, middle and high school) and less likely for undergraduate, postgraduate or adult participants. An exception to this is Ortega et al. [61] who described adult participants in data comic creation as [three] women in their 30s. In addition, 34 participants, 17 women and 17 men, completed an online survey evaluating the community-based data comics project and were described as ranging in age from 21 to 59. Population ethnicity was reported primarily among studies which took place in schools in the USA.

#### Research settings.

The majority of the studies were carried out by scholars based in the USA [2,8,9,11,13,33,34,36–39,43–45,47–49,51–54,56,58–60,64–68,70,71,73,77,78]. Where these studies included an educational or community-based intervention, these also took place in the USA. In other cases, USA based scholars carried out research in different countries. Akshay & Minces [32], for example, undertook a pilot study of a data literacy workshop in India while Bhargava et al. [40] worked with an independent consultant and a specialist in public policies and governmental management from Brazil in a data mural project based at a school in Brazil. Blackburn [41] is based at a German university but the publication reflected on experiences of eLearning using visual arts and fictional storytelling in the University of Queensland, Australia. In one other study, the first author is based in the UK but the study took place in the USA in collaboration with US based scholars [76]. Other studies took place and/or originated in Sweden [35], the UK [46,74], Denmark [55], Austria [62,63], Belgium [72], The Netherlands [61] and Finland [69,79].

Most of these studies provided descriptions and/or evaluations of the use of the arts in data literacy programmes based in school settings. These included elementary/primary school settings (e.g., [35,36,50,74]), middle schools (e.g., [11,13,33,34,38,39,47,49,56]), high schools (e.g., [37,45,49,57,67,74]), as well as projects involving undergraduate and postgraduate college or university programmes (e.g., [2,8,9,43,44,51–54]). A small number of studies took place in the community. Amato et al.’s studies [33,34], for example, were based in a school but used community data from “photo walks” in the community. Bowler et al. [78] reported on after-school, drop-in workshops in a public library setting. Similarly, Kahn & Jiang [13,49] reported on summer workshops for middle and high school youth in a public library setting. Zhao et al. [77] reported on an after-school data-art inquiry programme combining educational and community settings. Markham [55] reported on a public exhibition called MoRM [Museum of Random Memory] which was exhibited in several countries including Denmark, Italy, Spain, Canada and the UK. Some of the studies included professional development programmes and workshops. For example, Fotopoulou [46] included two training workshops with the goal of building capacity within civil society organisations. Tylosky et al. [69] described arts-based and data literacy workshops which were aimed at academics, artists, researchers and other professionals. Two studies reported on online summer programmes [64,65] with Payne et al. [64] noting the role of the COVID 19 pandemic in the decision to offer this summer camp programme online. Nguyen & Parameswaran [60] investigated critical data literacies and social media, focusing on the analysis of 410 TikTok videos. In another online community-based setting, Ortega et al. [61] reported on a two-phase data-comic project. In phase one, three women created the data-comics and, in phase two, members of the public participated in the evaluation of the perceived effectiveness of and engagement with the comics via an online survey.

### The role of the arts

The majority of the studies noted the growing presence and use of data in everyday life and therefore, the importance of supporting the enhancement of data literacy. Some were explicit in identifying the arts as providing strategies, media, and processes for achieving this, while others described innovative educational and community-based interventions which included artistic practices. This section discusses four ways in which the arts can support data literacy as explicitly identified in the literature. These include increasing accessibility, enhancing engagement, developing critical thinking, and supporting data justice.

#### Increasing accessibility of data.

Several studies [46,48,57,58,61,66,70] identified the role of the arts in increasing the accessibility of data literacy programmes, thus supporting different individuals and groups to learn about and engage with data in creative ways. Fotopoulou [46], for example, noted the need for data literacy programmes to move beyond an exclusive focus on the development of technical skills and using creative approaches such as storytelling “to make data accessible and to equip them with the resources necessary for addressing the critical and ethical questions that relate to datafication” (p. 1642). Giamellaro et al. [48] argued that storytelling can situate important facts and ideas in an accessible narrative which helps to make them more relatable (p.408). Storytelling and other art forms can support personal connections to data and shape its meaning in ways that enhance “sensemaking, identity building and empathy” [58 p.1780]. Spence et al. [66] noted that current barriers to the development of important skills such as data visualization are compounded when these are limited to disciplinary areas such as mathematics or viewed as something which can only be practised by a narrowly defined cohort of “experts”. Ortega et al. [61] noted the importance of enabling the women in their study who had no prior experience of data visualizations to create data comics based on their lived experience, contributing to a creative realm that they argue is typically dominated by data experts. Bertling et al. [38] argued that an arts-based approach to data visualization exceeds the graphical approaches used in STEM fields by finding ways “to produce captivating, memorable, action-inducing stories and experiences” (p. 6). Art can support widening access: “Art is a natural medium for increasing data knowledge and practising data visualization because it is accessible. Currently the skills for creating data-driven storytelling reside with professionals, but data-knowledge skills can be more widely shared through the medium of art” [66]. Similarly, Vacca et al. [70] noted that a narrow focus on statistics or mathematics fails to recognize the importance of students’ personal experiences and misses the opportunity to invite students to understand and interpret data through meaningful reflection of their own lived experiences. In an innovative project which invited students to develop “data memes”, Vacca et al. [71] proposed that creative engagement enhances accessibility by humanising data. This can also reveal important personal or social implications which might otherwise remain hidden in the context of more passive data consumption [58].

#### Enhancing engagement.

A number of studies [8,32,40,44,46,49–51,55,56,69,70,72] proposed a direct correlation between making data more accessible and increased levels of engagement. Akshay & Minces [32] noted that a key aspect of this involves increased “engagement with real world data” (p. 1). Real world data counters the sometimes abstract way in which data is presented or analysed in more mathematically-led approaches to data literacy, inviting students to consider why the information matters in wider personal and social contexts. In discussing an innovative project which involved the creation of “data murals” in a school and community context in Brazil, Bhargava et al. [40] noted that “technology-centric interventions to building data literacy have missed a crucial opportunity to look outside of the digital world for approaches and inspirations” (p.198). This project built on “a rich tradition of using the arts and the lived experiences of participants as an invitation for civic engagement” (p.198). Not only did the project support data literacy, it engaged the wider community in the project in a way which was creative, playful and fun. D’Ignazio & Bhargava [43] noted that many approaches to data focus on the need to create materials quickly in a fast moving medium, often at the expense of pedagogical engagement. The authors described the development of a number of online tools which support participation. These included tools to analyze and create song lyrics, tell stories through the creation of sketches, and use real world media such as crayons and paper to enhance engagement with online information. D’Ignazio & Bhargava [44] argued further that these approaches are of particular importance for teaching data literacy to “non-technical, adult newcomers such as humanities scholars, journalists, educators, artists and non-profit staff” (p.2) and require an alternative pedagogical approach which they call “creative data literacy”(p.1). Lesser et al. [51] described a project which utilized interactive educational songs to teach introductory statistics, noting that, “A well-written jingle or song with rhymed lyrics can help content make a more memorable impression on students than that same content delivered in prose form” (p.238) and that the use of songs improves not only recall but motivation and engagement. Markham [55] presented a public arts-based interactive museum experience designed to support data literacy, drawing on the experiences of the designers in, “activism, art, computer science, museum curation, architecture, filmmaking, printmaking, university administration, law, photography, and computational art” (p.227) to deepen curiosity, reflexivity and engagement. Tylosky et al. [69] also explored the barriers to data engagement experienced by the general public, arguing that the arts support non-verbal communication as well as experiences of fun, creativity, and empathy: “Combining arts-based approaches with data exploration and sensemaking – especially participatory sensemaking– has good potential for data inclusivity … arts-based methods may also support empathy building through data” (p. 338). In their 2024 study, Kahn & Jiang [49] argued that tension exists between data and students’ personal experiences but they suggested that this leads to learning opportunities and can “open spaces for rich reflection, engagement and learning” (p. 1131).

#### Developing critical thinking.

A third area explored by a number of publications was the ability of the arts to develop critical thinking in the context of engaging with data. Amato et al. [33] highlighted the ethical issues of representing communities, noting the limits of both data-driven and artistic representations. The development of approaches which combine both (referred to as “arts-integrated data literacy”) attempts to support critical engagement by integrating the strengths of both approaches: “While a visual artwork, such as a photograph, can tell a story about people and places, it can also be used to overgeneralize. On the other hand, quantitative data can be challenging to collect and may over represent a community’s problems (e.g., crime rates). In this study researchers and teachers co-designed an 8th-grade, arts-integrated data literacy unit to engage students critically with community data through photography” (p.1493). In their 2023 analysis of student photo-essays, Amato et al. [34] proposed that “arts-integrated approaches to data literacy have the potential to increase engagement in data reasoning and support students in making data-based arguments” (p. 537). Arastoopour Irgens et al. [36] explored the integration of storytelling and music into an elementary classroom, proposing that early intervention, combining both arts and data literacies is key to successful integrated learning. Building on practices (such as music) in which students are invested in is critical: “using authentic datasets contextualized to what students care about is an engaging way to have them think critically about data” (p. 263). In a study based on “data theatre”, Bhargava et al. [8] argued that such methods help students harness multiple intelligences (such as kinaesthetic and somatic intelligence) to support conceptual and experiential understanding. Such activities offer “an alternate entryway to building a critical data literacy” (p.105).

Lee et al. [50] noted that artistic processes and tools may also support a broader interpretation of data than that available through the exclusive use of digital analytical tools: “using digital data analysis tools provides powerful statistics and visualizations, but can limit students’ opportunities to explore more artistic visualization methods that emphasize trajectories of experience, outliers, and storytelling” p.666). In this vein, Kahn & Jiang [49] noted the importance of investigating the gaps that middle and high school students perceived between [in this study] their family migration stories and data trends as spaces of “epistemic data agency” meaning “students’ capacities to shape knowledge-building of family history data tool use and data reasoning” (p. 1131). They argued for the need to develop critical thinking to challenge the available broader data, to recognize what is missing in relation to the students’ and their families’ lived experiences, and to fill those gaps using personally generated data to tell stories of family migration. Lesser [52] also noted the importance of recognizing this intertwined relationship of data and personal life, arguing that poetry can be used to humanize statistics “where the ideas or language of statistics explores an event in one’s personal life” (p. 146). Woods et al. [76] also argued this point, describing it as claiming “the right to look” (p. 450), meaning that “students rejected data that did not speak to their lived experience” (p. 450). They further argued that their study “reveals that arts-infused pedagogies provide one (not necessarily guaranteed) avenue for students to reclaim this right to look” (p. 456). McDowell & Turk [54] concluded that “storytelling is a powerful way to reframe data literacy that has the potential to address current challenges, in data literacy pedagogy and in teaching data literacy” (p. 339).

Matuk et al. [56] argued for the emergence of a distinct form of art called “data art” which engages learners and consumers more deeply, tapping into the affective and cognitive dimensions of data and supporting more informed, critical reflection and interpretation. Woods et al. [76] argued that creating “data-art helps students to find their voice, ask personally relevant questions about data, and see themselves within data sets and data collection processes” (p. 448). Zhao et al. [77] noted that data-art inquiry programmes effectively merge art and science and “can be a notable example of STEAM education” (17^th^ page, np). Van Den Bosch et al. [72] probed more deeply into the characteristics of visual and narrative data (in the form of video content, for example), noting the value of character, plot, narrative, sound and image in enhancing empathy, personal connection, trust, and more critical engagement with content. The authors also noted the relative lack of in-depth exploration of content design and context in data literacy pedagogy as a missed opportunity in supporting a better understanding of how artistic processes can support critical engagement and challenge the growing spread of misinformation.

#### Supporting data justice.

While most of the discussion on the role of the arts focused on its potential as a creative medium, several publications [8,11,35,47,53,54,60,74,84] also pointed to its ability to enhance specific uses of data, particularly around themes of data “justice”. Bhargava et al. [8] proposed creative data literacy as an alternate approach “that builds on processes rooted in questions of justice and equity, de-centering technology and inviting sets of learners who are not engaged through current approaches” (p.105). This builds on rich traditions, particularly in the Global South, of harnessing the arts as a form of civic engagement [40]. DesPortes et al. [11] proposed that expanding data literacy to support concepts such as citizenship and empowerment can help address the social inequities such as the growing gap between the data rich and data poor. They argued further that embodied art forms such as dance create more holistic opportunities for whole-person, experiential engagement. McDowell & Turk [54] argued that data storytelling empowers students to speak up about their concerns, and that “[d]ata storytelling teaches data literacy with agency, placing interpretive power where it belongs: in the hands of storytelling citizens and future information professionals who will use data to advocate for greater justice and inclusion” (p. 326). Li et al. [53] acknowledged data as a currency of power, arguing that transdisciplinary approaches to data, embracing the full spectrum of STEAM disciplines, are necessary to engage users from diverse backgrounds. Focusing on the issue of climate and environmental action Nguyen & Parameswaran [60] asked critical questions about the data practices of online content creators. Looking specifically at TikTok videos, the authors highlighted the need for further knowledge around the use of audio, visual and narrative techniques in the communication of information and misinformation around climate action. Through the exploration of dance [64] and music/ sound-based [32] data literacy projects, authors argued for the empowering impact of creativity, innovative and artistic expression. Wolff et al. [74] proposed that these forms of empowerment can remove barriers to participation and empower understandings and experiences of citizenship. Otani’s reported interactive artworks, a micro-puppet show [62] and a playground installation [63], in the DADA-TATA project were designed with the aim of promoting children’s rights in the digital environment while, at the same time, promoting data literacy for young audiences. The key argument is that the collection of children’s data in the digital realm is a matter of grave concern.

#### Evaluating the impact of the arts.

The majority of the published studies relate to short-term research projects. While methods of evaluation were primarily qualitative, a number of studies also used quantitative tools such as surveys and questionnaires. Eight studies used pre- and post-surveys/questionnaires in conjunction with ethnographic methods, to evaluate educational interventions aimed at enhancing data literacy [32,38,41,43,56–58,67]. In the context of developing STEAM based curricula, Bertling et al. [38] utilized pre- and –post questionnaires and drawing exercises combined with interviews, observations, and field notes to assess “students’ perceived ability to read, create, and use data visualizations and conceptions and valuing of art, STEAM, and data visualization before and after the curriculum” (p. 7). Matuk et al. [57] utilized pre- and post-surveys to capture interaction between presenters and audience members at a symposium poster exhibition but they specify that “the goal of the symposium was to spark questions about using storytelling in data literacy rather than to evaluate it” (p. 1781). Blackburn [41] used a pre- and post-questionnaire to evaluate “the pedagogical effectiveness of employing a set of pictorial icons with a story-based teaching method in improving outcomes for students” (p. 464). Lesser et al. [51] used a survey incorporating a Likert scale and open-ended questions to evaluate two pilot studies involving interactive songs as a means to develop data literacy. Van den Bosch et al. [72] also used a questionnaire incorporating a Likert scale to evaluate interactions with a video-based storytelling application. Li et al. [53] used a field experiment, classroom evaluation and a questionnaire to evaluate the OCEL.AI (Open Collaborative Experiential Learning. AI) which uses storytelling as a means to enhance critical thinking and data science education. Spence et al. [66] used a combination of interviews, task analysis, pilot development, and a user survey to evaluate an online prototype tool for enhancing data literacy. They employed a usability test to quantify the usability of a prototype for an online educational tool in what is described as “user experience case study” (p. 610). Bowler et al. [42] combined feedback forms from 27 data literacy workshops and observations to evaluate public library programmes supporting data literacy. Sanei et al. [65] combined ethnography and surveys to evaluate a “design-based research project” (p. 162) utilising storytelling. In their co-designed educational project utilising “photo-walks”, Amato et al. [33] focused on the analysis of two students’ experiences in combination with post-implementation interviews with teachers as a means of evaluation. Their 2023 publication [34] focused on the evaluation of 20 student photo-essays. A number of studies were more descriptive, than evaluative, providing an introduction to a design concept or prototype (e.g., [35,55,62,63]), while others focused on arts and data-integrated curriculum development and the provision of relevant educational supports and resources (e.g., [47,52,73]). Bhargava & D’Ignazio [9] shared reflections on the authors’ hands-on experience of using data sculptures in multiple learning settings over a period of 10 years, while other studies utilized thematic analysis extrapolated from interview data (e.g., [11,58,59]) noting that evaluations had been carried out as part of their studies but as this was not the focus of these papers, details were not further elucidated. Wolff et al. [79] had planned to include attitude assessments but were unable to do so when forced to move online. Instead, they used deductive thematic analysis based on empathy constructs. Kahn & Jiang [49] used comparative analysis of middle and high school student cases from a design-based study merging family migration histories and large-scale data in a summer workshop. Ortega et al. [61] combined reflexive thematic analysis with analysis of survey data to evaluate the effectiveness of, and engagement with, their data-comics project.

## Discussion

This scoping review set out to identify and map the available evidence regarding the role of the arts in enhancing data literacy. In doing so, it aimed to contribute to a better understanding of which artistic practices were being utilized, with whom and in what contexts the work was being developed, in addition to the rationale for the role of the arts in these contexts. The findings outlined above indicate a broad spectrum of artistic practices across primarily educational settings, motivated by a desire to make data literacy more accessible and engaging, while also enhancing critical thinking, particularly around issues related to data justice. The following section discusses the implications of evaluation processes to date on the field, possible new directions based on both positive and negative findings in these studies, and the importance of attending to cultural contexts in developing the evidence base going forward.

### Challenges in the use of artistic practices to enhance data literacy

Notwithstanding the broadly positive descriptions and evaluations provided in the studies included in this scoping review, a number of cautions are outlined concerning the limits of the arts in their ability to fully support scientific explanation. Giamellaro et al. [48] noted, for example, that “[t]eachers used data stories to carry extrinsic science explanations to their students rather than the original authentic data. However, story alone does not seem to effectively carry the intrinsic scientific explanations. While there is much potential for using narrative structure to support data literacy and phenomenological understanding, greater intentionality is needed to either incorporate the phenomena of study into the narrative structure or include another mechanism to bring the phenomena into instruction” (p.423). Integrating the arts into data literacy education can be time consuming and inadvertently increase pressure on teachers and the curriculum. In some cases, the arts can also slow down knowledge acquisition compared to more literal approaches [38,39,47]. While personal connections to data were frequently noted as positive for engagement, it was also highlighted that students need support in understanding the gaps between their personal experiences and data trends including outliers and contextual factors. Interdisciplinary teacher training is critical in this regard [49]. Matuk et al. [58] noted that the rigours of data literacy and those of artistic practices do not always align, and may inadvertently create competing demands: “there were also tensions in using arts-based approaches to convey data claims as students struggled in making artistic choices based on data vs. aesthetics” (p.1174). Artistic expression may come at the cost of data accuracy, while scientific exactitude may limit creative freedom.

These are important constraints to be aware of in any evaluation of the role of the arts in enhancing data literacy. Co-design and the use of a participatory paradigm may mitigate these challenges, and the research team are currently exploring its use in an arts-based project using music and singing in the context of diversity and data literacy.

### Further directions and existing research gaps

The importance of cultural context was raised in a number of studies as requiring further attention. Kahn & Jiang [13] noted that while the arts might enhance a sense of personal connection to data, if one felt culturally under-represented or misrepresented in this data, this could have the opposite effect in terms of engagement. Certain subjects such as migration, for example, provide ample opportunities for cultural representation but if a participant’s lived experience of these cultures is complex, this can also impact engagement. The cultural positionality of the researcher (for example, enthusiasm for the topic, artistic practice or cultural engagement) might also have an unintended impact. Lee et al. [50] proposed a tripartite framework of personal, cultural and socio-political layers to further the development of a more humanistic understanding and approach to data work. As well as the need for further research concerning cultural context in understanding the role of the arts in data literacy, mediating between constructs concerning real and virtual worlds was also identified as an important area for further research. Otani [62] noted that this is of particular importance when working with children who do not always easily distinguish between online and offline spaces.

Any further research must also recognize that “historically marginalized groups such as “Blacks and Latinos” are significantly underrepresented in data science [57 p.1783] and further research is necessary to develop a more comprehensive and culturally-informed evaluation of research to date. However, Li et al. [53] also noted that, “findings indicate that it is students’ domain knowledge and experience in data science (acquirable), not students’ gender or ethnicity (not acquirable), that would make a difference in students’ data literacy” (p.10) and that the students’ previous exposure to data is of greater significance to their level of engagement than their gender or ethnicity. Overall, the studies highlight the practical value of combining the arts and data literacy in terms of pedagogical efficacy and theoretically point to its potential in the context of redressing social and educational inequities.

### Limitations

We conducted systematic searches across multiple databases, however the searches were confined to the English language only as it is the researchers’ primary language. We acknowledge that there may be studies in other languages that are focused on using the arts to enhance data literacy that are not included here. As noted in the protocol for this study, we acknowledge that the searches were confined to academic outputs and may not have captured all relevant outputs in this interdisciplinary space with the arts.

## Conclusion

The increased centrality of data in our everyday lives highlights the importance of effective educational strategies to enhance data literacy. Concerns regarding misinformation and disinformation, particularly around vulnerable groups, reinforces the importance of developing innovative, ethical and inclusive pedagogies. This is particularly important in the context of the persistent and growing divide in education contexts between STEM subjects and the arts [38]. The growing body of research indicating the potential of the arts to support meaningful engagement with information and experience motivated this review. The published literature broadly supports the positive role of the arts in enhancing data literacy, while also indicating challenges around the sometimes competing needs of artistic and scientific communications and practices. An important next step for the field would include the development of more longitudinal studies across a broader spectrum of settings and populations. The results of this review also support further investigation focusing on the importance of cultural context in decision-making concerning how and which arts are utilized with diverse populations grappling with the realities of the complex and dynamic world of data.

## Supporting information

S1 File PRISMA checklist. (DOCX)

S2 File Full search strategy. (DOCX)

S3 File Completed data extraction forms. (PDF)
