# Supplementary material for: A scoping review of the role of the arts in enhancing data literacy
Source: PLoS One. 2025 Dec 10;20(12):e0337582. doi: 10.1371/journal.pone.0337582 (PMC12694867; doi:10.1371/journal.pone.0337582)
Supplement: S2 File — (DOCX) [file pone.0337582.s002.docx]

**Full Search Records – April 2025 and July 2023**

**April 2025 update:**

**Date range: January 2023 to April 2025** (inclusive).

**Total results:** (including Google Scholar) 256

1. **Academic Search Complete (Ebscohost):** 16 results
2. **Dissertations & Theses A&I (ProQuest):** 8 results
3. **ERIC** **(ProQuest):** 18 results
4. **Scopus:** 127
5. **Sociological Abstracts** (ProQuest): 1 result
6. **Web of Science Core Collection:** 62 results
7. **Google Scholar:** (first ten pages screened) 24 results

**___________________________________________________________________________**

**July 2023**

**Date range: January 2002 to July 2023** (inclusive).

**Total results:** (including Google Scholar): 477

**Database list:**

1. **Academic Search Complete (Ebscohost):** 38 results
2. **Dissertations & Theses A&I (ProQuest):** 24 results
3. **ERIC** (ProQuest): 27 results
4. **Scopus:** 184
5. **Sociological Abstracts** (ProQuest): 3 results
6. **Web of Science Core Collection:** 148 results
7. **Google Scholar:** (first ten pages screened) 53 results

**___________________________________________________________________________**

**Search Terms:**

("data literacy" OR "statistical literacy") AND (arts OR poem OR poetry OR "creative writing" OR novel* OR story* OR stories OR photography OR film OR video OR drawing OR collage OR painting OR graffiti OR textile* OR mosaic* OR masks OR artefact OR artifact OR sculpture OR singing OR song* OR music* OR danc* OR drama OR theatre OR theater OR puppetry OR "live art" OR "body art" OR "performance art" OR STEAM OR ArtScience OR SciArt)

**___________________________________________________________________________**

**Search Update, April 2025:**

**Academic Search Complete (Ebscohost)**

Date of search: 28/04/2025

Results: 16

AB ( "data literacy" OR "statistical literacy" ) AND AB ( arts OR poem OR poetry OR "creative writing" OR novel* OR story* OR stories OR photography OR film OR video OR drawing OR collage OR painting OR graffiti OR textile* OR mosaic* OR masks OR artefact OR artifact OR sculpture OR singing OR song* OR music* OR danc* OR drama OR theatre OR theater OR puppetry OR "live art" OR "body art" OR "performance art" OR STEAM OR ArtScience OR SciArt )

**Limiters** - Publication Date: 20230101-20251231

**Expanders** - Apply equivalent subjects

**Search modes** – Proximity

___________________________________________________________________________

**Dissertations and Theses A&I (ProQuest)**

Date of search: 28/04/2025

Results: 8

[abstract("data literacy" OR "statistical literacy") AND abstract(arts OR poem OR poetry OR "creative writing" OR novel* OR story* OR stories OR photography OR film OR video OR drawing OR collage OR painting OR graffiti OR textile* OR mosaic* OR masks OR artefact OR artifact OR sculpture OR singing OR song* OR music* OR danc* OR drama OR theatre OR theater OR puppetry OR "live art" OR "body art" OR "performance art" OR STEAM OR ArtScience OR SciArt)](https://www.proquest.com/recentsearches.recentsearchtabview.recentsearchesgridview.scrolledrecentsearchlist.checkdbssearchlink:rerunsearch/55E326B7793F403APQ/None/$N?site=pqdt&t:ac=RecentSearches)Limits applied

Limited by:

Date: From January 01 2023 to December 31 2025

Manuscript type: Doctoral dissertations, Master's theses

Language: English

**___________________________________________________________________________**

ERIC (ProQuest)

Date of search: 28/04/2025

Results: 18

[abstract("data literacy" OR "statistical literacy") AND abstract(arts OR poem OR poetry OR "creative writing" OR novel* OR story* OR stories OR photography OR film OR video OR drawing OR collage OR painting OR graffiti OR textile* OR mosaic* OR masks OR artefact OR artifact OR sculpture OR singing OR song* OR music* OR danc* OR drama OR theatre OR theater OR puppetry OR "live art" OR "body art" OR "performance art" OR STEAM OR ArtScience OR SciArt)](https://www.proquest.com/recentsearches.recentsearchtabview.recentsearchesgridview.scrolledrecentsearchlist.checkdbssearchlink:rerunsearch/7A314EAABA1A4471PQ/None/$N?site=eric&t:ac=RecentSearches) Limits applied

Limited by:

Date: From January 01 2023 to April 28 2025

Language: English

___________________________________________________________________________

**Scopus**

Date of search: 28/04/25

Results: 127

TITLE-ABS-KEY ( ( "data literacy"  OR  "statistical literacy" )  AND  ( arts  OR  poem  OR  poetry  OR  "creative writing"  OR  novel*  OR  story*  OR  stories  OR  photography  OR  film  OR  video  OR  drawing  OR  collage  OR  painting  OR  graffiti  OR  textile*  OR  mosaic*  OR  masks  OR  artefact  OR  artifact  OR  sculpture  OR  singing  OR  song*  OR  music*  OR  danc*  OR  drama  OR  theatre  OR  theater  OR  puppetry  OR  "live art"  OR  "body art"  OR  "performance art"  OR  steam  OR  artscience  OR  sciart ) )  AND  PUBYEAR  >  2022

___________________________________________________________________________

**Sociological** **Abstracts**

Date of search: 28/04/25

Results: 1

[abstract("data literacy" OR "statistical literacy") AND abstract(arts OR poem OR poetry OR "creative writing" OR novel* OR story* OR stories OR photography OR film OR video OR drawing OR collage OR painting OR graffiti OR textile* OR mosaic* OR masks OR artefact OR artifact OR sculpture OR singing OR song* OR music* OR danc* OR drama OR theatre OR theater OR puppetry OR "live art" OR "body art" OR "performance art" OR STEAM OR ArtScience OR SciArt) AND la.exact("English") AND pd(20230101-20250428)](https://www.proquest.com/myresearch/savedsearches.checkdbssearchlink:rerunsearch/2859571/SavedSearches/$N?site=pqdt&t:ac=SavedSearches)

Limited by:

Date: From January 01 2023 to April 28 2025 Language: English

___________________________________________________________________________

**Web of Science**

Date of search: 28/04/2025

Results: 62

((AB=("data literacy" OR "statistical literacy" ))) AND AB=(arts OR poem OR poetry OR "creative writing" OR novel* OR story* OR stories OR photography OR film OR video OR drawing OR collage OR painting OR graffiti OR textile* OR mosaic* OR masks OR artefact OR artifact OR sculpture OR singing OR song* OR music* OR danc* OR drama OR theatre OR theater OR puppetry OR "live art" OR "body art" OR "performance art" OR STEAM OR ArtScience OR SciArt ) Index date: 2023-07-12 to 2025-04-28 [YY-MM-DD]

**__________________________________________________________________________**

**Google Scholar:** (first ten pages screened) 24 results identified

Date of search: 28/4/2025

("data literacy" OR "statistical literacy") AND (arts OR poem OR poetry OR "creative writing" OR novel* OR story* OR stories OR photography OR film OR video OR drawing OR collage OR painting OR graffiti OR textile* OR mosaic* OR masks OR artefact OR artifact OR sculpture OR singing OR song* OR music* OR danc* OR drama OR theatre OR theater OR puppetry OR "live art" OR "body art" OR "performance art" OR STEAM OR ArtScience OR SciArt)

___________________________________________________________________________

**Original Search: July 2023**

**Academic Search Complete (Ebscohost)**

Final search date: July 12^th^, 2023

Results: 38

AB ( "data literacy" OR "statistical literacy" ) AND AB ( arts OR poem OR poetry OR "creative writing" OR novel* OR story* OR stories OR photography OR film OR video OR drawing OR collage OR painting OR graffiti OR textile* OR mosaic* OR masks OR artefact OR artifact OR sculpture OR singing OR song* OR music* OR danc* OR drama OR theatre OR theater OR puppetry OR "live art" OR "body art" OR "performance art" OR STEAM OR ArtScience OR SciArt )

**Limiters** - Publication Date: 20020101-20231231

**Expanders** - Apply equivalent subjects

**Search modes** – Proximity

___________________________________________________________________________

**Dissertations and Theses A&I (ProQuest)**

Final search date: July 13^th^_,_ 2023

Results: 24

[abstract("data literacy" OR "statistical literacy") AND abstract(arts OR poem OR poetry OR "creative writing" OR novel* OR story* OR stories OR photography OR film OR video OR drawing OR collage OR painting OR graffiti OR textile* OR mosaic* OR masks OR artefact OR artifact OR sculpture OR singing OR song* OR music* OR danc* OR drama OR theatre OR theater OR puppetry OR "live art" OR "body art" OR "performance art" OR STEAM OR ArtScience OR SciArt) AND la.exact("English") AND pd(20020101-20231231)](https://www.proquest.com/myresearch/savedsearches.checkdbssearchlink:rerunsearch/2415984/SavedSearches/$N?site=eric&t:ac=SavedSearches)

Limited by:

Date: From January 01 2002 to December 31 2023

Language:

English

___________________________________________________________________________

**ERIC (ProQuest)**

Final search date: July 13^th^, 2023

Results: 27

[abstract("data literacy" OR "statistical literacy") AND abstract(arts OR poem OR poetry OR "creative writing" OR novel* OR story* OR stories OR photography OR film OR video OR drawing OR collage OR painting OR graffiti OR textile* OR mosaic* OR masks OR artefact OR artifact OR sculpture OR singing OR song* OR music* OR danc* OR drama OR theatre OR theater OR puppetry OR "live art" OR "body art" OR "performance art" OR STEAM OR ArtScience OR SciArt) AND la.exact("English") AND pd(20020101-20230713)](https://www.proquest.com/myresearch/savedsearches.checkdbssearchlink:rerunsearch/2418821/SavedSearches/$N?site=eric&t:ac=SavedSearches)

Limited by:

Date: From January 01 2002 to July 13 2023

Language: English

___________________________________________________________________________

**Scopus:**

Final search date: July 12^th^, 2023

Results: 184

("data literacy" OR "statistical literacy") AND (arts OR poem OR poetry OR "creative writing" OR novel* OR story* OR stories OR photography OR film OR video OR drawing OR collage OR painting OR graffiti OR textile* OR mosaic* OR masks OR artefact OR artifact OR sculpture OR singing OR song* OR music* OR danc* OR drama OR theatre OR theater OR puppetry OR "live art" OR "body art" OR "performance art" OR STEAM OR ArtScience OR SciArt) AND PUBYEAR >2001

_________________________________________________________________________

**Sociological Abstracts (ProQuest)**

Final search date: July 13^th^, 2023.

Results: 3

[abstract("data literacy" OR "statistical literacy") AND abstract(arts OR poem OR poetry OR "creative writing" OR novel* OR story* OR stories OR photography OR film OR video OR drawing OR collage OR painting OR graffiti OR textile* OR mosaic* OR masks OR artefact OR artifact OR sculpture OR singing OR song* OR music* OR danc* OR drama OR theatre OR theater OR puppetry OR "live art" OR "body art" OR "performance art" OR STEAM OR ArtScience OR SciArt) AND la.exact("English") AND pd(20020101-20230707)](https://www.proquest.com/myresearch/savedsearches.checkdbssearchlink:rerunsearch/2415978/SavedSearches/$N?site=eric&t:ac=SavedSearches)Limited by:

Date: From January 01 2002 to July 13 2023

Language:

English

___________________________________________________________________________

**Web of Science Core Collection: (Clarivate) [Social Sciences Citation Index** included in this database].

Final Search Date: July 12^th^, 2023

Results: 148

"data literacy" OR "statistical literacy" (Topic) and arts OR poem OR poetry OR "creative writing" OR novel* OR story* OR stories OR photography OR film OR video OR drawing OR collage OR painting OR graffiti OR textile* OR mosaic* OR masks OR artefact OR artifact OR sculpture OR singing OR song* OR music* OR danc* OR drama OR theatre OR theater OR puppetry OR "live art" OR "body art" OR "performance art" OR STEAM OR ArtScience OR SciArt (Topic)

___________________________________________________________________________

**Google Scholar**

Final search data: July 21^st^, 2023

Results: [first ten pages screened] 53 results identified.

("data literacy" OR "statistical literacy") AND (arts OR poem OR poetry OR "creative writing" OR novel* OR story* OR stories OR photography OR film OR video OR drawing OR collage OR painting OR graffiti OR textile* OR mosaic* OR masks OR artefact OR artifact OR sculpture OR singing OR song* OR music* OR danc* OR drama OR theatre OR theater OR puppetry OR "live art" OR "body art" OR "performance art" OR STEAM OR ArtScience OR SciArt)

___________________________________________________________________________
