# Supplementary material for: A scoping review of the role of the arts in enhancing data literacy
Source: PLoS One. 2025 Dec 10;20(12):e0337582. doi: 10.1371/journal.pone.0337582 (PMC12694867; doi:10.1371/journal.pone.0337582)
Supplement: S3 File — (PDF) [file pone.0337582.s003.pdf]

Akshay & Minces 2023

Charting Elements	Associated Questions
**Publication Details**	Akshay, N. & Minces, V. (2023) ‘Introducing Data Literacy in the Classroom using Sound Exploration Tools’, *EDUNINE 2023 – 7TH IEEE World Engineering Education Conference: Reimaging Engineering – Toward the Next Generation of Engineering Education, Merging Technologies in a Connected World, Proceedings 2023*. https://doi.org/10.1109/EDUNINE57531.2023.10102886
**Study ID**	Akshay & Minces 2023
**Title**	Introducing Data Literacy in the Classroom using Sound Exploration Tools
**Author(s)**	Nagarajan Akshay and Victor Minces
**Year of Publication**	2023
**Origin/Country of origin**	USA (study conducted in India)<br><br>“a middle school in South India” (second page, no page numbers).
**Publication Type**	Conference paper
**General Overview of Study**	
**Aims/purpose**	**What were the aims or purpose of the study?**<br><br>“The authors of this paper describe the online tools and a workshop they developed to introduce students to working with data in a hands-on way, using sound and multimodal interaction” (Abstract).<br><br>The aim of the study was to test the hypothesis that, “students at middle and high school level will score higher on a data literacy test after exposure to our data literacy workshops involving multimodal data driven activities” (p. 2)<br>**What was the rationale for using the arts?**<br><br>The authors argue that data science programmes geared towards third level education can be adapted for school data literacy curriculums that can extend across multiple disciplines. They argue that very few approaches use the arts as a means of expression and engagement in this context. They address this gap.

	To address a gap in existing studies on enhancing data literacy: “very few approaches have leveraged on the arts and constructionism to teach data literacy [10] [11]. There is gap to be addressed here in inclusion of data literacy programs” (p.2)
Rationale for Using the Arts in Data Literacy Context Choices:<br><br>*Increase accessibility*<br><br>*Increase engagement*<br><br>*Develop critical thinking skills*<br><br>*Other*	Increase engagement: The work is underpinned by the idea of creating a “curriculum that promotes engagement with real world data” (p. 1)<br><br>Develop critical thinking skills: “….improve reasoning and understanding of data” (p.4)
**Methodology**	**What methodological design was utilized for the study?**<br><br>A pilot workshop and data literacy test.<br><br>An 18 hour long pilot data literacy workshop, conducted over three days in a middle school in south India.<br><br>The authors describe the development of a curriculum that includes the arts and enables the students to engage with “real-world data” through “sound exploration, hands-on making, image manipulation and data visualizations” (p. 1)
**Key findings relating to the role of the arts in data literacy**	
**Methods**	**What specific methods (e.g. qualitative, quantitative, mixed methods) were utilized?**<br><br>Mixed methods<br><br>Data literacy workshop and pre- and post-workshop data literacy test<br><br>**Which art forms were used?**<br><br>Visual arts, sound and music<br><br>While the importance of all sensory data is noted, the primary focus was on sound exploration, including music.<br><br>“The data sources were divided into categories based on the way they were perceived: auditory (heard), visual (seen), tactile (touched), and language (spoken)” (second page)

	“Various activities such as sound exploration, hands-on making, image manipulation and data visualizations were introduced as part of the data literacy curriculum” (p. 1). “The workshop included activities that involved exploring data through sound using an educational program and freely available online tools focusing on the science of sound and music [16] [17]. These activities used sound as a way to interact with data operations” (p. 2).<br><br>“online tools focusing on the science of sound and music” (p.2)<br><br>The data sources were divided into categories based on the way they were perceived: auditory (heard), visual (seen), tactile (touched), and language (spoken)” (p.2). Data visualization mentioned throughout - in terms of artistic aspects, the importance of colour was noted.
	**Was data literacy defined and, if so, how?**<br><br>No exact definition of data literacy but definitions of “data science” and “data wrangling”:<br><br>Jeannette Wing defines data science as “the study of extracting value from data” (p. 2)<br><br>The authors describe what being data literate means and mention an approach:<br><br>“Being data literate requires gaining a broad set of skills to comprehend, manipulate and utilize data to solve real world problems” (p.1)<br><br>“Data literacy can be situated in an inquiry-based approach to probe data and determine good questions that can be posed for a given dataset [6]” (p.1).<br><br>“Kandel et al. describe the term ”data wrangling” as the various complex transformations a data scientist makes to bring data into a “credible and usable” form” (p. 1)
	**Was data literacy measured and, if so, how?**<br><br>Yes, through the use of a data literacy test given before and after the pilot workshop:<br><br>“A data literacy assessment was conducted before and after the above mentioned activities which indicated participants had positive perception and increased comprehension of data operations and its real world applicability after the workshop” (p.1).

	"The data represented below is from a workshop conducted for a class of 36 students aged 14 to 15 years old, studying in 9th grade in a rural school in India. Among the participants, 27 students (18 girls and 9 boys) agreed to participate in the data literacy test. The assessment included a pre-questionnaire before the first workshop session and a post-questionnaire filled out after the last session. The assessment tool used was the Data Literacy Test [20], which included 10 questions data topics such as: the test taker's general relationship with data, understanding of data concepts and the utility of visualizations. The results of the assessment are based on the responses of the 27 students."(p. 4)
	**Was there an evaluation of the role of the arts in enhancing data literacy and, if so, how?**<br><br>While there does not appear to have been a specific test regarding an evaluation of the role of the arts, the Data Literacy test evaluated the usefulness of the pilot workshop which included artistic activities such as sound and music exploration.<br><br>The authors assess the change that has occurred after the workshop activities which included the arts.<br><br>"Interactive and constructionist activities such as the ones used in the workshop can help children form a positive association with data science" (p. 4).
**Study Population**	**What population groups are being studied in the literature?**<br><br>9th grade students in a rural school in India aged 14 to 15 years (male and female)
	**What was the sample size?**<br><br>"36 students aged 14 to 15 years old, studying in 9th grade in a rural school in India. Among the participants, 27 students (18 girls and 9 boys) agreed to participate in the data literacy test" (p.4 of the PDF).
**Research Setting**	Was it a community-based setting?<br><br>Was it an educational setting? * (9th grade) school in India<br><br>Was it a healthcare setting?
**Findings/results**	**How have the arts have been used to enhance data literacy?**<br><br>The arts (particularly sound exploration and music) were used as part of a pilot workshop designed to include

	“sensory sources such as hearing, vision, and touch” (fourth page) in data literacy training.
Was the evaluation of the role of the arts positive, negative or mixed?	**Was the evaluation of the role of the arts positive, negative or mixed?**<br><br>The evaluation of the pilot workshop infers an evaluation of its content (including the arts) which were adjudicated to be positive or neutral:<br><br>“The post-test plot suggests that the students either improved or maintained their understanding of data after the workshop with low levels of standard deviation,.” (p. 4)<br><br>The authors suggest that “the workshop had a positive impact on the students’ attitudes towards data visualizations” (p.4).<br><br>Also:<br><br>“..the workshop seems to have increased the students’ interest and confidence in working with data visualizations” (p4).<br><br>*[End of Akshay & Minces 2023]*

Amato et al. 2022

**Charting Elements**	**Associated Questions**
**Publication Details**	Amato, A., Matuk, C., DesPortes, K., Silander, M., Tes, M., Vacca, R., & Woods, P. J. (2022). Postcards and photo walks: Telling community data stories through photography. In Chinn, C., Tan, E., Chan, C., & Kali, Y. (Eds.), *Proceedings of the 16th International Conference of the Learning Sciences - ICLS 2022* (pp. 1493-1496). International Society of the Learning Sciences. https://repository.isls.org/bitstream/1/8522/1/ICLS2022_1493-1496.pdf
**Study ID**	Amato et al. 2022
**Title**	Postcards and photo walks: Telling community data stories through photography.
Author(s)	Amato, Anna, Camillia Matuk, Kayla DesPortes, Megan Silander, Marian Tes, Ralph Vacca, Peter J. Woods.
Year of Publication	2022

Origin/Country of origin	USA
Publication Type	Conference paper
**General Overview of Study**	
Aims/purpose	**What were the aims or purpose of the study?**<br><br>The purpose of the study was to design, implement and reflect on, “a two-week-long grade 8 unit to explore how photo walks—an arts-based methodology that involves capturing and reflecting on photos along one’s walking route—can engage students critically with community data” (p.1493).<br><br>“We aimed to engage students critically with data through the following activities: reflecting on multiple ways to measure the health of a neighborhood, investigating community assets and issues through personal and public data, and **drawing on the arts to connect personal experiences to community statistics**” (p. 1493)<br><br>**What was the rationale for using the arts?**<br><br>The article notes the ethical issues in representing communities which effect both data science and the arts. Researchers decided to co-design a project bringing together arts-based and data approaches as a way to enhance critical data literacy:<br><br>“How communities are represented is a social and ethical challenge. It is also one that cuts across both data science and the arts (D'Ignazio & Klein, 2020).While a visual artwork, such as a photograph, can tell a story about people and places, it can also be used to overgeneralize. On the other hand, quantitative data can be challenging to collect and may over represent a community’s problems (e.g., crime rates). In this study researchers and teachers co-designed an 8th-grade, arts-integrated data literacy unit to engage students critically with community data through photography.” (p.1493)<br><br>*Develop critical thinking skills<br><br>*Increase engagement

Rationale for Using the Arts in Data Literacy Context Choices:<br><br>*Increase accessibility*<br><br>*Increase engagement*<br><br>*Develop critical thinking skills*<br><br>*Other*	
**Methodology**	**What methodological design was utilized for the study?**<br><br>Co-design of education project, interviews and field notes.<br><br>Arts-based methodological design using photo-walks (and photovoice)
**Key findings relating to the role of the arts in data literacy**	
**Methods**	**What specific methods (e.g. qualitative, quantitative, mixed methods) were utilized?**<br><br>Qualitative. Data gathering: Photographs, interviews (teachers and students), field notes, artifacts<br><br>“artifacts produced by the 2 students interviewed (letters, photography, maps, graphs, artist statements, and contributions to class discussions written in the Zoom chat);” (p. 1494) and observational field notes conducted over the five sessions on Zoom (during the pandemic) (p. 1494).<br>Analysis: a case study approach<br><br>They use photovoice and photo-walking (p. 1493)<br><br>“Photovoice, for example, engages those who are often excluded from producing and analyzing data in creating bottom-up change through storytelling (Wang & Burris, 1997). Photowalking, a collaborative activity that engages people in multisensory observations of their environments, further highlights how prior knowledge and experience affects data collection (Mainsah & Boe, 2019)” (p. 1493).<br>*Through photo-walking, the students created postcards that introduced students across the country to their community.*<br><br>“Students’ artworks were collected into a class website by the research team to enable reflection across data and a way to share their work with friends and family” (p. 1494).<br><br>Two students participated in interviews and presented their artifacts to tell stories about their community,

	positive and negative e.g. *providing an explanation about where the data was collected, explaining that a certain area is dangerous at night, photos at certain times of the day may not accurately reflect the narrative.* 1494).<br><br>**Which art forms were used?**<br><br>Photography/storytelling<br><br>* Photo-walks<br><br>* artistic statements
	**Was data literacy defined and, if so, how?**<br><br>No, but drew on, "Philip and colleagues' (2013) framework for learning about big data for democratic participation, which outlines five goals: participating in the language and tools of data science, identifying as people who can use data for purposes that interest them, engaging in a process of inquiry to understand and address social issues, recognizing how data highlights and obscures perspectives, and making sense of and communicating data using the tools of multiple disciplines." (p.1493)<br><br>"We draw on a critical data literacy perspective, which views data as situated in social contexts and challenges the notion of data as neutral" (abstract p. 1493).
	**Was data literacy measured and, if so, how?**<br><br>Not discussed<br><br>There was no exact measurement of data literacy but two student interviews were conducted and presented in tandem with artistic statements and examples of their artifacts in order to share their experience of the project.
	**Was there an evaluation of the role of the arts in enhancing data literacy and, if so, how?**<br><br>The paper is deemed to be a case study that outlines the approach and discusses the experiences of engaging in the project rather than evaluating it.<br><br>As above, artistic outputs such as photography was presented as part of the reflexive exploration of student experience.

**Study Population**	**What population groups are being studied in the literature?**<br><br>Students – private Catholic middle school – 8th graders from a predominantly Latinx and Black or African American population (as below). No age profile.<br><br>"Participants were 23 eighth graders from a private Catholic middle school located in a large urban area with a predominantly Latinx and Black or African American population (85%). About 70% of students are on a free or reduced price lunch program." (p.1494)
	**What was the sample size?**<br><br>23 student participants in the project.<br><br>Two students participated in interviews and presented their artifacts (as in 'methods')
**Research Setting**	**Was it a community-based setting?**<br><br>Was it an educational setting? Yes<br><br>Was it a healthcare setting?
**Findings/results**	**How have the arts have been used to enhance data literacy?**<br><br>Photo-walking was used as a key tool in the project, as part of a strategy to support critical engagement with community data and enhance critical data literacy .<br><br>"In their photo walks, both students demonstrated attention to informal inference-making (Makar & Rubin, 2018), visually summarizing data as a pattern or comparison and discussing context. These findings highlight future opportunities to reinforce statistical concepts, such as sampling and variation, by iterating on data analysis and photo walks" (p. 1496).
Was the evaluation of the role of the arts positive, negative or mixed?	The study concludes that, "Planning and participating in photo walks prompted students to engage with social issues" (p.1496). As this was the goal of the project, it is a positive outcome.<br><br>The authors end on a positive note, suggesting that planning and engaging in the photo-walks enabled the students to "engage with social issues" in their communities (p.1496).<br><br>"In their photo walks, both students demonstrated attention to informal inference-making (Makar & Rubin, 2018), visually summarizing data as a pattern or comparison and discussing context. These findings

	highlight future opportunities to reinforce statistical concepts, such as sampling and variation, by iterating on data analysis and photo walks" (p. 1496).<br>*[End of Amato et al. 2022]*

Amato et al. 2023

**Charting Elements**	**Associated Questions**
**Publication Details**	Amato, A., Matuk, C., Beale, J., DesPortes, K., Tes, M., Vacca, R., Woods, P.J. and Silander, M., 2023. Critical Data Storytelling through Photography. In *Proceedings of the 17th International Conference of the Learning Sciences-ICLS 2023, pp. 537-544*. International Society of the Learning Sciences.
**Study ID**	Amato et al. 2023
**Title:**	Critical Data Storytelling through Photography
Author(s)	**Who are the authors of the publication?**<br>Amato, A., Matuk, C., Beale, J., DesPortes, K., Tes, M., Vacca, R., Woods, P.J. and Silander, M.,
Year of Publication	**When was the paper/study published?**<br>2023
Origin/Country of origin	**Where was the study carried out?**<br>USA
Publication Type	**Is the publication a journal article, book or book chapter, review, opinion paper, grey literature, other?**<br>Conference paper
**General Overview of Study**	
Aims/purpose	**What were the aims or purpose of the study?**<br>To investigate "how middle school students connected data reasoning with artistic strategies

	through the construction of photo-essays” (Amato et al. 2023, p. 537).<br><br>To “discuss findings from Year 2 of co-designing and arts-integrated data literacy unit investigating the question “What contributes to a healthy neighborhood?”” (Amato et al. 2023, p. 538).<br><br>**What was the rationale for using the arts?**<br><br>The authors propose that “using arts-integrated approaches to data literacy have the potential to increase engagement in data reasoning and support students in making data-based arguments” (Amato et al. 2023, p. 537).<br><br>The authors note that more research is needed to investigate the integration of data reasoning and artistic practices in the context of developing data literacy.
Increase accessibility<br><br>*Increase engagement*<br><br>*Develop critical thinking skills*<br><br>*Other*	Increase engagement<br><br>Develop critical thinking skills
Methodology	**What methodological design was utilized for the study?**<br><br>Qualitative. The paper reports on the co-design of data storytelling unit using photography and storytelling and includes qualitative analysis of photo-essays.
**Key findings relating to the role of the arts in data literacy**	
**Methods**	**What specific methods (e.g. qualitative, quantitative, mixed methods) were utilized?**<br><br>The paper reports on the co-design of a data storytelling unit using photography and storytelling and includes qualitative analysis of photo-essays.<br><br>Qualitative. The “13-day arts-integrated data literacy unit” ran over a period of 5 weeks (Amato et al. 2023, p. 539). The unit incorporated 8 art/PE

	classes, dedicated class time in 3 maths classes and 2 ELA [English language arts] classes.<br><br>Data gathering:<br><br>“ 20 student photo-series, artists statements, data journals, 1… semi-structured interview with 3 students […] one post-implementation interview with the art teacher…” (Amato et al. 2023, p. 539).<br><br>Analysis: the 20 photo-essays are the focus of this article<br><br>**Which art forms were used?**<br><br>Photography and storytelling – photo-walks, artist statements, and the creation of photo-essays
	**Was data literacy defined and, if so, how?**<br><br>The authors use ‘D’Ignazio & Bhargava’s, 2018 definition: “Data storytelling is an interdisciplinary practice that supports *data literacy*, or ‘the ability to read, work with, analyze, and argue with data as part of a larger inquiry process’ (p. 84; D’Ignazio and Bhargava, 2018)” (Amato et al. 2023, p. 537).
	**Was data literacy measured and, if so, how?**<br><br>Data literacy was not measured.
	**Was there an evaluation of the role of the arts in enhancing data literacy and, if so, how?**<br><br>Evaluation (for this article) was conducted through “qualitative analysis of 20 photo-essays” (Amato et al. 2023, p. 537).<br><br>Researchers analyzed the photo-essays to ascertain how students used artistic strategies to communicate their messages (p. 540). The researchers used a “constant comparison approach” to identify a number of characteristics: “principles of co-design e.g. variety, contrast, pattern; elements of art e.g. line, color [sic], value; and composition strategies e.g. leading lines, background/foreground” (Amato et al. 2023, p. 540). Finally, the researchers “analyzed photo-essays for co-presence of critical data reasoning,

	data reasoning and principles of design codes" (Amato et al. 2023, p. 540).
**Study Population**	**What population groups are being studied in the literature?**<br><br>8th grade students "from a private middle school in a large urban area with predominantly Latine and Black or African American population (85%) […] 70% of students…are eligible for a free-or-reduced-price lunch" (Amato et al. 2023, p. 538).
	**What was the sample size?**<br><br>20 (8th grade students)
**Research Setting**	Was it a community-based setting?<br><br>**Was it an educational setting? Yes.**<br><br>Was it a healthcare setting?
**Findings/results**	**How have the arts have been used to enhance data literacy?**<br><br>Students engaged in photography and storytelling by creating photo-essays in order to critically engage with data about the health of their neighborhoods. They identified and explored multiple themes from different perspectives.<br><br>"…photography offered students a tool to collect and organize lived experiences as data an enabled them to draw on artistic strategies to help them construct evidence-based arguments" (Amato et al. 2023, p. 543).
	**Was the evaluation of the role of the arts positive, negative or mixed?**<br><br>Mixed. Mostly positive with a caution that students need support in this context, and that artistic strategies integrated with data in this way can provide a means of support for students "in making data-based arguments and expressing critical data literacy" (Amato et al. 2023, p. 543).

	Positive: The authors argue that the students “demonstrated both data reasoning and critical data reasoning in their artist statements and attention to principles of design in their photo series” (Amato et al. 2023, p. 540). They argue that data storytelling using photography provided the students with “multiple entry points into data reasoning” (Amato et al. 2023, p. 542). Photography was not simply a visualization tool for the students, but, in addition, was drawn on “as a form of data and as a storytelling medium” (Amato et al. 2023, p. 543).<br>*End of Amato et al. 2023*

Ambrosini and Meyer 2022

**Charting Elements**	**Associated Questions**
**Publication Details**	Ambrosini, L. and Meyer, M, "Data Bricks Space Mission: Teaching Kids about Data with Physicalization," 2022 IEEE Workshop on Visualization for Social Good (VIS4Good), Oklahoma City, OK, USA, 2022, pp. 10-14, doi: 10.1109/VIS4Good57762.2022.00007. https://doi.org/10.1109/VIS4Good57762.2022.00007
**Study ID**	Ambrosini & Meyer 2022
**Title**	Data bricks space mission: Teaching kids about data with physicalization.
**Author(s)**	Lorenzo Ambrosini, and Miriah Meyer.
**Year of Publication**	2022
**Origin/Country of origin**	**Where was the study carried out?**<br>Sweden
**Publication Type**	Conference paper
**General Overview of Study**	

**Aims/purpose**	**What were the aims or purpose of the study?**<br><br>"In this research we tackle the challenge of teaching kids about data: what it is, how it is produced, and how we can visualize it to communicate with others. We set out to design an activity for teachers to empower kids to produce data, and to acquire hands on learning about the connection between data and the world around Them" (p. 10).<br><br>"The goal of the activity is to support data literacy in young students through engaging them in the production data. The activity introduces kids to the concept of data through physicalization, aiming to instill an understanding about the potential opportunities to create and use data in everyday life" (p. 12).<br><br>The aim of the study was to design and implement a project called Data Bricks Space Mission,"an activity and toolkit to support data literacy among children through physicalization." (p.13)<br><br>**What was the rationale for using the arts?**<br><br>The literature review showed "the effectiveness of data physicalization to help people collect, understand and reflect on data" (p.11). The Data Bricks Space Mission designed to test this with children incorporated creative play with lego and a "fictional space adventure story" (abstract, p.10).<br><br>"Using Lego bricks and a fictional space adventure story, teachers can use the Data Bricks Space Mission activity to empower kids to produce data, communicate their findings, and gain a better understanding of the relationship between data and the world around them" (p.10).<br><br>"The ideal situation is that the activity could be integrated as much as possible into any lessons or subjects, complementing the current curriculum with data-based perspectives. The interviews highlighted opportunities to engage kids through fun, creative, and physical activities that fall outside of standard classroom activities" (p. 12).
Rationale for Using the Arts in Data Literacy Context Choices:<br><br>*Increase accessibility*<br><br>*Increase engagement*<br><br>*Develop critical thinking skills*	Not really answered. The paper notes a desire to "build a fruitful data education experience among youths" (p.13) It also notes that the literature review shows that data literacy education, "can enable youths to think critically about how they use data"(p.11).

Other	
**Methodology**	**What methodological design was utilized for the study?**<br><br>A research through design approach (RtD) using the "double diamond model" (P.11) combined with literature and teacher interviews.<br><br>"We took a research through design (RtD) approach throughout the project, generating new knowledge through the development of a practical, feasible solution to a problem [9]. RtD is an approach where designers explore potential futures and express the knowledge gained about such possibilities through designed artifacts [13]" (p. 11).
**Key findings relating to the role of the arts in data literacy**	
**Methods**	**What specific methods (e.g. qualitative, quantitative, mixed methods) were utilized?**<br><br>Mixed methods including RtD, interview and literature review<br><br>Qualitative – Initial discussions, literature review and interviews to inform design of the project.<br><br>**Which art forms were used?**<br><br>Primarily creative storytelling<br><br>* Creative / fictional storytelling<br><br>Developing a fictitious narrative – the classroom becomes a "fictionalized spacecraft" (p. 12).
	**Was data literacy defined and, if so, how?**<br><br>No, the paper states explicitly that, "the concept of data …is abstract and ill-defined"(p.10) and "Within the activity, there is no formal definition of data. Instead, students learn about data implicitly through data collection tasks and group presentations." (p.12)<br><br>However, it is noted that:<br><br>"Being able work with data is a vital component of many professional fields, with data skills increasingly being important for success in many jobs. And awareness of the data traces we leave in our use of digital tools has broad implications for our future privacy, security, and control of personal data" (p. 10).

	**Was data literacy measured and, if so, how?**<br><br>No, but the authors note that, “the next step for this research is to get feedback on the design…we anticipate that feedback from educators would provide insights on how to improve all aspects of the toolkit” (p.13).<br><br>Not discussed. They present a prototype of an activity intended for future use in schools.
	**Was there an evaluation of the role of the arts in enhancing data literacy and, if so, how?**<br><br>Not discussed. The authors discuss the potential usefulness of the prototype:<br><br>“The activity is designed to help educators teach students how to gather and understand data that represents a specific phenomenon. It is a simplified form of a role-playing game in which the teacher acts as a facilitator and Lego bricks serve as both props for storytelling and a tangible representation of data” (p. 12).
**Study Population**	**What population groups are being studied in the literature?**<br><br>Primary school teachers<br><br>*Designed for educators who work with students between the ages of 10-12 (p.12) –*<br><br>*It is a “prototype of an activity intended for use in primary schools.” (p.13)*
	**What was the sample size?**<br><br>Still at design phase<br><br>Not specified
**Research Setting**	Was it a community-based setting?<br><br>Was it an educational setting? Yes, primary schools for ages 10-12<br><br>Was it a healthcare setting?
**Findings/results**	**How have the arts have been used to enhance data literacy?**<br><br>Creative, fictional storytelling is incorporated into the design prototype.

Was the evaluation of the role of the arts positive, negative or mixed?	**Was the evaluation of the role of the arts positive, negative or mixed?**<br><br>Still at design phases<br><br>Evaluation not applicable as this is a prototype. The authors state that the prototype activity needs to be tested:<br><br>"Finally, the activity needs to be tested and refined based on a deployment in classrooms. A testing phase will clarify how the activity can be useful to education, as well its strengths and flaws. These tests further look at the effectiveness of data physicalization for engaging kids with data and visualization" (p. 13)..<br><br>"Combining the knowledge gained from literature and interviews with educational professionals, we present the prototype of an activity that is intended for use in primary schools. The activity represents a step towards promoting more discussions about how to build a fruitful data education experience among youths" (p. 13).<br><br>*[End of Ambrosini & Meyer 2022]*

Arastoopour Irgens et al. 2023

**Charting Elements**	**Associated Questions**
**Publication Details**	Arastoopour Irgens, G., Herro, D., Fisher, A., Adisa, I., & Abimbade, O. (2023). Bop or Flop?: Integrating Music and Data Science in an Elementary Classroom. *The Journal of Experimental Education*, *92*(2), pp. 262–286. https://doi.org/10.1080/00220973.2023.2201570
**Study ID**	Arastoopour Irgens et al. 2023
**Title**	Bop or Flop?: Integrating Music and Data Science in an Elementary Classroom
Author(s)	Who are the authors of the publication?<br><br>Arastoopour Irgens, G., Herro, D., Fisher, A., Adisa, I., & Abimbade, O.
Year of Publication	**When was the paper/study published?**<br><br>2023

Origin/Country of origin	**Where was the study carried out?**<br><br>USA
Publication Type	**Is the publication a journal article, book or book chapter, review, opinion paper, grey literature, other?**<br><br>Journal article
**General Overview of Study**	
Aims/purpose	**What were the aims or purpose of the study?**<br><br>"We used a narrative case study methodology to describe the instructional practices of one music teacher who co-designed a data science curricular unit during a summer professional development program and implemented it in her 5th-grade music classroom" (p. 262).<br><br>**What was the rationale for using the arts?**<br><br>"Motivated by calls to expand data literacies to elementary classrooms and limited research on integrated data science into everyday practices, our research draws on using authentic datasets and data storytelling to extend knowledge about integrating data science in early classroom settings" (p. 264).<br><br>"This article provides an example of how data science knowledge and practices can be integrated into elementary school curricula using data storytelling" (p. 264).<br><br>The use of storytelling was seen to be particularly useful for engaging children: "Data storytelling is appealing for all learners, particularly young children ….stories" (p. 264).<br><br>"We chose Ms. Houston [music teacher] as our case because it was "intrinsically interesting" (Merriam, 2009, p. 42) and would uncover useful mechanisms behind the effective integration of data science and non-STEM subjects in elementary classrooms" (p. 263).<br><br>Noted the importance of using things students care about (e.g. music): "using authentic datasets contextualized to what students care about is an engaging way to have them think critically about data" (p. 263).
Rationale for Using the Arts in Data Literacy Context Choices:	

Increase accessibility Yes<br><br>*Increase engagement Yes*<br><br>*Develop critical thinking skills Yes*<br><br>*Other*	
Methodology	**What methodological design was utilized for the study?**<br><br>Narrative Case Study (a single case over one year)<br><br>"In this study, we developed an RPP where we collaborated with teachers to create interest-based, locally relevant data science problems for students. Our research team includes two Learning Sciences professors, a Quantitative Methods professor, a Special Education professor, and four graduate students. As part of a multi-year funded study aimed at offering data science curricula for elementary students in rural populations, we worked with nine teachers in grades three, four, and five to co-create a data science curriculum for their students" (p. 267).
**Key findings relating to the role of the arts in data literacy**	
**Methods**	**What specific methods (e.g. qualitative, quantitative, mixed methods) were utilized?**<br><br>"Data collected for this study include in-person and video observations, reflective journals, artifacts, and interviews" (p. 262 abstract).<br><br>Mixed methods including co-design through Research-Practice Partnerships (RPP) [and the list of methods above]<br><br>artifacts including "her datascience unit, photos to assist in documenting the process, and students final data stories" (p.270).<br><br>**Which art forms were used?**<br><br>Singing, dancing, music listening, storytelling
	Was data literacy defined and, if so, how?<br><br>"Data literacies include the ability to comprehend, analyze and interpret data and their visual representations (Shreiner, 2018) and are

	necessary for everyday transactions to evaluate information (Kjelvik & Schultheis, 2019)" (pp. 262-263).
	**Was data literacy measured and, if so, how?**<br><br>In the CT-STEM data science pop-up unit, change was observed and noted by the teacher who asserted that "students understand the data, their data story, and the visualizations they are using" (p. 278).
	**Was there an evaluation of the role of the arts in enhancing data literacy and, if so, how?**<br><br>"Our data sources included: (1) in-person observations during implementations, which noted choices and challenges encountered during implementations; we observed how the teacher used data practices and integrated interest-based, student-centered, and relevant activities; (2) video observations using a Swivl (https://www.swivl.com/) recording device to capture the teacher's movement and language while teaching; (3) reflective teacher journals with prompts completed once a week during the implementation process; (4) two post-interviews to discuss the teacher's experience with data science instruction and ways it informed her implementation practices, including benefits and challenges of implementing the unit; and (5) artifacts including her data science unit, photos to assist in documenting the process, and students final data stories" (p. 270)
**Study Population**	What population groups are being studied in the literature?<br><br>5th grade students: Riverside Elementary School
	**What was the sample size?**<br><br>Not specified
**Research Setting**	Was it a community-based setting?<br><br>Was it an educational setting? Yes. Elementary school<br><br>Was it a healthcare setting?
**Findings/results**	**How have the arts have been used to enhance data literacy?**<br><br>"The class integrated music content knowledge with data science practices by creating data visualizations to support claims about musical elements of popular songs"

	(p. 280). Storytelling enhanced data literacy by fostering personal connections with data (see below).
Was the evaluation of the role of the arts positive, negative or mixed?	**Was the evaluation of the role of the arts positive, negative or mixed?**<br><br>"Our primary claim in this article is that by connecting 1) everyday discourse, 2) domain content knowledge, and 3) data science practices, teachers can effectively integrate data science education into the elementary classroom and particularly for non-STEM subjects" (p. 280)<br><br>"Moreover, our findings also support that data storytelling is a way for teachers to engage and expose children early to data science and encourage personal connections with the data (Kahn, 2020; Kahn & Jiang, 2021; Wilkerson & Laina, 2018" (p. 280).<br><br>*[End of Arastoopour Irgens et al. 2023]*

Bergner et al. 2021

**Charting Elements**	**Associated Questions**
**Publication Details**	Bergner, Y., Mund, S., Chen, O. and Payne, W. (2021) 'Leveraging interest-driven embodied practices to build quantitative literacies: A case study using motion and audio capture from dance', *Educational Technology Research and Development*, Vol. 69 Issue 4, pp. 2013-2036. https://doi.org/10.1007/s11423-020-09804-2
**Study ID**	Bergner et al. 2021
**Title**	Leveraging interest-driven embodied practices to build quantitative literacies: A case study using motion and audio capture from dance
**Author(s)**	Bergner, Yoav; Shiri Mund, Ofer Chen, and Willie Payne
**Year of Publication**	2020
**Origin/Country of origin**	**Where was the study carried out?**<br><br>USA:" Brooklyn Catholic High School (BCHS; pseudonym) is a private, all-girls, Catholic high school that serves urban, predominantly African-American and Latina students."(p.2019)

Publication Type	**Is the publication a journal article, book or book chapter, review, opinion paper, grey literature, other?**<br><br>Journal article
**General Overview of Study**	
Aims/purpose	**What were the aims or purpose of the study?**<br><br>The article reports "on an exploratory effort to design an interest-based learning experience for high school (step) dancers to engage with concepts in mathematics and data science (abstract, p.2013)<br><br>**What was the rationale for using the arts?**<br><br>Researchers proposed that basing the study on a practice the students enjoyed (i.e. dance) would increase motivation, interest and relevance "We hypothesized that generating and analyzing data from their own dance movement, through motion and audio capture, would (a) enable learners to form analogies between off-line embodied experiences and new abstract concepts and (b) support motivation to learn due to perceived relevance and usefulness of data science to dance practice." (abstract, p.2013)<br><br>"In the present work, we pursue… an interest-based, design research effort. Working with a population of high school girls who are interested in dance and active in a school-based, competitive step team, we build towards a set of data, computation, and mathematical literacies" (p.2015).<br><br>* increased engagement can be inferred from increase motivation<br><br>* critical thinking can be inferred from desired connection between off-line embodied experiences and conceptual thinking.
Rationale for Using the Arts in Data Literacy Context Choices:<br><br>*Increase accessibility*<br><br>*Increase engagement*<br><br>*Develop critical thinking skills*<br><br>*Other*	
**Methodology**	**What methodological design was utilized for the study?**<br><br>Design research. This is an "an iterative process often tested with a small number of participants (p.2018) and

	participatory: “Thus, design research is necessarily collaborative. In participatory design, learners and/or teachers are consulted throughout each design cycle. In co-design, participant are further empowered to take leading roles in the design process.” (p.2018)<br><br>“We have carried out this project as design research to develop a reciprocal relationship between two kinds of student knowledge. One direction builds from embodied knowledge to develop students’ quantitative literacy. At the same time, interactions with technology and understanding of motion/audio capture and analysis are intended to feel both relevant and useful for the learners’ established interest in dance practice” (p.2018).
**Key findings relating to the role of the arts in data literacy**	
**Methods**	**What specific methods (e.g. qualitative, quantitative, mixed methods) were utilized?**<br><br>Mixed methods including interviews, design prototype, re-interviews<br><br>“We began with a series of semi-structured interviews (Rubin and Rubin 2011) to better understand how teachers, coaches, and students communicated about their dance practice. We then developed some learning environment prototypes. This process involved thinking simultaneously about the technologies used for sensing and about the data and computational literacies that could be explored with those technologies. We now describe the setting for our development process; details of the initial exploratory interviews with the steppers and their coach, which helped set the direction for the design process; the visual and acoustic quantization work and prototype audio and visual analysis tools; and the second round of design interviews, conducted with the steppers and coach to obtain feedback on the prototypes (p.2019).<br><br>Interviews were carried out “with three primary goals: The first was to learn more about step dancing and to gain insight into the students’ connection to stepping. In addition, we sought specific elements of step that lent themselves to exploration through simple data analysis tools. Finally, we wanted to know how students conceptualized and visualized quantifiable aspects of their dancing” (p.2020).<br><br>**Which art forms were used?**<br><br>Dance, body percussion, music

	The focus was on dance but the acoustic elements of dance were also identified as important:<br><br>“Music and dance emerged as a clear theme. Of particular mention was the school’s highly competitive step team.” (p.2019)<br><br>“Stepping is a unique form of performance that involves complex routines of synchronized body percussion, using stomps, claps, and body hits—with sharp and expressive gestures—accompanied by singing, chanting, or drama. This distinct blend of elements aims to demonstrate group cohesion and elevate the group status (Fine 1991). Step routines often include high levels of synchronization between dancers, syncopation, and polyrhythms—all produced using the dancers’ bodies—which make them both visually and musically impressive” (p.2019)
	**Was data literacy defined and, if so, how?**<br><br>No
	**Was data literacy measured and, if so, how?**<br><br>Not discussed.<br><br>No, researchers noted that this could be a future goal: “Given the exploratory nature of the present study, we made no attempt to systematically assess learning gains on specific data literacy concepts. However, once learning objectives become more solidified, we believe an experimental design can be brought to bear to evaluate interest-based embodied learning approaches in comparison to more traditional form of data science instruction” (p.2031)<br><br>Researchers noted that, “Broadly speaking, the steppers did convey a feeling that learning math and data science can be more engaging through relevance to an activity of interest” (p.2031).
	**Was there an evaluation of the role of the arts in enhancing data literacy and, if so, how?**<br><br>The authors explain the collaborative nature of design research, stating that it differs from evaluation efforts:<br><br>“It differs from one-off design-and-use evaluation efforts in that the design process is integrated with the inquiry into how learning occurs and can be supported. Thus,

	design research is necessarily collaborative. In participatory design, learners and/or teachers are consulted throughout each design cycle” (p.2018).<br><br>No, but student comments were positive:<br><br>Student stepper: “this can help math make sense and it can help dance make sense!” (p.2030)<br><br>Student stepper: “’You can explain dance when you put the math behind it...you can use how dance works to trigger the math in your mind’.” (p.2030)
**Study Population**	**What population groups are being studied in the literature?**<br><br>Teachers, coaches and students from “The Brooklyn Catholic High School (BCHS; pseudonym) is a private, all-girls, Catholic high school that serves urban, predominantly African-American and Latina students.” (p.2019)
	**What was the sample size?**<br><br>Total unspecified.<br><br>“..we conducted a series of initial interviews with school faculty to learn about student interests and activities” (p.2019).<br><br>Interviews: “we conducted a series of interviews with a dance teacher, the step coach, and three members of the step team. The three student interviewees were high school seniors who had been on the team for 4 years and included one of the team captains. Each interview was approximately 45 min in duration and followed a semi structured interview protocol (Rubin and Rubin 2011). (p.2020)
**Research Setting**	Was it a community-based setting?<br><br>Was it an educational setting? * Yes, High School, as above.<br><br>Was it a healthcare setting?
**Findings/results**	**How have the arts have been used to enhance data literacy?**<br><br>Dance was used as part of a design project to enhance data literacy.<br><br>The authors posit links between embodied cognition and data representations:<br><br>“We feel more confident in the provisional evidence that off-line embodied cognition can play a role in grounding

	the abstractions of data representations in sensorimotor experience and memory" (p.2031)
Was the evaluation of the role of the arts positive, negative or mixed?	**Was the evaluation of the role of the arts positive, negative or mixed?**<br><br>Positive comments from students but it is acknowledged that only two students were interviewed and the study is exploratory.<br><br>The authors acknowledge the feedback from the steppers but emphasise that the study is exploratory and that learning gains cannot be assessed at this point:<br><br>"Broadly speaking, the steppers did convey a feeling that learning math and data science can be more engaging through relevance to an activity of interest. However, we acknowledge that only two steppers were interviewed for this study, and that the results are only suggestive. Even for the two steppers, this exposure was short, and we have not had a chance to see maintained motivation over the duration (weeks, at least) that would be required to solidify knowledge gains" (p.2031).<br><br>"Given the exploratory nature of the present study, we made no attempt to systematically assess learning gains on specific data literacy concepts. However, once learning objectives become more solidified, we believe an experimental design can be brought to bear to evaluate interest-based embodied learning approaches in comparison to more traditional form of data science instruction" (p.2031).<br><br>*[End of Bergner et al. 2021]*

Bertling et al. 2024

**Charting Elements**	**Associated Questions**
**Publication Details**	Bertling, J.G., Galbraith, A., Doss, T.W., Swartzentruber, R., Massey, M. and Christen, N., 2024. Transdisciplinary inquiry that elevates the arts? Insights from a data-visualization pilot project. *International Journal of Education & the Arts*, *25*(16).
**Study ID**	Bertling et al. 2024

**Title**	Transdisciplinary inquiry that elevates the arts? Insights from a data-visualization pilot project.
Author(s)	**Who are the authors of the publication?**<br><br>Bertling, J.G., Galbraith, A., Doss, T.W., Swartzentruber, R., Massey, M. and Christen, N.
Year of Publication	**When was the paper/study published?**<br><br>2024
Origin/Country of origin	**Where was the study carried out?**<br><br>USA
Publication Type	**Is the publication a journal article, book or book chapter, review, opinion paper, grey literature, other?**<br><br>Journal article
**General Overview of Study**	
Aims/purpose	**What were the aims or purpose of the study?**<br><br>"This study explored STEAM curricula centered around data visualization, a transdisciplinary practice commonly utilized in design and STEM fields and increasingly practiced in and reinterpreted through contemporary art" (Bertling et al. 2024, p. 3).<br><br>The authors used design based research (DBR) to investigate how "data visualization STEAM curriculum might function within middle-school STEM and art/media-arts contexts and …developed broader insights into the opportunities and challenges of transdisciplinary learning in K-12 schooling" (Bertling et al. 2024, p. 7).<br><br>**What was the rationale for using the arts?**<br><br>The authors highlight a persistent and growing divide in educational contexts between STEM subjects and the arts and humanities in the USA. They argue that the arts have been undervalued in both, thus restricting "students' access to quality arts education and, correspondingly, deny them the opportunity to benefit from the unique cognitive

	and attitudinal dispositions arts education can foster" [citing Hetland et al. 2013] (Bertling et al. 2024, p. 5). Therefore, in designing STEAM curricula, they used an arts-based data visualization approach "drawing from the data-visualization practices of contemporary artists" (Bertling et al. 2024, p. 7). They argue that this arts-based approach exceeds the graphical approaches used in STEM fields as "they find ways to make the real-world connections of the data visible – to produce captivating, memorable, action-inducing stories and experiences" (Bertling et al. 2024, p. 6).
Increase accessibility<br><br>*Increase engagement*<br><br>*Develop critical thinking skills*<br><br>*Other*	Increase engagement<br><br>Develop critical thinking skills<br><br>Introduce arts into STEM classes and introduce data into art classes
Methodology	**What methodological design was utilized for the study?**<br><br>Design based research (DBR). Drawing on Anderson and Shattuck (2012), the authors outline two key characteristics of DBR: "(1) research situated in a real education context and (2) focused on the design and testing of an intervention" (Bertling et al. 2024, p. 6).
**Key findings relating to the role of the arts in data literacy**	
**Methods**	**What specific methods (e.g. qualitative, quantitative, mixed methods) were utilized?**<br><br>Mixed methods: The authors describe their DBR approach "a mixed methods, qualitative dominant design" (Bertling et al. 2024, p. 7).<br><br>Data collection methods: "student questionnaires, observations, reviews of student assessment, pre- and post-drawing exercises, and group interviews" (Bertling et al. 2024, p. 7) Daily "exit tickets" (p.7) were used to ask students to "self-rate their class experiences related to enjoyment, thinking, effort, an attention on a five-point scale" (Bertling et al. 2024, p. 7), and in daily "retrospective post

	questionnaires" students could rate agreement with pre-set statements regarding their engagement with data visualizations. Observations, both structured and unstructured, were used for all sessions, in addition to researcher field notes.<br><br>**Which art forms were used?**<br><br>Visual art: digital collages; data visualizations using papier-mâché, fabric and recycled materials; data visualization installation using images and video; and examination of "professional arts-based data visualizations" (Bertling et al. 2024, p. 12).
	**Was data literacy defined and, if so, how?**<br><br>Data literacy was not formally defined but its meaning was implied e.g. "… students, through participation in this STEAM curriculum, were able to analyze, interpret and create socially and ecologically engaged arts-based data visualizations" (Bertling et al. 2024, p. 20).
	**Was data literacy measured and, if so, how?**<br><br>As per methods: "student questionnaires, observations, reviews of student assessment, pre- and post-drawing exercises, and group interviews" (p. 7). Daily "exit tickets" and "retrospective post questionnaires" (p. 7).<br><br>"These items assessed students' perceived ability to read, create, and use data visualizations and conceptions and valuing of art, STEAM, and data visualization before and after the curriculum" (Bertling et al. 2024, p. 7)
	**Was there an evaluation of the role of the arts in enhancing data literacy and, if so, how?**<br><br>The researchers conducted the analysis of qualitative and quantitative data. At the end of the curricula, small group interviews (3-5 students) were conducted for students to "discuss their data-visualization projects and process". A teacher group interview asked teachers to reflect on the challenges and successes of the curriculum. Student data-visualizations, artist statements and drawing exercises were independently coded by two

	researchers who used pre-meeting memos and meetings to compare findings and subsequently conducted a “semiotic analysis of the interconnected imagery” […] Quantitative data were tabulated and translated using descriptive statistics” and then integrated with qualitative findings (Bertling et al. 2024, p. 11).
**Study Population**	**What population groups are being studied in the literature?**<br><br>8th grade students in two suburban middle schools in southeastern USA.<br><br>School population described as: “White (School A: 60%; School B: 78%, Black (School A: 23%; School B: 6%, and Hispanic/Lantinx (School A: 14%; School B: 12%, with less than 5% of students identifying as “other”” (Bertling et al. 2024, p. 7). Students who qualify for free or reduced rate lunch, School A: 32% and School B: 11%.
	**What was the sample size?**<br><br>School A, STEM class: 16 students<br><br>School B, art class one: 16 students<br><br>School B, art class two: 20 students
**Research Setting**	Was it a community-based setting?<br><br>**Was it an educational setting? Yes**<br><br>Was it a healthcare setting?
**Findings/results**	**How have the arts have been used to enhance data literacy?**<br><br>The researchers designed and implemented a transdisciplinary STEAM curriculum to introduce art in STEM classes and data in art classes.
	**Was the evaluation of the role of the arts positive, negative or mixed?**<br><br>Mixed.<br><br>The authors identified both opportunities and challenges in the implementation of the curriculum. **Opportunities:** students expanded their

	conceptions of data visualization, could read and create data visualizations, including arts-based visualizations and understood that data visualizations could be used to "solve community problems" (Bertling et al. 2024, p. 17).<br><br>**Challenges:** the main challenge identified was that more time was needed for instruction and for the student tasks involved in identifying data set patterns. While acknowledging some strengths, the art teacher was concerned that reading data sets was too challenging for the students and "she positioned the curriculum as developmentally inappropriate" (Bertling et al. 2024, p. 19).<br><br>**Negative:** The STEM teacher and the art teacher did not view arts-based data visualizations as an option for inclusion in a future curriculum. The authors suggest that teacher training and additional resources might provide the means to effectively implement the curriculum.<br><br>**Positive:** The authors report that the majority of students in the three classes "broadened their conceptualization of data visualization to include arts-based approaches and were able to create socially and ecologically engaged, arts-based visualizations by the end of the instructional unit" (Bertling et al. 2024, p. 11).<br><br>Ultimately they argue that their research resonates with other studies utilizing "arts-based practices to make sense of and story data" (Bertling et al. 2024, p. 22) and recommend that future research "examine the means by which transdisciplinary third spaces, to include those orientated around arts-based visualization, might be realized and sustained in diverse educational settings" (Bertling et al. 2024, p. 23).<br><br>*End of Bertling et al. 2024*

Bertling et al. 2025

Charting Elements	Associated Questions
**Publication Details**	Bertling, J.G., Galbraith, A., Doss, T.W. and Swartzentruber, R., 2025. Plastic Rivers and E. coli Ice Cream: Visual Metaphors in Middle School Students' Arts-Based Data Visualizations. *Studies in Art Education*, *66*(1), pp.53-77.
**Study ID**	Bertling et al. 2025
**Title**	Plastic Rivers and E. coli Ice Cream: Visual Metaphors in Middle School Students' Arts-Based Data Visualizations
Author(s)	**Who are the authors of the publication?**<br>Bertling, J.G., Galbraith, A., Doss, T.W. and Swartzentruber, R.
Year of Publication	When was the paper/study published?<br>2025
Origin/Country of origin	**Where was the study carried out?**<br>USA
Publication Type	**Is the publication a journal article, book or book chapter, review, opinion paper, grey literature, other?**<br>Journal article
**General Overview of Study**	
Aims/purpose	**What were the aims or purpose of the study?**<br>This study is part of their 2024 larger design based research study (Bertling et al. 2024) with the overall goal of supporting "students' development of arts-based visualizations that communicate important ideas about socially and ecologically engaged data sets" (Bertling et al. 2025, p. 57).<br>The article reports on a part of the broader research by focusing on "students' symbolic and

	metaphoric communication" (Bertling et al. 2025, p. 57).<br><br>**What was the rationale for using the arts?**<br><br>The authors address a research gap around exploring how K-12 students engage in arts-based data visualization. They state that ideas of ideas of data visualization now include contemporary art and design works and this "represents an important new dimension for transdisciplinary art education" (Bertling et al. 2025, p. 54).<br><br>They argue that "data visualizations can communicate through metaphor where two or more concepts are associated, commonly through pictorial means" (Bertling et al. 2025, p. 54).
Increase accessibility<br><br>*Increase engagement*<br><br>*Develop critical thinking skills*<br><br>*Other*	Increase engagement<br><br>Develop critical thinking skills
Methodology	What methodological design was utilized for the study?<br><br>Design based research (DBR) as per Bertling et al. 2024.
**Key findings relating to the role of the arts in data literacy**	
**Methods**	What specific methods (e.g. qualitative, quantitative, mixed methods) were utilized?<br><br>Qualitative. The data sources for this article were confined to "predominantly to summative student assessments" (Bertling et al. 2025, p. 57).<br><br>Data sources: "students' data visualizations and… artist statements […] field notes and student postgroup interviews" (Bertling et al. 2025, p. 57).<br><br>Groupwork - Art class 1: 12 visualizations; Art class 2: 17 visualizations; STEM class: 6 visualizations

	Analysis: researcher analysis of data visualizations, artist statements, interviews, field notes - semiotic analysis.<br><br>**Which art forms were used?**<br><br>Visual arts. Digital collages, traditional art with found objects, papier-mache, tempera paint. Performance art (as described by students): "simulating water quality systems using found and constructed props" (Bertling et al. 2025, p. 59).
	**Was data literacy defined and, if so, how?**<br><br>Data literacy was not defined.
	**Was data literacy measured and, if so, how?**<br><br>Data literacy was not measured.
	**Was there an evaluation of the role of the arts in enhancing data literacy and, if so, how?**<br><br>The focus here is on the analysis of symbolism and metaphor apparent in the students' data visualizations, used to communicate data around social and ecological issues. The visualizations used incorporated digital and traditional art media. The authors discuss various ways to support visual metaphor in K-12 arts-based visualization curricula.
**Study Population**	**What population groups are being studied in the literature?**<br><br>8th grade students in two art classes and one STEM class, as per Bertling et al. 2024.
	**What was the sample size?**<br><br>The sample size is not specified in this article. This is a sub section of the larger Design Based Research project discussed in Bertling et al. 2024, where sample sizes are specified.
**Research Setting**	Was it a community-based setting?<br><br>**Was it an educational setting? Yes**

	Was it a healthcare setting?
**Findings/results**	**How have the arts have been used to enhance data literacy?**<br><br>The focus is more on the semiotics of students' arts-based data visualizations – how metaphor and symbolism are used in the students' communication of their data stories.
	**Was the evaluation of the role of the arts positive, negative or mixed?**<br><br>Mixed. The work of the students' visual metaphors "were often captivating and seemed capable of inspiring valuable community dialogues" (Bertling et al. 2025, p. 72). However, in spite of not quite aligning with initial educational goals, the authors acknowledge that more literal approaches had merits such as speedy viewer comprehension of the data and more direct communication which might be important "in certain journalistic or street art contexts" (Bertling et al. 2025, p. 72).<br><br>The authors are positive about how the investigation demonstrated that "middle-school students employ a wide range of semiotic approaches, some of which can be unique, complex, and intriguingly ambiguous" (Bertling et al. 2025, p. 72). They regard visual metaphors as a powerful means of "inspiring creative cognition, eliciting strong emotion, making new ideas and paradigms digestible, and provoking fresh insights" (Bertling et al. 2025, p. 73).<br><br>*End of Bertling et al. 2025*

Bhargava, Brea, Palacin, Perovich, Hinson 2022

**Charting Elements**	**Associated Questions**
**Publication Details**	Bhargava, R., Brea, A., Palacin, V., Perovich, L. and Hinson, J. (2022) 'Data Theatre as an Entry Point to Data Literacy', *Journal of Educational Technology & Society*, Vol. 25 Issue 4, 93-108 https://helda.helsinki.fi/server/api/core/bitstreams/c0ae6b3c-911f-4547-99d9-87aa1c42eae6/content

**Study ID**	Bhargava et al. 2022
**Title**	Data Theatre as an Entry Point to Data Literacy
Author(s)	**Who are the authors of the publication?**<br><br>Bhargava, R., Brea, A., Palacin, V., Perovich, L. and Hinson, J.
Year of Publication	When was the paper/study published?<br><br>2022
Origin/Country of origin	**Where was the study carried out?**<br><br>USA
Publication Type	**Is the publication a journal article, book or book chapter, review, opinion paper, grey literature, other?**<br><br>Journal article
**General Overview of Study**	
Aims/purpose	**What were the aims or purpose of the study?**<br><br>To introduce "data theatre" (p. 93) that centers on social justice and liberation.<br><br>To build on, expand and complement "our larger body of work building arts-based invitations for the data literacy learner" (p.95).<br><br>"In this paper, we explore theatre, an arts-method, as a promising approach to address the broader need for new paths into speaking the language of data" (p.95)<br><br>"…our driving motivation: exploring how participatory theatre can help us build novel introductions to data that center on justice" (p.95).<br><br>"…our goal to create participatory spaces that bring people together to learn how to speak and embody data, and leverage this knowledge towards goals within their communities" (p.96).<br><br>**What was the rationale for using the arts?**<br><br>The authors set out a context where data gathering and analysis has been dominated by quantitative methods and technology and argue that qualitative, hands-on,

	participatory methods, such as participatory theatre can be introduced to engage with, and represent data.<br><br>"These methods [arts-based] offer learners an opportunity to tap into their kinesthetic intelligence by using their body to express or understand ideas, concepts, and experiences (Lowenfield, 1957)"(p. 95).<br><br>"We offer the activities as an alternate entryway to building a critical data literacy, one that builds on processes rooted in questions of justice and equity, decentering technology and inviting sets of learners who are not engaged through current approaches" (p.105).
Rationale for Using the Arts in Data Literacy Context Choices:<br><br>*Increase accessibility*<br><br>*Increase engagement Yes*<br><br>*Develop critical thinking skills Yes*<br><br>*Other: support education*<br><br>*support embodied learning*	
**Methodology**	**What methodological design was utilized for the study?**<br><br>Case study
**Key findings relating to the role of the arts in data literacy**	
**Methods**	What specific methods (e.g. qualitative, quantitative, mixed methods) were utilized?<br><br>Mixed<br><br>Qualitative: arts-based theatre, interviews, reflection, dialogue.<br><br>Use of quantitative data sets<br><br>The group developed movements to represent both quantitative and qualitative data in the handouts they received.<br><br>**Which art forms were used?**<br><br>Participatory theatre: embodiment, gesture, mime<br><br>movement/dance, storytelling and<br><br>""puppeting" p.98 "Puppeting: This activity was inspired by image theatre, where participants "sculpt" bodies to embody perspectives" (p. 98)

	**Was data literacy defined and, if so, how?**<br><br>"We utilize a four-part definition for "data literacy," engaging components related to acquiring data, processing and analyzing data, representing data, and storytelling with data for some purpose (Bhargava & D'Ignazio, 2015)" (p.93)<br><br>"The approach of critical data literacy offers us theoretical framing within which to situate our activities, and a set of related projects that our work connects to" (p.96).
	**Was data literacy measured and, if so, how?**<br><br>Not discussed. The authors refer to initial evidence of change in engagement, as below.<br><br>Qualitative findings were presented. "we see data theatre as a promising approach that could help engage a critical frame of mind about data itself, though this work did not engage critique of data's use directly" (p. 104)
	**Was there an evaluation of the role of the arts in enhancing data literacy and, if so, how?**<br><br>The researchers write about their observations and interpretations around arts-based theatre and data literacy.<br><br>"Specifically related to RQ1 - Literacy, we saw initial evidence that participants were engaging with some core areas of data literacy: encodings, representation, and editorial choices in storytelling" (p.100).<br><br>"The two puppeting examples…indicate that participants were exploring a variety of modes for representing data in physical form" (p.100).<br><br>Using the arts can "rehumanize data and potentially build empathy and engagement for participants, but we are left with more questions about impacts for the audience" (p.104).
**Study Population**	**What population groups are being studied in the literature?**<br><br>Graduate students and theatre undergraduates (p.97)
	**What was the sample size?**<br><br>Workshop 1-Exploring Movement: 15 graduate students (in-person)

	Workshop 2-Prototype Data Theatre: 10 theatre undergraduates (on Zoom)<br><br>Workshop 3-In-Class Data Theatre<br><br>12 theatre undergraduates (p.97)
**Research Setting**	Was it a community-based setting?<br><br>Was it an educational setting? Yes, "higher education setting" (p.105).<br><br>Was it a healthcare setting?
**Findings/results**	**How have the arts have been used to enhance data literacy?**<br><br>"Data theatre offers one path to making data more understandable, particularly for learners without access to technology, or who do not take to spreadsheets. […] Theatre based introductions to data literacy can be an effective entry point into data literacy that complements other approaches and potentially lowers barriers to certain content based on their embodied nature" (p.105).
Was the evaluation of the role of the arts positive, negative or mixed?	**Was the evaluation of the role of the arts positive, negative or mixed?**<br><br>Positive in terms of empowering participants to engage with data and enhancing data literacy skills.<br><br>"Our initial workshops and prototype activities suggest that data theatre can introduce participants to several core data literacy skills - reading data, picking representations, and creating stories" (p.103).<br><br>"We find early evidence that the data theatre approach is effective at helping participants build some data literacy skills without introductions to statistics and computational tools, reflect critically on datasets and their intended use, and engage in emotionally meaningful embodied performance of data stories" (p. 105).<br><br>*[End of Bhargava, Brea, Palacin, Perovich, Hinson 2022]*

Bhargava and D’Ignazio 2015

**Charting Elements**	**Associated Questions**
**Publication Details**	Bhargava, R. and D’Ignazio, C. (2015) ‘Designing tools and activities for data literacy learners’, *Workshop on data literacy, Webscience 2015,* n.p. https://www.media.mit.edu/publications/designing-tools-and-activities-for-data-literacy-learners/
**Study ID**	Bhargava and D’Ignazio 2015
**Title**	Designing Tools and Activities for Data Literacy Learners
Author(s)	Who are the authors of the publication?<br><br>Bhargava, R. and D’Ignazio, C.
Year of Publication	**When was the paper/study published?**<br><br>2015
Origin/Country of origin	**Where was the study carried out?**<br><br>USA
Publication Type	Is the publication a journal article, book or book chapter, review, opinion paper, grey literature, other?<br><br>Short Paper
**General Overview of Study**	
Aims/purpose	**What were the aims or purpose of the study?**<br><br>“This paper outlines a basic definition of data literacy and uses it to analyze the tools in this space. Based on this analysis, we propose a set of pedagogical design principles to guide the development of tools and activities that help learners build data literacy” (p.1 of PDF np). The authors provide a case study of tools and activities designed to teach data literacy.<br><br>The aim of the study was to propose “a set of pedagogical design principles to guide the development of tools and activities that help learners build data literacy” (first page abstract) and to share a case study of a tool (Wordcounter) built using these principles.<br><br>**What was the rationale for using the arts?**

	The context provided alludes to a rationale for using the arts when engaging with data:<br><br>“Arts-based activities have been used as an introduction to information in an attempt to bring a playful approach to working with data [4]” (p.2 of PDF np).<br><br>It was also noted that its use of song lyrics “builds on a rich tradition of analysing music lyrics as data” (third page).The arts were seen to be inviting (one of their design) principles: “Wordcounter is inviting learners because….contemporary society is struggling with” (fourth page np).
Rationale for Using the Arts in Data Literacy Context Choices:<br><br>*Increase accessibility*<br><br>*Increase engagement*<br><br>*Develop critical thinking skills*	Increase accessibility<br><br>Increase engagement<br><br>Develop critical thinking skills
Methodology	What methodological design was utilized for the study?<br><br>Case study design
**Key findings relating to the role of the arts in data literacy**	
**Methods**	What specific methods (e.g. qualitative, quantitative, mixed methods) were utilized?<br><br>Mixed. Literature review, development of tool and activities, quantitative text analysis using WordCounter.<br><br>**Which art forms were used?**<br><br>Lyrics analysis, songwriting, drawing/sketching<br><br>Sketching: “to create a sketch of visual presentation of their story to share with peers for feedback” (fourth page, np).<br><br>Music: Analysis of music lyrics and songwriting: “Other groups use the data as input for a generative creative process……..from ten artists” (fourth page, np)
	**Was data literacy defined and, if so, how?**<br><br>Yes, they use a definition as they have used it in other publications and elaborate on what it involves: “For our purposes, data literacy includes the ability to read, work

	with, analyse and argue with data. Reading data involves understanding what data is, and what aspects of the world it represents. Working with data involves creating, acquiring, cleaning, and managing it. Analyzing data involves filtering, sorting, aggregating, comparing, and performing other such analytic operations on it. Arguing with data involves using data to support a larger narrative intended to communicate some message to a particular audience" (p.1 of PDF np)
	**Was data literacy measured and, if so, how?**<br><br>Not discussed. The case study is about the development of a prototype – tools for educators
	**Was there an evaluation of the role of the arts in enhancing data literacy and, if so, how?**<br><br>There is only one mention of evaluation in the brief discussion section: "We evaluated this example tool [WordCounter] and activity [analyse music lyrics and create a sketch as a visual representation of their story] in terms of how it fulfils our design principles, and also how it helps build our definition of core data literacy skills in learners" (p.4 of PDF np).<br><br>"While just a prototype, it strongly suggests that following these principles can achieve outcomes that increase data literacy along the axes we have defined. There are certainly opportunities to expand this tool and activity to be further aligned with our design principles" (Page 4 of PDF, np).
**Study Population**	**What population groups are being studied in the literature?**<br><br>Undergraduate students and graduates. "We used WordCounter and the accompanying lyrics anal-ysis activity with two sets of undergraduate and graduate learners in data storytelling courses. One group included undergraduate journalism majors, while the other included students from a mix of degree programs at both undergraduate and graduate levels" (p.4 of PDF np).
	**What was the sample size?**<br><br>Not specified.
**Research Setting**	Was it a community-based setting?<br><br>Was it an educational setting? Yes.<br><br>Was it a healthcare setting?

**Findings/results**	**How have the arts have been used to enhance data literacy?**<br><br>The discussion is more about testing how the tools and activities might support data literacy in the future.<br><br>No direct measure but the arts were used to support the design principles around being "inviting" and researchers stated that the prototype suggests that the tool supports the proposed principles.
Was the evaluation of the role of the arts positive, negative or mixed?	**Was the evaluation of the role of the arts positive, negative or mixed?**<br><br>The evaluation was more about the design principles and expansion of the prototype that the researchers developed:<br><br>"This case presents a first pass at implementing the design principles ["a focused tool, a guiding tool, an inviting tool, an expandable tool" p.3] we proposed for tools that are built for data literacy learners. While just a prototype, it strongly suggests that following these principles can achieve outcomes that increase data literacy along the axes we have defined. There are certainly opportunities to expand this tool and activity to be further aligned with our design principles" (p.4 of PDF np).<br><br>*[End of Bhargava and D'Ignazio 2015]*

Bhargava & D'Ignazio 2017 Data Sculptures

**Charting Elements**	**Associated Questions**
**Publication Details**	Bhargava, R., & D'Ignazio, C. (2017, June). *Data sculptures as a playful and low-tech introduction to working with data* [Paper presentation]. Designing Interactive Systems, Edinburgh, Scotland. https://hdl.handle.net/1721.1/123453
**Study ID**	Bhargava & D'Ignazio 2017
**Title**	Data sculptures as a playful and low-tech introduction to working with data
Author(s)	**Who are the authors of the publication?**<br><br>Bhargava, R., & D'Ignazio, C.

Year of Publication	**When was the paper/study published?**<br><br>2017
Origin/Country of origin	Where was the study carried out?<br><br>U.S.A.
Publication Type	Is the publication a journal article, book or book chapter, review, opinion paper, grey literature, other?<br><br>Conference paper
**General Overview of Study**	
Aims/purpose	**What were the aims or purpose of the study?**<br><br>The authors use three case study examples from ten years of experience of using data sculptures in educational and corporate settings to support their argument that "[d]ata sculptures provide an opportunity to empower more diverse audiences to tell stories with data that can create change in the world around them" (third page).<br><br>**What was the rationale for using the arts?**<br><br>The authors argue that people, particularly novices, experience barriers to creating stories from data as , for example, "technical language and digital technologies" (second page np) can be intimidating for the learner.<br><br>"We argue that the activity of creating low-tech physicalizations of data is particularly well-suited to introduce novices to the process of finding stories in data and telling these stories to others" (second page np).<br><br>The authors propose that the **"**physical objects we create embody our learnings, and serve as physical foils which others can react to and reflect on. The interactive dialog through and with these objects is a powerful path to learning" (third page).<br><br>The authors argue that approaches using data sculptures made up of physical craft materials "present an opportunity to create something in a physical language that is familiar to the learner" (third page).
Rationale for Using the Arts in Data Literacy Context Choices:<br><br>*Increase accessibility*<br><br>*Increase engagement*	Increase accessibility<br><br>Increase engagement<br><br>Creative learning opportunities

Develop critical thinking skills<br>*Other: creating learning opportunities*<br>*Enhancing creative interaction with data*	Enhancing creative interaction with the data
Methodology	Discussion of three case studies – Case by case descriptions - reflections on the authors' hands-on experience in multiple learning settings over 10 years
**Key findings relating to the role of the arts in data literacy**	
**Methods**	**What specific methods (e.g. qualitative, quantitative, mixed methods) were utilized?**<br>Data sculptures – multiple learning settings<br>"First, we discuss doing short 5-minute building activities in professional development workshops with non-expert audiences. Second, we look at creations made by undergraduate and graduate students in week-long data sculpture "sketching" exercises. Third, we look at some nascent work introducing data sculptures to elementary school students in classroom settings" (second and third pages).<br>**Which art forms were used?**<br>Data sculptures; data sculpture sketches
	**Was data literacy defined and, if so, how?**<br>Data literacy is referred to but not formally defined
	**Was data literacy measured and, if so, how?**<br>Data literacy is not measured
	**Was there an evaluation of the role of the arts in enhancing data literacy and, if so, how?**<br>The researchers reflect on their hands-on experience (over ten years) of using data sculptures in corporate and educational settings in order to make a case for the importance of using data sculptures as a pathway to identifying and telling stories with data in these contexts.
**Study Population**	What population groups are being studied in the literature?

	The authors refer to three specific groups: corporate staff, higher education students and elementary school students.
	What was the sample size?<br><br>Not specified
**Research Setting**	Was it a community-based setting? Corporate<br><br>Was it an educational setting? Elementary school and higher education settings<br><br>Was it a healthcare setting?
**Findings/results**	**How have the arts have been used to enhance data literacy?**<br><br>Data sculptures were used to introduce participants to finding stories in data and telling data stories.
Was the evaluation of the role of the arts positive, negative or mixed?	**Was the evaluation of the role of the arts positive, negative or mixed?**<br><br>Positive.<br><br>"This brings us back to approaches like Freire's, which thinks about learning and literacy as a path to empowerment rather than simply skill-building. Our work strongly suggests that data sculptures are particularly well-suited to help novices overcome the barriers to learning and empower them to work with data to solve the problems they think are most important" (Sixth page np).<br><br>"Data sculptures provide an opportunity to empower more diverse audiences to tell stories with data that can create change in the world around them"(Sixth page np).

Bhargava, Kadouaki, Bhargava, Castro and D'Ignazio 2016

**Charting Elements**	**Associated Questions**
**Publication Details**	Bhargava, R., Kadouaki, R., Bhargava, E., Castro, G. and D'Ignazio, C. (2016) 'Data murals: Using the arts to build data literacy', *The Journal of Community Informatics*, 12(3), 197-216.<br><br>https://www.media.mit.edu/publications/data-murals-using-the-arts-to-build-data-literacy/

**Study ID**	Bhargava et al., 2016
**Title**	Data murals: Using the arts to build data literacy
Author(s)	Rahul Bhargava, Ricardo Kadouaki, Emily Bhargava, Guilherme Castro, and Catherine D'Ignazio.
Year of Publication	2016
Origin/Country of origin	Brazil
Publication Type	Journal article
**General Overview of Study**	
Aims/purpose	**What were the aims or purpose of the study?**<br><br>The authors outline a case study example of “a Popular Education-inspired approach to building participatory and impactful data literacy using a set of visual arts activities with students” (p.197)<br><br>“This paper documents one of our efforts to promote data literacy in Brazil with an arts-centric approach” (p.198).<br><br>**What was the rationale for using the arts?**<br><br>“technology-centric interventions to building data literacy have missed a crucial opportunity to look outside of the digital world for approaches and inspirations. (p.198)<br><br>The project was building on “a rich tradition of using the arts and the lived experiences of participants as an invitation for civic engagement” (p.198)<br><br>p.200 and 201 describe examples of this long history including the history of mural art and theatre of the oppressed.<br><br>They argue that the arts provide a means to engage with data in creative ways, rooted in lived experience, rather than through technology, which has predominated in public school curriculums.<br><br>“We offer this case study as an example of how to build data literacy in novel and creative ways built on the needs, affordances, history,

	and context of the community one is working with" (p.199).
Rationale for Using the Arts in Data Literacy Context Choices:<br><br>*Increase accessibility*<br><br>*Increase engagement*<br><br>*Develop critical thinking skills*<br><br>*Other*	* p.209 "A car with an amplifier its roof was sent around the neighborhood to play a recorded announcement that everyone was invited to join the mural painting activity at Plug.<br><br>* p.209 "School staff invited students across all theNucleos to join the painting process the next day".<br><br>* p.203 "to improve capacity to read, understand, and synthesize data into insights".<br><br>Have fun! P.203 "to build the belief that playing with data to tell a story can be fun."
Methodology	**What methodological design was utilized for the study?**<br><br>Case study<br><br>"The researchers (Bhargava & Bhargava), desired to test their hypothesis that this type of arts-based intervention could increase participant data literacy, build and reinforce connections among participants, and act as a catalyst for further conversation about the topic being explored" (p.202).
**Key findings relating to the role of the arts in data literacy**	
**Methods**	**What specific methods (e.g. qualitative, quantitative, mixed methods) were utilized?**<br><br>Mixed methods. Qualitative (informal interviews), revisiting the field for more longitudinal findings.<br><br>Design, arts-based methods (data sculpture, data murals, storytelling)<br><br>Data sets (quantitative) e.g. population numbers<br><br>Which art forms were used?<br><br>Visual (mural, canvas painting, data sculpture) storytelling, music (background) film (of process)<br><br>* (circus arts mentioned as an area of focus in the school)
	**Was data literacy defined and, if so, how?**<br><br>Mentioned that "the wide variety suggests the definition is still being fleshed out" (p.198)

	“For this case study, we build on our previous work (Bhargava & D'Ignazio 2015), which defines data literacy as the ability to read, work with, analyze and argue with data” (p. 198).
	**Was data literacy measured and, if so, how?**<br><br>No exact measurement but qualitative interviews indicated “The experience of creating the data mural built students' data literacy in various ways. (p.211) e.g. “interviews conducted revealed critical thinking about the data gathering process (p.211)<br><br>“Unfortunately, logistical constraints on-site prevented us from doing a thorough pre- and post-survey. Instead, informal interviews were used with students and staff the day after the mural was painted to assess short-term outcomes. To look at longer term outcomes, researchers remained in contact with staff on-site and checked in during the months following the data mural process, and again a year after the project was complete. This is less than ideal, but provides significant qualitative data to inspect for findings” (p. 211).
	**Was there an evaluation of the role of the arts in enhancing data literacy and, if so, how?**<br><br>Qualitative interviews indicated that the arts enhanced engagement with data literacy: “’Working with data with arts – is very interesting… to turn the data into art. Another responded to the question of if they learned anything by saying ‘I’ve never had this experience to play with data and art. And data can be translated into art.’" (P.213)<br><br>“The experience of creating the data mural built students' data literacy in various ways” (p.211).<br><br>Interviews revealed that the project encouraged critical thinking about how information is gathered, what might be missing from the data, and how data can be used to influence change.
**Study Population**	**What population groups are being studied in the literature?**<br><br>Students aged 16 to 21 from Plug Minas school in Belo Horizonte<br><br>School children took part in the data design workshops and mural development: “Plug Minas (hereafter referred to as "Plug") is a state sponsored school that promotes education through innovative educational methodologies, guided by values like leadership and

	digital literacy. Acting as a complement to the regular public school system, Plug has approximately 1,300 students aged between 14 and 24 years old." (p.202) This is an affluent school in an affluent part of Brazil.<br><br>No further information provided on gender, ethnicity, social group.
	**What was the sample size?**<br><br>Total not specified.<br><br>"The story-finding and design workshops involved almost 20 student participants, the two researchers, three Plug staff, and three Escritorio staff. The 2-day process were held on-site in a large auditorium…Approximately 50 people participated in the mural painting" (p.203).
**Research Setting**	Was it a community-based setting?<br><br>*****some outreach noted<br><br>Was it an educational setting? * School is a state sponsored school that promotes education through innovative educational methodologies, guided by values like leadership and digital literacy" (p.202).<br><br>Was it a healthcare setting?
**Findings/results**	**How have the arts have been used to enhance data literacy?**<br><br>* used to enhance non-technical, creative approaches to data literacy<br><br>* used to increase fun<br><br>* to increase participation and connections<br><br>"The arts approach appealed to participants in a way that simply looking at a spreadsheet never does, to the point where they described it as "play"" (p.213).<br><br>"Through observation and interview, we showed that participants in the Data Mural process demonstrated increased data literacy, while at the same time having fun! The students used creative tools to turn data into meaningful stories about their identity. Plug students and staff also walked away with new connections across the Nucleos" (pp.214-215).

Was the evaluation of the role of the arts positive, negative or mixed?	**Was the evaluation of the role of the arts positive, negative or mixed?**<br><br>Broadly positive - "As a result of the project data literacy among participants increased, and the project initiated a sustained interest within the school community in using data to tell stories and create social change." (abstract P.197)<br><br>"An important part of data literacy is feeling less intimidated by data. Feeling that you have the power to understand and transform the information into what you want it to be. In terms of accomplishing this outcome, the process was successful" (p. 211).<br><br>"In addition to the students, the staff felt like the process gave them the tools to use data in new and interesting ways in the future" (p. 212).<br><br>"The arts approach appealed to participants in a way that simply looking at a spreadsheet never does, to the point where they described it as "play"" (p.213).<br><br>"We found that the Data Mural process increased students' comfort with data, created a sustained interest from students in using data to tell stories in creative ways, and reinforced connections across the Nucleos. The use of playful, relatable data resonated with the participants and created interest in a traditionally dry topic" (p.214).<br><br>*[End of Bhargava, Kadouaki, Bhargava, Castro and D'Ignazio 2016]*

Blackburn 2015

**Charting Elements**	**Associated Questions**
**Publication Details**	Blackburn, G. (2015), 'Effectiveness of eLearning in statistics: Pictures and stories', *E-Learning and Digital Media,* Vol. 12 Issue 5-6 Pages 459-480 https://doi.org/10.1177/2042753016653704
**Study ID**	Blackburn 2015
**Title**	Effectiveness of eLearning in statistics: Pictures and stories

Author(s)	**Who are the authors of the publication?**<br><br>Greg Blackburn
Year of Publication	**When was the paper/study published?**<br><br>2015
Origin/Country of origin	**Where was the study carried out?**<br><br>Author: Germany<br><br>Study reflects on experiences of UQ, University of Queensland, Australia.
Publication Type	**Is the publication a journal article, book or book chapter, review, opinion paper, grey literature, other?**<br><br>Journal article
**General Overview of Study**	
Aims/purpose	**What were the aims or purpose of the study?**<br><br>“The study investigates (1) the effectiveness of using eLearning-embedded stories and pictures in order to improve learning outcomes for students and (2) how universities can adopt innovative approaches to the creation of Problem-Based Learning (PBL) resources and embed them in educational technology for teaching domain-specific content, such as statistical literacy – a widely documented difficult subject for students to master” (abstract, p. 459).<br><br>“The research question for this study is:<br><br>To what extent and in what ways does using eLearning embedded fictional real-life context stories allow students to perform differently than those exposed to pre-course materials only?<br><br>The hypotheses for the study are:<br><br>H1: By using eLearning-embedded fictional real-life context stories, these students will perform better than those exposed to pre-course materials.<br><br>H2: By being exposed to pre-course materials, these students will perform better than their counterparts who use eLearning-embedded fictional real-life context stories and pictures (p.464)”.<br><br>**What was the rationale for using the arts?**

	"ECON 1310 is an introductory quantitative analysis course with approximately 800 enrolled students per semester" (p.459).<br><br>The arts are not specifically mentioned but storytelling and pictures were used to show practical relevance and contextualise abstract concepts: "Since understanding economic theory and, in particular, the abstract mathematical concepts in economic statistics, can be difficult for students, the course was redesigned to be interactive and delivered via eLearning technology, as well as show the practical relevance of learning statistics through explanations using stories that revolve around a fictional fish farming business. Abstract statistical concepts are contextualized through characters, such as a seagull, a pelican and Freaky Fish" (p.460).<br><br>The author notes that the students previously considered the course boring and frustrating (p. 460).
Rationale for Using the Arts in Data Literacy Context Choices:<br><br>*Increase accessibility*<br><br>*Increase engagement*<br><br>*Develop critical thinking skills*<br><br>*Other: show practical relevance of statistics.*	
Methodology	**What methodological design was utilized for the study?**<br><br>Unclear. Evaluation?<br><br>"This study is aimed at the evaluation of the pedagogical effectiveness of employing a set of pictorial icons with - based teaching method in improving outcomes for students" (p. 464).
**Key findings relating to the role of the arts in data literacy**	
**Methods**	**What specific methods (e.g. qualitative, quantitative, mixed methods) were utilized?**<br><br>Mixed methods. Quantitative (pre and post questionnaire) and qualitative data gathering and analysis (p.466). Questionnaires.<br><br>**Which art forms were used?**<br><br>Visual arts/fictional storytelling<br><br>Pictures (freaky fish, seagull, pelican).

	**Was data literacy defined and, if so, how?**<br>Data literacy is not mentioned but the explanation below follows a reference to statistical literacy:<br>"MacGillivray and Pereira-Mendoza (2011: 110) argue that 'the learning and teaching of statistical thinking requires gradual building up of concepts, understanding and skills, in a coherent, consistent and cumulative way that engages students in real contexts and authentic learning experiences'" (p.460).
	**Was data literacy measured and, if so, how?**<br>"Participating students completed a pre-test online in order to test their understanding of fundamental statistical concepts before taking the course. At the end of the term, participating students completed a post-test online in order to test for increases in statistical literacy" (p.465).
	**Was there an evaluation of the role of the arts in enhancing data literacy and, if so, how?**<br>The e-learning programme using pictorial icons was evaluated by the researcher who discusses the qualitative data analysis. See below.
**Study Population**	**What population groups are being studied in the literature?**<br>University students
	**What was the sample size?**<br>Participants for the study included 385 undergraduate first-year university students enrolled in an introductory statistics course (ECON 1310 Quantitative and Economic and Business Analysis) (p.464).
**Research Setting**	Was it a community-based setting?<br>Was it an educational setting? Yes –University of Queensland<br>Was it a healthcare setting?
**Findings/results**	**How have the arts have been used to enhance data literacy?**<br>"…an innovative approach has been introduced to teaching undergraduate statistics by employing

	eLearning tools to embed pictorial icons within a problem learning environment to connect learning with real-world practices, and this has resonated with students" (p.475).
Was the evaluation of the role of the arts positive, negative or mixed?	**Was the evaluation of the role of the arts positive, negative or mixed?**<br><br>Positive overall with challenges noted.<br><br>"…based on the data, the teaching method appears to have a positive influence on student understanding of subject matter" (p. 474).<br><br>"The qualitative data demonstrate that students largely enjoyed the real-world experience of learning statistics in the eLearning environment and related to the pictorial icons. It was also found that student pass rates have risen from 70% in 2009 to 87% in 2010 as a result of this pedagogy. More recently it increased to 90% in 2011 and 2012" (p.475).<br><br>"…two challenges unique to statistics have emerged. First, the cumulative approach was a challenge for students to keep up with whilst completing assignments. Second, instructors faced the challenges of instructing students from wide-ranging disciplinary backgrounds with varying degrees of prior mathematical knowledge" (p.475).<br><br>"Moving away from a traditional teacher-centred teaching approach to a student-focused approach embedded in technology has helped influence,motivate and inspire students, who can apply theory to function effectively in the corporate environment as independent and proactive professionals" (p.475).<br><br>*[End of Blackburn 2015]*

Bowler et al. 2020

**Charting Elements**	**Associated Questions**
**Publication Details**	Bowler, L., Aronofsky, M., Milliken, G., & Acker, A. (2020). Teen engagements with data in an after-school data literacy programme at the public library. In *Proceedings of ISIC, the Information Behaviour Conference, Pretoria, South Africa, 28-1 October, 2020. Information Research, 25*(4), paper isic2015. Retrieved from http://InformationR.net/ir/25-4/isic2020/isic2015.html (Archived by the Internet

	Archive at https://bit.ly/3a6DBwv)<br>https://doi.org/10.47989/irisic2015
**Study ID**	Bowler et al. 2020
**Title**	Teen engagements with data in an after-school data literacy programme at the public library
Author(s)	Who are the authors of the publication?<br>Leanne Bowler, Manuela Aronofsky, Genevieve Milliken, and Amelia Acker.
Year of Publication	When was the paper/study published?<br>2020
Origin/Country of origin	Where was the study carried out?<br>USA: It is based on the . "*Exploring Data Worlds at the Public Library*, a three-year empirical research study that took place at a public library in the United States" (no page numbers- p. 2 in doc)
Publication Type	Is the publication a journal article, book or book chapter, review, opinion paper, grey literature, other?<br>Proceedings of ISIC, the Information Behaviour Conference, Pretoria, South Africa, 28-1 October, 2020. *Information Research, 25*(4),
**General Overview of Study**	
Aims/purpose	**What were the aims or purpose of the study?**<br>The paper notes that data empowerment has important developmental implications and, "With this in mind we ask, how can libraries find ways to help teens develop the intrinsic motivation, attention, interest and effort needed to be more fully self-determining data producers and consumers?"(second page np) The study proposed to examine these questions by studying indicators of *engagement* in data literacy workshops.

	**What was the rationale for using the arts?**<br><br>There was no explicit rationale given for using the arts. The study looked at 11 data literacy workshops which had been designed as part of the *Exploring Data Worlds at the Public Library* project. A number of these workshops used artistic methods such as arts&crafts and data visualisation such as the following two workshops:<br><br>“*Data Keychains* – Visualising data subjectivity through keychains created from personal information.<br>4. *Data Postcards* – Creating data visualisations of teens’ daily data” (Tenth page, figure 2, np).<br><br>The following noted on p.11<br><br>“the value of play”<br><br>“making personal connections”<br><br>“cognitive breakthroughs, interactivity and embodied learning”<br><br>*Increase accessibility<br><br>*Increase engagement<br><br>*Develop critical thinking skills
Rationale for Using the Arts in Data Literacy Context Choices:<br><br>*Increase accessibility*<br><br>*Increase engagement*<br><br>*Develop critical thinking skills*<br><br>*Other*	
Methodology	**What methodological design was utilized for the study?**<br><br>“The study examines two data sources (observation notes and feedback forms) from the *Exploring Data Worlds* research project: (1) the observation notes from 27 data literacy workshops on 11 unique data topics, held at the public library with 95 teens, and, (2) feedback forms completed by teen participants following each workshop” (p.9 on doc).

Key findings relating to the role of the arts in data literacy	
**Methods**	What specific methods (e.g. qualitative, quantitative, mixed methods) were utilized?<br><br>Mixed method (qualitative and quantitative, analysing observation notes and feedback forms)<br><br>Which art forms were used?<br><br>Data visualisations; arts and crafts
	**Was data literacy defined and, if so, how?**<br><br>Yes and the specific skills included in the field of data science also noted:<br><br>"Data literacy is often defined as the '*ability to read, work with, analyse and argue with data as part of a larger inquiry process*' (D'Ignazio and Bhargava, 2016). In the field of Information Science, the definition of data literacy includes skills necessary for the curation and preservation of data" (Lyon and Brenner, 2015). (p.3 in doc)<br><br>However, it also notes that data literacy can be difficult to define:<br><br>" the concept of data literacy is under-theorised and lacking a single definition. This is not surprising, as even in academic literature there is an agreement that the nature of data itself can be hard to define (Borgman, 2015) " (p. 4 in doc)<br><br>They also noted that data literacy is contextual, interpretivist and involves ideological and ethical dimensions.<br><br>"In their thematic analysis of the critical data literacy discourse, Špiranec et al (2019) highlight the contextual and interpretive nature of data, it's ideological foundations and ethical dimensions" (p.6 in doc)
	**Was data literacy measured and, if so, how?**<br><br>No, there was not a specific measurement of data literacy.<br><br>"We acknowledge that our study does not measure learning outcomes nor was it intended to do so." (p. 16 in doc)<br><br>The focus of the paper was on indicators of engagement in data literacy workshops.

	" focusing on the specific features associated with engagement with data, asking the question, *what do teens find engaging and fun (or, boring and not worthy of their attention) in their interactions with data at the library?"* (p. 9 in doc)<br><br>Meaningful engagement was seen to be high based on feedback forms:<br><br>"Based on evidence from the feedback forms, responses to the workshops from teens were overwhelmingly positive, with 95.87% giving the data workshops a *thumbs up*. Two percent gave the workshops a *thumbs down* and two percent left this question blank. The average score for 23 workshops, on a scale of one to five where five is best, was 4.44. In addition, 88.8%, of the teens surveyed reported being *happy* or *most happy* with their experiences" (p, 12 in doc).
	**Was there an evaluation of the role of the arts in enhancing data literacy and, if so, how?**<br><br>While there was not an explicit evaluation of the role of the arts, an arts-based workshop was seen to increase a sense of personal engagement with the date:<br><br>"Across all workshops, engagement was notably higher when the teen participants were able to make a personal connection to data during the activity, finding themselves or their community in the data […] In addition, a personal connection to the subject matter not only increased participation levels but also raised the quality and depth of discussions that took place between the participants and facilitators. For example, the popular *Data Keychains* activity asked participants to visualise their personal data and identities with coloured beads in order to make a keychain that seemingly encodes themselves (i.e. coloured beads could represent a teen's age, gender, likes and dislikes) (p.13 in doc).
**Study Population**	**What population groups are being studied in the literature?**<br><br>"Approximately 95 teens participated in 27 after-school, drop-in workshops . All participants were teens between the ages of 14 to 17 years. Of the 95 participants, 52 identified as female and 37 as male, with six leaving the

	open-ended question about gender blank (‘*I identify my gender as*…’). The workshops were held in a teen services area at an urban, mid-sized city in the North-Eastern United States, and each was designed and facilitated by members of the *Exploring Data Worlds* research team, with library staff in attendance”<br><br>(p.9 in doc)
	**What was the sample size?**<br><br>95 teens
**Research Setting**	Was it a community-based setting? * public library<br><br>Was it an educational setting? Informal learning setting in public library<br><br>Was it a healthcare setting?
**Findings/results**	**How have the arts have been used to enhance data literacy?**<br><br>No explicit findings related to the arts but engagement in the workshops (which included arts-based activities) was seen to be enhanced by characteristics often enhanced by artistic engagement including:<br><br>“personal connections to data, embodied learning, interactions with data through facilitation techniques (analogy as one such example), opportunities for inquiry and discovery, social arrangements that encourage interaction, and adopting a playful attitude to learning” (p.16 in doc)
Was the evaluation of the role of the arts positive, negative or mixed?	**Was the evaluation of the role of the arts positive, negative or mixed?**<br><br>There was no explicit evaluation of the role of the arts but engagement with the workshops (which included arts-based elements) was overwhelmingly positive:<br><br>“…responses to the workshops from teens were overwhelmingly positive, with 95.87% giving the data workshops a *thumbs up*. Two percent gave the workshops a *thumbs down* and two percent left this question blank. The average score for 23 workshops, on a scale of one to five where five is best, was 4.44. In addition, 88.8%, of the teens surveyed reported being *happy* or *most happy* with their experiences (p. 12 in doc.)

	[End of Bowler et al. 2020]

DesPortes et al. 2022

**Charting Elements**	**Associated Questions**
**Publication Details**	DesPortes, K., Vacca, R., Tes, M., Woods, P.J., Matuk, C., Amato, A., Silander, M. (2022) 'Dancing with Data: Embodying the Numerical and Humanistic Sides of Data', *Proceedings of International Conference of the Learning Sciences, ICLS 2022*: 305-312. https://repository.isls.org/bitstream/1/8805/1/ICLS2022_305-312.pdf
**Study ID**	DesPortes et al. 2022
**Title**	
Author(s)	**Who are the authors of the publication?**<br>DesPortes, K., Vacca, R., Tes, M., Woods, P.J., Matuk, C., Amato, A., Silander, M.
Year of Publication	**When was the paper/study published?**<br>2022
Origin/Country of origin	**Where was the study carried out?**<br>USA
Publication Type	**Is the publication a journal article, book or book chapter, review, opinion paper, grey literature, other?**<br>Conference paper
**General Overview of Study**	
Aims/purpose	**What were the aims or purpose of the study?**<br>"In this study, we explore how dancing with and about data can support the understanding of mathematical

	concepts such as number sense, contextualization of data, and making meaning with data" (p.306).<br><br>**What was the rationale for using the arts?**<br><br>"Data literacy has been critiqued for focusing mostly on technical skills with disregard for broader concepts like *citizenship* and *empowerment,* which can exacerbate educational and social inequities (D'Ignazio, 2017). A creative data literacy framing that incorporates art production with data practices can attend to these issues (D'Ignazio, 2017) and help students achieve the same rigor in learning as traditional curricula (Bhargava et al., 2016)" (p. 305).<br><br>"The unique embodied practices of dance create opportunities to expand the ways that learners can explore and build understanding with and about data" (p. 305).<br><br>*Increase accessibility<br><br>*Increase engagement<br><br>*Develop critical thinking skills<br><br>*Reduced inequalities
Rationale for Using the Arts in Data Literacy Context Choices:<br><br>*Increase accessibility*<br><br>*Increase engagement*<br><br>*Develop critical thinking skills*<br><br>*Other*	
Methodology	**What methodological design was utilized for the study?**<br><br>"Case study of dance and data" (p. 311).<br><br>"Co-design" (p. 306).<br><br>"Our work takes a grounded cognitive approach (Barsalou, 2010) to explore ways to promote engagement with data. Specifically, we examine how a data-dance context provides opportunities for learners to create embodied metaphors to think about, represent, and communicate about data" (p. 305).
**Key findings relating to the role of the arts in data literacy**	

**Methods**	**What specific methods (e.g. qualitative, quantitative, mixed methods) were utilized?**<br><br>Qualitative<br><br>“We engaged in co-design with one math and one dance teacher in a public charter school in the Midwestern United States” (p. 306).<br><br>“…student group interviews, dance planning materials, and a paired teacher interview. The student group interviews were conducted with each of the five student teams” (p. 306).<br><br>“The dance planning materials were created by each of the student teams and made them connect *claims/supports* from the data to *movements* in their dance. The paired teacher interview was conducted after unit implementation with both the art and math teachers” (p. 306).<br><br>“In the unit, students: (1) picked a topic of interest, (2) interpreted and analyzed data sources on that topic provided by the co-design team, and (3) created a dance in response to the data and their questions grounded in the data. Students performed these dances and presented an artist statement explaining them at an exhibition attended by other students, teachers, parents, and community members” (p. 306).<br><br>**Which art forms were used?**<br><br>Dance
	**Was data literacy defined and, if so, how?**<br><br>Data literacy is not specifically defined.
	**Was data literacy measured and, if so, how?**<br><br>Data literacy was not measured.
	**Was there an evaluation of the role of the arts in enhancing data literacy and, if so, how?**<br><br>The role of dance was evaluated through thematic analysis. One theme is presented in the paper.<br><br>“We collected data from five separate project groups that engaged with data on: (1) *Women’s Rights*—Emily, Gabby, Amber, Cameron, (2) *Minecraft*—Cory, (3) *Animal Populations*—Irene, Debbie, (4) *Mental Health*—

	Margaret, and (5) *Social Media and Mental Health*—Will, Brooke, Ashley. Student names used in the analysis are pseudonyms" (p. 306).<br><br>Following interviews (see methods column):<br>"We engaged in an iterative coding process beginning with open descriptive coding (Saldaña, 2015) that was refined through social moderation (Frederiksen, 1998). For each iteration, conflicts and differences in interpretation were discussed and codes were refined. The codebook was reviewed and checked for clarity with researchers outside of the project team, who discussed the coding of a subset of data" (p. 306).<br><br>"In this paper, we focus on one of our analytic themes, ***embodied data characteristics—****characteristics of the data that students used to create embodied metaphors*. We identified three data characteristics that students used to create embodied metaphors: graph shape & numerical value, data context, and data implications (Table 1)" (p. 306).
**Study Population**	**What population groups are being studied in the literature?**<br><br>One math and one dance teacher and students in a public charter school, Midwestern USA (p. 306)<br><br>"The teachers taught the data-dance unit to eleven 7th grade students who were in both the math and dance classes. The middle school student population is 65% White, 19% Hispanic, and 6% Black—46% of the student population qualifies for free or reduced lunch" (p. 306).
	**What was the sample size?**<br><br>Not specified.
**Research Setting**	Was it a community-based setting?<br><br>Was it an educational setting? Yes<br><br>Was it a healthcare setting?
**Findings/results**	**How have the arts have been used to enhance data literacy?**<br><br>Dance was used as a means to embody metaphorical representations of data.<br><br>"Dance practices fundamentally changed how learners built knowledge with data, including how they examined, reasoned around, related to, and represented the data" (p. 310).

Was the evaluation of the role of the arts positive, negative or mixed?	**Was the evaluation of the role of the arts positive, negative or mixed?**<br><br>Mixed. Positive in term of the learning experience for students with some limitations outlined.<br><br>"Learning experiences, such as creating and performing data dances from both a math and art disciplinary perspective has potential to center tensions on the role of affect in making claims about how the data should feel, how audiences engage in embodied and affective forms of sense-making through kinesthetic empathy, and the use of artistic dance practices that center this affective communication in data visualizations (e.g., music, gestures)" (p. 311).<br><br>"Although this study alludes to a number of important connections between data literacy and embodied learning, there were some limitations. First, the study was situated in the context of contemporary dance, limiting the range of embodied practices. Second, we ran into challenges in understanding learners' affective responses relying mostly on their verbal descriptions instead of embodied emotional responses during the curricular unit due to our limited ability to observe during classroom activities" (p. 311).<br><br>*End of DesPortes et al. 2022*

D'Ignazio and Bhargava 2016

**Charting Elements**	**Associated Questions**
**Publication Details**	D'Ignazio, C., Bhargava, R. (2016). DataBasic: design principles, tools and activities for Data Literacy Learners. *The Journal of Community Informatics*, 12(3), 83—107.
**Study ID**	D'Ignazio & Bhargava 2016
**Title**	DataBasic: design principles, tools and activities for Data Literacy Learners
Author(s)	**Who are the authors of the publication?**<br><br>D'Ignazio, C. and Bhargava, R.

Year of Publication	**When was the paper/study published?**<br><br>2016
Origin/Country of origin	**Where was the study carried out?**<br><br>U.S.A.
Publication Type	**Is the publication a journal article, book or book chapter, review, opinion paper, grey literature, other?**<br><br>Journal article
**General Overview of Study**	
Aims/purpose	**What were the aims or purpose of the study?**<br><br>"This paper proposes a set of pedagogical design principles for tool development to support data literacy learners" (p. 83).<br><br>**What was the rationale for using the arts?**<br><br>The authors "assert that most data tools for novices are designed for users, not learners, and privilege the production of quick visuals at the expense of supporting a learning process" (p. 84). They propose alternative design criteria "guided by strong pedagogical goals" (p. 84). They describe the online tools they have created to support their argument: "DataBasic a suite of three digital tools, with accompanying participatory activities" (p.) Activities include analysing song lyrics to find and tell stories by creating sketches with crayons and paper to visualize and represent their stories [Wordcounter] and creating imaginary song lyrics (SameDiff).
Rationale for Using the Arts in Data Literacy Context Choices:<br><br>*Increase accessibility*<br><br>*Increase engagement*<br><br>*Develop critical thinking skills*<br><br>*Other*	*Increase accessibility<br><br>*Increase engagement<br><br>*Develop critical thinking skills
Methodology	**What methodological design was utilized for the study?**<br><br>Qualitative and quantitative
**Key findings relating to the role of the arts in data literacy**	

**Methods**	**What specific methods (e.g. qualitative, quantitative, mixed methods) were utilized?**<br><br>"pre- and post-surveys, observation and analysis of generated artifacts to critically assess DataBasic" (p. 95). Workshops.<br><br>**Which art forms were used?**<br><br>Visual art: sketches<br><br>Music: sketches created to represent song lyrics and songwriting
	**Was data literacy defined and, if so, how?**<br><br>Yes: "Building on these existing descriptions, we adopt a multi-faceted definition of data literacy. For our purposes, data literacy includes the ability to read, work with, analyze and argue with data as part of a larger inquiry process" (p. 84).
	**Was data literacy measured and, if so, how?**<br><br>The pre-survey asked participants to rate tools they had used in the past, and about their comfort level in "analyzing text data and data in a table (rated on a Likert scale) […] The post-survey again asked people how comfortable they felt analyzing text data and data in a table" (p. 95). Participants were also asked about each of the three tools and to provide feedback and suggestions.
	**Was there an evaluation of the role of the arts in enhancing data literacy and, if so, how?**<br><br>"We evaluated DataBasic's three tools and their accompanying activities at a workshop in November 2015 at the MIT Media Lab […] We admininstered a pre- and post-survey immediately before and after the workshop" (p. 95).
**Study Population**	**What population groups are being studied in the literature?**<br><br>"undergraduate students and adult learners from non-profit organizations, news organizations and community advocacy groups" (p. 95).
	**What was the sample size?**<br><br>Sample size listed for the evaluation workshop only: 25 people. Age range: 20-65 with the majority aged 20-35.

**Research Setting**	Was it a community-based setting? Yes<br><br>Was it an educational setting? Yes<br><br>Was it a healthcare setting?
**Findings/results**	**How have the arts have been used to enhance data literacy?**<br><br>Hands-on activities such as creating sketches to represent data stories based on popular American song lyrics, and writing hypothetical song collaborations based on musical data samples were used as a means to introduce participants to working with data/arguing with data.
Was the evaluation of the role of the arts positive, negative or mixed?	**Was the evaluation of the role of the arts positive, negative or mixed?**<br><br>Positive in terms of interaction with the tools in general. The authors note that their "high-level evaluation of the three tools supports the idea that learners increased their comfort level with analyzing text data" (p. 96). Mixed in terms of the role of the arts: "Analyzing the sketches [WordCounter] created by the learners shows that they develop a strong ability to argue with data" (p. 98). The authors note that further work is needed "to assess how participants reflect on WordCounter as a learning tool" (p. 99). The SameDiff tool activities involved choosing two musical artists from the sample data and writing a hypothetical collaborative song (also singing it for extra points in the workshop). The authors note that this "activity led to the most laughter and delight of the three workshop activities, we have also struggled the most in matching our learning goals to the activity itself" (p. 104) i.e. introducing concepts of data analysis.<br><br>*End of D'Ignazio & Bhargava 2016*

D'Ignazio and Bhargava 2018

**Charting Elements**	**Associated Questions**
**Publication Details**	D'Ignazio, C., and Bhargava, R. (2018) 'Creative Data Literacy: A Constructionist Approach to Teaching Information Visualization', *Digital Humanities Quarterly*, Vol. 12 Issue 4. https://dspace.mit.edu/handle/1721.1/123473

**Study ID**	D'Ignazio and Bhargava 2018
**Title**	"Creative Data Literacy: A Constructionist Approach to Teaching Information Visualization"
Author(s)	**Who are the authors of the publication?**<br><br>D'Ignazio, Catherine and Bhargava, Rahul.
Year of Publication	When was the paper/study published? 2018
Origin/Country of origin	Where was the study carried out? USA – u/g and graduate courses mentioned:<br><br>"In our undergraduate and graduate courses at MIT and Emerson College," no page numbers (p.4 of PDF)
Publication Type	Is the publication a journal article, book or book chapter, review, opinion paper, grey literature, other?<br><br>Journal:<br><br>*Digital Humanities Quarterly*, Vol. 12 Issue 4.
**General Overview of Study**	
Aims/purpose	What were the aims or purpose of the study?<br><br>The authors note that, "A key challenge within the rise of data has been the unequal distribution of data literacy". The aim of the paper is to "present novel approaches to learning technologies and activities, focused on novice learners entering the field of data driven storytelling." (abstract, p.1 of PDF np)<br><br>**What was the rationale for using the arts?**<br><br>The authors argue that "…teaching data literacy to computer scientists and statisticians is a different proposition than teaching data literacy to non-technical, adult newcomers such as humanities scholars, journalists, educators, artists and non-profit staff … For these reasons, we assert that teaching information analysis and design to learners without technical backgrounds requires a set of alternate approaches".(p.2 in PDF np)<br><br>These approaches are referred to as "creative data literacy" (p. 1 in PDF np).<br><br>* Increase accessibility<br><br>* Other: reduce inequalities

Rationale for Using the Arts in Data Literacy Context Choices:<br><br>*Increase accessibility*<br><br>*Increase engagement*<br><br>*Develop critical thinking skills*<br><br>*Other*	Increase accessibility<br><br>Increase engagement
Methodology	**What methodological design was utilized for the study?**<br><br>The paper presents a description of novel approaches to creative data literacy.
**Key findings relating to the role of the arts in data literacy**	
**Methods**	**What specific methods (e.g. qualitative, quantitative, mixed methods) were utilized?**<br><br>Qualitative narrative description of learning approaches<br><br>**Which art forms were used?** Data visualization and data storytelling
	**Was data literacy defined and, if so, how?**<br><br>Yes:<br><br>"…define data literacy as "the ability to read, work with, analyze and argue with data as part of a broader process of inquiry into the world" [D'Ignazio and Bhargava 2016]" (p.2 of PDF)
	**Was data literacy measured and, if so, how?**<br><br>No – the paper provides a descriptive narrative of novel learning approaches to creative data literacy
	**Was there an evaluation of the role of the arts in enhancing data literacy and, if so, how?**<br><br>No – the paper provides a descriptive narrative of novel learning approaches to creative data literacy – however, the value of creative approaches to data literacy is proposed:<br><br>"Reflecting on the background, contexts, and settings of the learners that we work with drives us to offer more

	creative and engaging introductions related to data storytelling and data visualization. This work can draw from long histories of more creative and empowering approaches to learning." (p. 2 in PDF)
**Study Population**	**What population groups are being studied in the literature?**<br>U/g and graduate students in US colleges:<br>"undergraduate and graduate courses at MIT and Emerson College," no page numbers (p.4 of PDF)
	**What was the sample size?**<br>Not given
**Research Setting**	Was it a community-based setting?<br>Was it an educational setting? Yes<br>Was it a healthcare setting?
**Findings/results**	**How have the arts have been used to enhance data literacy?**<br>Data visualization and data storytelling used to enhance engagement:<br>For example, on p.5, the use of word counting technologies are used to analyse song lyrics.<br>On p.6, an approach to data storytelling is described:<br>"Learners are asked to bring their current story in the form of a template that says says "the data say _______, we want to tell that story because _______". We then give each group a large piece of paper and a pair of scissors. We then lead them through a classic technique of folding that paper, with one small cut, into a small book with 3 two-page spreads, a front cover, and a back cover. Once the paper is in book form, each group is instructed to write "once upon a time…" on the cover and "the end" on the back. The rest of the pages are available for them to sketch out their story in a form similar to a children's storybook, using large graphics and story text. We offer crayons or thick markers as the implements to write with, to both suggest the playful approach and keep the visuals they choose to include at low fidelity." (p. 6)

Was the evaluation of the role of the arts positive, negative or mixed?	**Was the evaluation of the role of the arts positive, negative or mixed?**<br><br>No evaluation is given but the authors argue for the importance of creative approaches to data literacy:<br><br>“That said, we consider this work preliminary. Much work remains to be done in clarifying and standardizing the definition of data literacy, especially in relation to a shifting field of technological developments and visual communication practices. What should data literacy look like for non-technical learners, who will not go on to be data scientists, but who will need to communicate with data in their professional lives? Finally, there is room for inquiry into how to best measure and evaluate the learning that is taking place for non-technical adult learners. That said, this paper argues that the best way forward is through engaging learners where they are with hands-on creative activities that build their capacity” (p 7-8 in PDF)<br><br>*[End of D'Ignazio and Bhargava 2018]*

Forster et al. 2018

**Charting Elements**	**Associated Questions**
**Publication Details**	Forster, M., Bestelmeyer, S., Baez-Rodriguez, N., Berkowitz, A., Caplan, B., Esposito, R., Grace, E., and McGee, S. (2018) ‘Data Jams’, *Science Teacher*, Vol. 86 Issue 2, 48-53. https://doi.org/10.2505/4/tst18_086_02_48
**Study ID**	Forster et al. 2018
**Title**	‘Data Jams’, *Science Teacher*, Vol. 86 Issue 2, 48-53.
Author(s)	**Who are the authors of the publication?**<br><br>Forster, M., Bestelmeyer, S., Baez-Rodriguez, N., Berkowitz, A., Caplan, B., Esposito, R., Grace, E., and McGee, S.
Year of Publication	When was the paper/study published? 2018
Origin/Country of origin	**Where was the study carried out?**<br><br>The paper reports on Data Jams carries out across the US, noting that:<br><br>“Most Data Jams have been sponsored by long-term ecological research (LTER) sites such as the Baltimore

	Ecosystem Study, Jornada Basin, and Luquillo (Puerto Rico). These sites belong to a network funded by the National Science Foundation to study long-term and largescale ecological phenomena. In New York, the Hudson Data Jam is sponsored by the nonprofit Cary Institute of Ecosystem Studies" (p.49).
Publication Type	**Is the publication a journal article, book or book chapter, review, opinion paper, grey literature, other?**<br><br>Journal article
**General Overview of Study**	
Aims/purpose	**What were the aims or purpose of the study?**<br><br>The article introduces a model called "Data Jams":<br><br>" This article introduces the Data Jam model and how teachers can use it in classroom or after-school settings, supported by vignettes of student projects and feedback from teachers and students" (p. 49).<br><br>The project was motivated by a desire to make engagement with data more accessible and engaging for students:<br><br>" Most have limited experience with authentic scientific data sets, and find them complex and intimidating (Ben-Zvi and Garfield, 2004).<br><br>**What was the rationale for using the arts?**<br><br>The authors were inspired by the use of the arts to support engagement with and communication of data and were particularly interested in how it could be used to support communication around ecological data:<br><br>"Interest in creative and artistic science communication tools has increased, as evidenced by the proliferation of projects such as the SciShow YouTube video series and the Dance Your Ph.D. contest from the American Association for the Advancement of Science (see "On the web.") Inspired by this movement, fourscience education organizations have developed the Data Jam model to engage high school students in learning about ecological research while igniting their creativity" (p. 49).
Rationale for Using the Arts in Data Literacy Context Choices:<br><br>*Increase accessibility*<br><br>*Increase accessibility: increased confidence*	Evidence of all of these:<br><br>" encouraging collaboration, supporting interdisciplinary thinking, enhancing data analysis and communication skills, strengthening connections to the local environment, and fostering a deeper understanding of

Increase engagement: communication skills<br><br>*Develop critical thinking skills:* interdisciplinary thinking, deeper understanding of core scientific concepts<br><br>*Other:* collaboration and teamwork, connection with environment	core scientific concepts. Students in the Cary Institute Data Jam **ranked teamwork as the top skill they gained from the process**" (p.50).
Methodology	**What methodological design was utilized for the study?**<br><br>This paper reported on the implementation of Data Jams in schools but did not engage in an analysis or evaluation of these projects. It provided vignettes to "illustrate how students successfully and creatively interpreted their data" (p.52) as well as some direct quotes from teachers.
**Key findings relating to the role of the arts in data literacy**	
**Methods**	**What specific methods (e.g. qualitative, quantitative, mixed methods) were utilized?**<br><br>The paper used vignettes and teacher quotes to present its findings.<br><br>**Which art forms were used?**<br><br>The paper emphasized the importance of students choosing their own art form to communicate the data:<br><br>"Giving students the choice to work on what they want is important" (p. 52).<br><br>"We encourage students to apply their interests and hobbies to this component (e.g., dance, painting, game design, skitwriting, making three-dimensional models)" (p.49).<br><br>"Thousands of students around the country have participated in activities using the Data Jam model, creating poetry, songs, videos, or sculpture to improve their data literacy" (p.49).<br><br>Visual arts, music, theatre, literature, film and dance
	**Was data literacy defined and, if so, how?**<br><br>No definition was provided. The paper noted that: "Data is the lens through which we increasingly view our world" (p.49).

	**Was data literacy measured and, if so, how?**<br><br>Data literacy was not measured. However, it was noted that feedback on the data jams was largely positive:<br><br>"Feedback on Data Jams has been positive, and many teachers have participated for multiple years. In surveys from the Cary Institute, both teachers and students reported that students gained content knowledge, showed increased ability to discuss their topic, improved their data analysis skills, and increased their confidence with analyzing data" (p.50).
	**Was there an evaluation of the role of the arts in enhancing data literacy and, if so, how?**<br><br>Feedback on the role of the arts was largely positive:<br><br>"Students enjoy the creative component the most, and one student wrote, "I think that it is cool that we are making a creative piece about data that is supposed to be serious"" (p.50).<br><br>"Students enjoy the creative aspects of Data Jam, which motivates them through the challenges of data analyses. One 2017 New York Data Jam teacher wrote, "Students who didn't previously like science really enjoyed working on the creative project ... it provided a window into understanding the science"" (p.51).<br><br>However, it was noted that it is important that the creative element must accurately represent the data:<br><br>"While students should have freedom to express their findings using any format they choose, it is important to emphasize that the creative project should accurately represent the data in an appealing way" (p.51).<br><br>"Planning and producing the creative component of the Data Jam is motivating but also challenging. Students get excited about this part of the project, but can stray from the content of the data. For instance, in 2014, a team of Baltimore students chose a data set on local stream chloride concentrations. Their data analysis was excellent, but their creative project championed trash prevention in Baltimore Harbor" (p.53).
**Study Population**	**What population groups are being studied in the literature?**<br><br>High school students in the USA
	**What was the sample size?**

	No sample sizes provided. Noted that students tend to work in groups of three when working on a data project.
**Research Setting**	Was it a community-based setting? * some community based elements in the sharing of the creative projects in eg. "This can take many forms, from a classroom gallery walk to a public exhibition" (p.51).<br><br>Was it an educational setting? Yes<br><br>Was it a healthcare setting?
**Findings/results**	**How have the arts have been used to enhance data literacy?**<br><br>The arts support creative engagement and communication of data-based, ecological information, enhancing pride in collaborative work, creative development and fun:<br><br>"*Teachers report* that the Data Jam can be very challenging, albeit worthwhile, for their students. One Baltimore teacher exclaimed that the most important thing she got from the Data Jam was "seeing the students take pride in something they worked hard on," while another was thrilled to watch "some hidden creativity emerge" in her students. Tips for addressing the major challenges of Data Jam are summarized in Figure 5. And finally, as one 2015 Data Jam teacher advised, "have fun with it!" (p.53).
Was the evaluation of the role of the arts positive, negative or mixed?	**Was the evaluation of the role of the arts positive, negative or mixed?**<br><br>Very positive but included a caution to make sure the creative element accurately communicates the data.<br><br>*[End of Forster et al. 2018]*

Fotopoulou 2020

**Charting Elements**	**Associated Questions**
**Publication Details**	Fotopoulou, A. (2020). Conceptualising critical data literacies for civil society organisations: agency, care, and social responsibility. *Information, Communication & Society*, *24*(11), 1640–1657. https://doi.org/10.1080/1369118X.2020.1716041

**Study ID**	Fotopoulou 2020
**Title**	Conceptualising critical data literacies for civil society organisations: agency, care, and social responsibility.
Author(s)	**Who are the authors of the publication?**<br><br>Fotopoulou, A.
Year of Publication	**When was the paper/study published?**<br><br>2020
Origin/Country of origin	**Where was the study carried out?**<br><br>UK
Publication Type	**Is the publication a journal article, book or book chapter, review, opinion paper, grey literature, other?**<br><br>Journal article
**General Overview of Study**	
Aims/purpose	**What were the aims or purpose of the study?**<br><br>"The primary purpose of the article is to move forward the debate around how to conceptualise data literacy – and to question how far the concept is useful in the first place" (p. 1642). The author draws on empirical work to do this, i.e. a training programme with the aim of enhancing critical data literacy.<br><br>Overall rationale:<br>"…it is imperative to develop frameworks and training schemes that enable civil society actors and publics more generally to use open data for advocacy, to make data more relevant and useful to stakeholders, and to support their engagement in policy debates around datafication" (p. 1642).<br><br>**What was the rationale for using the arts?**<br><br>The author argue that there is "a pressing need for data literacies that move beyond a focus on enhancing quantitative analysis and technical skills"

	(p. 1641) by adopting a creative approach. Storytelling is the creative approach used here, in the training workshops discussed:<br><br>"Following the mapping of existing data literacy projects,2 the development of the critical data literacy instrument comprised two training workshops that used a combination of tool-based capacity-building and critical skills through **storytelling**, visualisation and analytics. It aimed to address the knowledge needs and skills of these actors, in order to make data accessible and to equip them with the resources necessary for addressing the critical and ethical questions that relate to datafication" (p. 1642).<br><br>*Increase accessibility<br><br>*Increase engagement<br><br>*Develop critical thinking skills<br><br>*Enhance data storytelling abilities<br><br>*Enhance critical data literacy
Rationale for Using the Arts in Data Literacy Context Choices:<br><br>*Increase accessibility*<br><br>*Increase engagement*<br><br>*Develop critical thinking skills*<br><br>*Other*	
Methodology	**What methodological design was utilized for the study?**<br><br>Empirical research drawn from a pilot training intervention:<br><br>"The empirical research entailed the development of a training instrument for critical data literacies, which was piloted in training workshops with seven civil society organisations in the South East of England" (p. 1642).
**Key findings relating to the role of the arts in data literacy**	

**Methods**	**What specific methods (e.g. qualitative, quantitative, mixed methods) were utilized?**<br><br>Does not say but appears to be qualitative:<br><br>“(a)Tool-based and capacity-building work consisting of the development of the data analytics training instrument and workshops with civil society organisations and community groups.<br>(b)Participant observation and a focus group interview with workshop participants.<br>(c)Desk research consisting of mapping existing projects in the field of data literacy” (p. 1654).<br><br>**Which art forms were used?**<br><br>Storytelling
	**Was data literacy defined and, if so, how?**<br><br>Data literacy was not specifically defined. A key argument throughout is that “data literacies should be understood as ‘social literacies’. Literacies was here used to underline that data literacy practices are multiple and intersecting, linking critical media, information literacy and digital literacy as essential components for citizen engagement. Moreover, framing data literacies as contextual, the article showed how data literacies can shape the data practices of civil society organisations within their respective institutional, material and symbolic contexts, and, in turn, should be informed by these contexts” (p. 1654).
	**Was data literacy measured and, if so, how?**<br><br>Data literacy was not specifically measured.
	**Was there an evaluation of the role of the arts in enhancing data literacy and, if so, how?**<br><br>Yes, the role of storytelling was evaluated based on the reflections of the participants engaged in the training programme workshops in tandem with a mapping of other data literacy projects in published literature.
**Study Population**	**What population groups are being studied in the literature?**<br><br>“The study involved eight representatives of seven organisations: an organisation that provides older and young people with support in improving their

	quality of life, such as housing and advocacy; a mental health charity for LGBTQ people; a charity that supports survivors of child sexual abuse, sexual abuse and domestic violence; an organisation that supports young people and families across Sussex, providing accommodation, family work, health, advice, and education services; a music charity specialising in grassroots music and talent development, social change and innovation in education; a foundation supporting people with learning disabilities by finding accommodation, organising activities and providing community support; and a charity that provides suicide prevention training" (p. 1642).<br><br>"Participants were identified from a network of over 2300 organisations and community groups with the aid of Community Works, a platform that connects the charity and voluntary sector" (p. 1654).
	**What was the sample size?**<br><br>"A total of 12 community groups were recruited, and 8 people took part in the Workshops" (p. 1654).
**Research Setting**	Was it a community-based setting? Yes.<br><br>Was it an educational setting?<br><br>Was it a healthcare setting?
**Findings/results**	**How have the arts have been used to enhance data literacy?**<br><br>The participants engaged with data storytelling in workshops designed to enhance critical data literacy. Areas of enhancement included interpretation, communication, and critical reflection on community data stories.
Was the evaluation of the role of the arts positive, negative or mixed?	**Was the evaluation of the role of the arts positive, negative or mixed?**<br><br>Positive. The reflections of the participants illustrate a better understanding of data interpretation and communication, increased awareness of the power of emotional engagement in community stories, and critical awareness in community storytelling in terms of "considerations about the shaping of ethically and socially responsible data practices" (p. 1650).<br><br>"…data stories become the link between the personal and the public, between an individual experience and a larger scale issue that affects a whole community" (p. 1649).

	"Reflecting on the training they received overall during the capacity building workshops, participants valued how data analytics and data visualisation could be used in order to better communicate their organisation's aims and actions. Participants reflected particularly on the hands-on training of the storytelling sessions (where we talked about the process of making creative briefs and developing narratives from data), and the data visualisation sessions (where we used the online infographics application infogr.am to create simple data graphs). Many felt that learning how to do these tasks and use data effectively was significant, because it would allow them to communicate the work that they did, to educate audiences about the mission and priorities of their organisation, and potentially even get a specific campaign off the ground" (p. 1648).<br><br>"Learning how to tell stories with data was understood to be central in making sense of common experiences and connecting people" (p. 1649).<br><br>"Since participants already had the media literacy skills and competences required in order to create campaign material for their organisations through storytelling, the data literacy offered in the sessions added a new layer to their existing skills. Participants valued how learning to turn data into stories could potentially allow them to engage audiences emotionally by highlighting the scale of an issue, and, this way, to make a story more powerful" (p. 1649).<br><br>*End of Fotopoulou 2020*

Galbraith et al. 2024

**Charting Elements**	**Associated Questions**
**Publication Details**	Galbraith, A., Bertling, J.G., Wandell, T., Swartzentruber, R. and Hodge, L., 2024. Data story finding and storytelling: Arts-based data visualization in art and STEM classrooms. *Art Education*, *77*(2), pp.8-16.
**Study ID**	Galbraith et al. 2024
**Title**	Data story finding and storytelling: Arts-based data visualization in art and STEM classrooms
Author(s)	**Who are the authors of the publication?**

	Galbraith, A., Bertling, J.G., Wandell, T., Swartzentruber, R. and Hodge, L.
Year of Publication	**When was the paper/study published?**<br><br>2024
Origin/Country of origin	**Where was the study carried out?**<br><br>USA
Publication Type	**Is the publication a journal article, book or book chapter, review, opinion paper, grey literature, other?**<br><br>Journal article
**General Overview of Study**	
Aims/purpose	**What were the aims or purpose of the study?**<br><br>The article "explores how three science, technology, engineering, arts, and mathematics (STEAM) curricula supported students in interpreting data and constructing data stories as part of the Arts-Based Data Visualization Project" (Galbraith et al. 2024, p. 8).<br><br>**What was the rationale for using the arts?**
Increase accessibility<br><br>*Increase engagement*<br><br>*Develop critical thinking skills*<br><br>*Other*	Increase accessibility<br><br>Increase engagement
Methodology	**What methodological design was utilized for the study?**<br><br>The article describes curriculum design, and implementation of the Arts-Based Data Visualization Project.
**Key findings relating to the role of the arts in data literacy**	

**Methods**	**What specific methods (e.g. qualitative, quantitative, mixed methods) were utilized?**<br><br>Story-finding and storytelling were implemented in all unit lessons with the aim of supporting students to tell a range of data stories using various artistic means. Data visualization is conceptualized as "showing information" (Galbraith et al. 2024, p. 8).<br><br>Data gathering and analysis is not described.<br><br>**Which art forms were used?**<br><br>Visual arts, story-finding and storytelling, data sculpture, media installation; creating data visualizations working with "two and three dimensional formats… using various traditional art materials such as papier-mache, paint, modelling clay, and found objects" (Galbraith et al. 2024, p. 10). One class created a large multimedia installation to represent "mercury levels in local fish populations" (Galbraith et al. 2024, p. 11).
	**Was data literacy defined and, if so, how?**<br><br>Yes. "*Data literacy* describes how students interact with data through analysis, interpretation, evaluation (Shreiner, 2020), and examination of the source(s) and processes(s) used to collect the data (Calabrese Barton et al., 2021; Gould, 2017)"(Galbraith et al. 2024, p. 8).<br><br>Elements of visual literacy, statistical literacy and data-visualization literacy are explored, alongside data literacy, as components of artistic storytelling processes.
	**Was data literacy measured and, if so, how?**<br><br>Data literacy was not measured.
	**Was there an evaluation of the role of the arts in enhancing data literacy and, if so, how?**<br><br>No formal evaluation. Researcher summary of the project.

**Study Population**	**What population groups are being studied in the literature?** *[age profile, gender, ethnicity, social group]*<br><br>Three groups of 8th grade students in two schools in a midsize city in southern USA
	**What was the sample size?**<br><br>STEM class: 16 students<br><br>Two media arts classes: 31 and 32 students.
**Research Setting**	Was it a community-based setting?<br><br>**Was it an educational setting? Yes**<br><br>Was it a healthcare setting?
**Findings/results**	**How have the arts have been used to enhance data literacy?**<br><br>"The Arts-Based Visualization Project was designed to inspire and support students to engage with and make sense of data through artistic production" (Galbraith et al. 2024, p.8).<br><br>Students used multiple strategies to tell stories and communicate meaning from data about social and ecological issues in their area. These included "using settings to communicate meaning, building on personal connections, and considering audience perceptions in their designs – each strategy aimed at humanizing the data, making data trends and patterns accessible and meaningful" (Galbraith et al. 2024, p.11).
	**Was the evaluation of the role of the arts positive, negative or mixed?**<br><br>Mixed. The authors advocate for more time for instruction, more time for students to discuss and create traditional graphs prior to creating artistic data visualizations, and more time for reflection and incorporating feedback into their visualizations. They state the intention to develop a support model for educators to address such issues.<br><br>The authors write positively about how the students merged "data and storytelling elements such as setting, connection, and audience can

	humanize numbers in a way that is compatible with art education" (Galbraith et al. 2024, p. 15).<br><br>"Art education and STEAM curricula can empower students to access these various forms of knowledge, interpret them through their own situated experiences, and tell stories in ways that act on audiences" (Galbraith et al. 2024, p. 15).<br><br>*End of Galbraith et al. 2024*

Giamellaro et al. 2020

**Charting Elements**	**Associated Questions**
**Publication Details**	Giamellaro, M., O'Connell, K. and Knapp, M. (2020) 'Teachers as participant-narrators in authentic data stories', *International Journal of Science Education*, Vol. 42 Issue 3, pp. 406-425. https://doi.org/10.1080/09500693.2020.1714093
**Study ID**	Giamellaro et al. 2020
**Title**	'Teachers as participant-narrators in authentic data stories'
Author(s)	Giamellaro, M., O'Connell, K. and Knapp, M.
Year of Publication	**When was the paper/study published?**<br><br>2020
Origin/Country of origin	**Where was the study carried out?**<br><br>USA
Publication Type	**Is the publication a journal article, book or book chapter, review, opinion paper, grey literature, other?**<br><br>Journal article

**General Overview of Study**	
Aims/purpose	**What were the aims or purpose of the study?**<br><br>The aims of this study is to describe<br><br>"Outcomes from a place-based, teacher-scientist partnership intended to support teacher use of authentic data for science and math" *instruction.* (p.406)<br><br>Specifically, the study aimed to explore the following key questions related to the role of storytelling in this case study:<br><br>"(1) Did teachers develop a sense of story via their participation in the place-based teacher scientist partnership? and if so,<br><br>(2) Did teachers identify as agents in those stories?<br><br>(3) Which story elements did teachers use when discussing their experience?<br><br>(4) How did teachers use story to transfer knowledge and practice from the scientists to the students?" (p.408-409)<br><br>What was the rationale for using the arts?<br><br>The paper focuses specifically on the role of storytelling as a complementary tool in the teaching of scientific data and information:<br><br>"While good stories and good arguments are judged by different criteria, Bruner (1986) suggests they are complementary. We adopted this stance and sought to examine how stories and arguments could complement each other as teachers sought to learn, process, and teach content knowledge they learned from cooperating scientists" *(p.408).*
Rationale for Using the Arts in Data Literacy Context Choices:<br><br>*Increase accessibility*<br><br>*Increase engagement*<br><br>*Develop critical thinking skills*<br><br>*Other*	• Noted that storytelling can make knowledge more accessible and relatable: "one goal of storytelling is to situate important ideas into a narrative structure that is understandable and relatable (p.407)<br><br>• Focus was on the communication of scientific knowledge

Methodology	**What methodological design was utilized for the study?**<br>Case study:<br>“We used an exploratory, phenomenological, qualitative case study approach to analyse teacher experiences with the place-based TSP project” (p.408).<br>“The case was built from teacher interviews, and researcher field notes” (p.409).<br>“Data were collected from: (1) researcher notes, (2) teacher focus groups, and (3) individual teacher interviews” (p.409).
**Key findings relating to the role of the arts in data literacy**	
**Methods**	**What specific methods (e.g. qualitative, quantitative, mixed methods) were utilized?**<br>Qualitative – interviews, focus groups and field notes<br>**Which art forms were used?** Storytelling
	**Was data literacy defined and, if so, how?**<br>No definition provided
	**Was data literacy measured and, if so, how?**<br>No – focus was on the role of storytelling in knowledge transmission and communication
	**Was there an evaluation of the role of the arts in enhancing data literacy and, if so, how?**<br>No formal evaluation but it was noted that storytelling may play a key role in knowledge transfer and communication:<br>“This case study investigated how narrative structure and contextualisation were created and used by teachers to convey meaning from the scientists to students…The results show that teacher narratives were based heavily on the scientists’ narratives as informed by teachers’ own field experiences and authentic data “(Abstract, p.406).
**Study Population**	**What population groups are being studied in the literature?**

	Math, science, and elementary teachers from public schools in the US.
	**What was the sample size?**<br><br>“Thirty-three math, science, and elementary teacher participants from public schools in the Western U.S.” (p.409).<br><br>“ all teachers participated in 45-minute focus group interviews in which they responded to six prompts about their experience with the project, outcomes, barriers, and specifics about interactions with the scientists. Similarly, 13 teachers were interviewed (all were invited, 13 were able to) with a structured protocol of seven questions regarding impact on their knowledge, practices, and students’ outcomes”(p.410-411).
**Research Setting**	Was it a community-based setting? * Teachers participated in three retreats based at scientific research stations.<br><br>Was it an educational setting? * Teachers brought findings from their retreats back to the classroom: “Throughout the subsequent fall semester, the teachers tried their new curricula in their classrooms, shared the data and vicarious experiences with their students, and participated in videoconferences with the scientists to clarify or modify the curricula” (p.409).<br><br>Was it a healthcare setting?
**Findings/results**	**How have the arts have been used to enhance data literacy?**<br><br>Teachers were seen to draw on the stories of partner scientists, supplement these through their own field experiences, and:<br><br>“Using narrative structure, teachers condensed and filtered these experiences into a story focused on extrinsic explanation, shared with students through secondary contextualisation, and robustly supported by students’ field experiences” (p.419).
Was the evaluation of the role of the arts positive, negative or mixed?	**Was the evaluation of the role of the arts positive, negative or mixed?**<br><br>The paper concludes that “there is much potential for using narrative structure to support data literacy” (p.423) but also notes that, “story alone does not seem to effectively carry the intrinsic scientific explanations” (p.423).

	"Teachers used data stories to carry extrinsic science explanations to their students rather than the original authentic data … greater intentionality is needed to either incorporate the phenomena of study into the narrative structure or include another mechanism to bring the phenomena into instruction (p.423).<br><br>*End of Giamellaro et al. 2020*

Kahn & Jiang 2021

**Charting Elements**	**Associated Questions**
**Publication Details**	Kahn, J. and Jiang, S.Y. (2021) 'Learning with large, complex data and visualizations: youth data wrangling in modeling family migration', *Learning Media and Technology* 2021 Vol. 46 Issue 2: 128-143. https://doi.org/10.1080/17439884.2020.1826962
**Study ID**	Kahn & Jiang, 2021
**Title**	Learning with large, complex data and visualizations: youth data wrangling in modeling family migration
Author(s)	**Who are the authors of the publication?**<br><br>Kahn, J. and Jiang, S.Y.
Year of Publication	**When was the paper/study published?**<br><br>2021
Origin/Country of origin	**Where was the study carried out?**<br><br>Doesn't say country explicitly but researchers based in the US: "Middle and high school youth (N = 17; 6 male; 11 female; self-identified as 13 African-American; 3 White; 1 Asian; Mean hours of attendance = 13) created family data storylines to explore reasons for personal family mobility (the family geobiography; Kahn 2020) as well as national and global migration in a free summer workshop at a city public library" (p.130).
Publication Type	**Is the publication a journal article, book or book chapter, review, opinion paper, grey literature, other?**<br><br>Journal article:

	Learning Media and Technology 2021 Vol. 46 Issue 2: 128-143.<br>https://doi.org/10.1080/17439884.2020.1826962
**General Overview of Study**	
Aims/purpose	**What were the aims or purpose of the study?**<br><br>The study set out to address the following questions:<br><br>"(1) What are the patterns of youth's data wrangling trajectories for building models and telling stories about family migration?<br><br>(2) What are the stages that describe youth learning in their data wrangling trajectories for building models and telling stories about family migration?" (p.130).<br><br>"The goal of the project was to understand how youth interact with large, complex, geospatial datasets and data visualization technologies to tell stories about family migration. The main learning objective was for youth to learn to tell stories with these data in a personal context" (p.131).<br><br>**What was the rationale for using the arts?**<br><br>While the arts are not mentioned explicitly, storytelling is mentioned several times as is data visualization (see above quote) and:<br><br>"Then they captured screenshots of models or maps and inserted them into a Microsoft PowerPoint" (p.131).<br><br>The rationale for using arts was to **engage "**students in **meaningful** data exploration through establishing relevance and making **personal connections** to the data" (p. 129)<br><br>Increase accessibility<br><br>Increase engagement<br><br>Develop critical thinking skills
Rationale for Using the Arts in Data Literacy Context Choices: Increase accessibility<br><br>*Increase accessibility*<br><br>*Increase engagement*<br><br>*Develop critical thinking skills*	

Other	
Methodology	**What methodological design was utilized for the study?**<br><br>Described the study as a “a micro-analysis of youth interactions with large complex, socioeconomic datasets and data visualization tools” (p.128).
**Key findings relating to the role of the arts in data literacy**	
**Methods**	**What specific methods (e.g. qualitative, quantitative, mixed methods) were utilized?**<br><br>Primarily qualitative:<br><br>“We video and audio recorded all activities, recorded participants’ work on laptop computers with screen capture software, and collected field notes and their final artifacts. Our analytical approach built on Hmelo-Silver et al.’s (2011) work in using representational tools to visualize and analyze complex temporal, interactional digital activities. We conducted a close-up examination of student talk as well as their multimodal interactions with the computer interfaces (clicks, visible gestures) and artifacts (their evolving and final family data storylines), since data selections **were not always accompanied by verbal explanations** of the aggregate data trends” (p.131-132).<br><br>**Which art forms were used?**<br><br>• data visualization<br>• data stories / story-telling
	**Was data literacy defined and, if so, how?**<br><br>Data literacy was not defined but a definition was provided for “data-wrangling”:<br><br>“We define data wrangling as the set of practices to manage and select meaningful data from a large, complex database in order to communicate insights from data analysis (Jiang and Kahn 2020)” (p. 129).
	**Was data literacy measured and, if so, how?**<br><br>Data literacy was not measured. The goal of the paper was to describe how a group of students engaged in a data project through close-up analysis of youth-computer interactions.<br><br>A model was proposed based on this analysis:

	“The Find, Relate, Challenge, Build model” (p.140)
	**Was there an evaluation of the role of the arts in enhancing data literacy and, if so, how?**<br><br>There was no specific evaluation of the role of the arts but through a micro-analysis of youth-computer interactions with large data-sets, the paper proposes a model to support data story-telling and visualization:<br><br>“Our Find, Relate, Challenge, Build model describes the possible stages of data wrangling activity that youth moved through in order to build models and tell stories about family migration with complex, large datasets and tools.” (p.140)
**Study Population**	**What population groups are being studied in the literature?**<br><br>“Middle and high school youth” (p. 130).
	**What was the sample size?**<br><br>“(N = 17; 6 male; 11 female; self-identified as 13 African-American; 3White; 1 Asian)” (p.130).
**Research Setting**	Was it a community-based setting? Yes<br><br>Was it an educational setting? Yes<br><br>“a free summer workshop at a city public library” (p.130)<br><br>“libraries have become established social and learning community spaces for youth to explore fields of interest.” (p.131)<br><br>Was it a healthcare setting?
**Findings/results**	**How have the arts have been used to enhance data literacy?**<br><br>The project used data visualization and storytelling as part of its approach to engaging with large data-sets
Was the evaluation of the role of the arts positive, negative or mixed?	**Was the evaluation of the role of the arts positive, negative or mixed?**<br><br>There was no specific evaluation of the role of the arts – the study suggested that it provided new insights which warrant further investigation:

	"Our findings provide **new insights** on the nature of how youth interact with large, complex datasets and data visualization tools, which are growing in use in educational settings. Close-up analyses of youth–computer interactions like ours **inform the learning opportunities** and challenges that students might encounter while **telling stories and modeling** with open datasets. However, **more research is needed** to extend and build on our understanding of data wrangling as a key critical data literacy activity (p. 141)"<br><br>*[End of Kahn & Jiang, 2021]*

Kahn & Jiang 2024

**Charting Elements**	**Associated Questions**
**Publication Details**	Kahn, J. and Jiang, S., 2024. Leveraging epistemic data agency with data visualizations to bridge the gap between data trends and personal experiences. *Information and Learning Sciences*, *125*(11/12), pp.1126-1145.
**Study ID**	Kahn & Jiang 2024
**Title**	Leveraging epistemic data agency with data visualizations to bridge the gap between data trends and personal experiences.
Author(s)	**Who are the authors of the publication?**<br><br>Kahn, J. and Jiang, S.
Year of Publication	**When was the paper/study published?**<br><br>2024
Origin/Country of origin	**Where was the study carried out?**<br><br>USA
Publication Type	**Is the publication a journal article, book or book chapter, review, opinion paper, grey literature, other?**<br><br>Journal article
**General Overview of Study**	

Aims/purpose	**What were the aims or purpose of the study?**<br><br>The authors analyzed three student cases from a design study (17 participants) in order to use their findings to point out the learning opportunities and challenges that may help to "support students enacting epistemic data agency" (Kahn & Jiang 2024, p. 1132) in the context of designing future projects for such learning environments. The focus of the paper is on "how learners navigate tensions between personal experiences and data trends" (Kahn & Jiang 2024, p. 1126) which they suggest is an overlooked aspect in designing activities aimed at enhancing data literacy.<br><br>**What was the rationale for using the arts?**<br><br>The authors argue that tension exists between data and student's personal experiences, but they suggest that this leads to learning opportunities and can "open spaces for rich reflection, engagement and learning" (Kahn & Jiang 2024, p. 1131). The authors' aim here is to investigate the gaps that middle and high school students perceive between their family migration stories and data trends as spaces of "epistemic data agency" meaning "students' capacities to shape knowledge-building of family history, data tool use and data reasoning" (Kahn & Jiang 2024, p. 1131).
Increase accessibility<br><br>*Increase engagement*<br><br>*Develop critical thinking skills*<br><br>*Other*	Increase engagement<br><br>Develop critical thinking skills<br><br>Develop and understand data storytelling as a two-way process
Methodology	**What methodological design was utilized for the study?**<br><br>The authors report on a comparative analysis of middle and high school student cases from a design-based study merging family migration histories and large-scale data in a summer workshop. Three cases were selected for analysis.
**Key findings relating to the role of the arts in data literacy**	

**Methods**	**What specific methods (e.g. qualitative, quantitative, mixed methods) were utilized?**<br><br>Summer workshop: “Storytelling with Big Data”. The students interacted with “web-based data visualization platforms (Social Explorer and Gapminder)” (Kahn & Jiang 2024, p. 1131).<br><br>Data gathering: analytic memos consisting of screenshots - and video recordings, primarily documenting challenges in the storytelling process encountered during workshop time.<br><br>Researcher analysis.<br><br>**Which art forms were used?**<br><br>Storytelling [personal stories – real and imagined]
	**Was data literacy defined and, if so, how?**<br><br>Data literacy is not formally defined but its meaning is implied in the context of this study. The authors argue that, as seen in the cases in this paper, “addressing gaps between personal experiences and data trends requires deep inquiry about data and its contexts, which is a key part of data literacy (Gebre, 2022; Wilkerson and Laina, 2018)” (Kahn & Jiang 2024, p. 1141).
	**Was data literacy measured and, if so, how?**<br><br>Data literacy was not measured.
	**Was there an evaluation of the role of the arts in enhancing data literacy and, if so, how?**<br><br>Interaction analysis was used to examine student responses to perceived discrepancies between family experiences and data trends in the students’ models, “drawing on the construct of epistemic data agency” (Kahn & Jiang 2024, p. 1126). The authors specify that interaction analysis extends analyses in the authors’ previous studies by “investigating students’ responses to instructional design” (Kahn & Jiang 2024, p. 1131). They suggest that addressing gaps between personal stories and data trends, in this case telling family migration

	stories, involves deep inquiry which they posit as a key component in data literacy.
**Study Population**	**What population groups are being studied in the literature?**<br>Participants are described as "…diverse middle and high school youth" in a city library in southern USA (Kahn & Jiang 2024, p. 1131).
	**What was the sample size?**<br>17 students
**Research Setting**	**Was it a community-based setting?** Yes –public library summer project with an educational focus.<br>Was it an educational setting?<br>Was it a healthcare setting?
**Findings/results**	**How have the arts have been used to enhance data literacy?**<br>In this study, personal stories were used by the students to challenge gaps that they perceived existed in broader data trends. They incorporated their real stories but also imagined stories i.e. what might have occurred if circumstances or context had been different in their lived experiences and those of other family members. The authors argue that this challenging of gaps in the data trends represents an often overlooked aspect of activities designed to enhance data literacy.
	**Was the evaluation of the role of the arts positive, negative or mixed?**<br>The authors argue that using epistemic data agency as an analytical lens illustrates "a two-way relationship between aggregate data and personal stories" (1137)<br>The authors acknowledge the importance of further validation of their findings in other contexts. They emphasize the need to support students in owning their data stories while also addressing the challenges that emerge when exploring the gaps

	between personal experience and data trends, and developing multiple models to facilitate this. "Strategies for addressing gaps should be explicitly taught, such as examining outliers, assembling multiple models and focusing on the contextual factors underlying the data trends" (1141).<br><br>They also stress the importance of providing professional development training focusing on enhancement of critical data literacies for teachers.<br><br>*End of Kahn and Jiang 2024*

Lee et al. 2021

**Charting Elements**	**Associated Questions**
**Publication Details**	Lee, V.R., Wilkerson, M.H., and Lanouette, K. (2021) 'A Call for a Humanistic Stance Toward K-12 Data Science Education', *Educational Researcher*: 664-672.<br><br>https://doi.org/10.3102/0013189x211048810
**Study ID**	Lee, Wilkerson & Lanouette, 2021
**Title**	'A Call for a Humanistic Stance Toward K-12 Data Science Education'
Author(s)	Who are the authors of the publication?<br><br>Lee, V.R., Wilkerson, M.H., and Lanouette, K.
Year of Publication	When was the paper/study published? 2021
Origin/Country of origin	Where was the study carried out?<br><br>It does not state this explicitly but the authors are based in the U.S.
Publication Type	Is the publication a journal article, book or book chapter, review, opinion paper, grey literature, other?

	Journal article
**General Overview of Study**	
Aims/purpose	**What were the aims or purpose of the study?**<br><br>The aim of this study is to propose a humanistic approach to data literacy through the presentation of two case studies. It presents:<br><br>"two examples how our proposed humanistic stance highlights ways that efforts to make data personally relevant for youth also necessarily implicate cultural and sociopolitical dimensions" (Abstract, p.664).<br><br>What was the rationale for using the arts?<br><br>There is no explicit discussion on a rationale for using the arts. However, it is noted that certain data tools can limit artistic engagement with data:<br><br>"More broadly, using digital data analysis tools provides powerful statistics and visualizations, but can limit students' opportunities to explore more artistic visualization methods that emphasize trajectories of experience, outliers, and storytelling (e.g., Lupi & Posavec, 2016)" (p.666).<br><br>There are a number of references to the value of storytelling and data visualization in supporting data literacy:<br><br>"This emphasis has also shaped the ways in which students were to engage with and personalize particular data sets (e.g., through filtering, grouping, or adding records). CODAP does enable students to add multimedia such as images and text to their data "document," supporting the storytelling aspect of the work (Wilkerson et al., 2021)" (p.669).<br><br>"our goal in this article is to articulate ways in which educators and researchers can deliberately center these human dimensions of student **engagement** with data—what we call a humanistic stance toward datascience education" (p.665)<br><br>"develop learning experiences that encourage **thoughtful** and **critical participation** in practices of data creation, interpretation, analysis, argumentation, and critique" (p.670).<br><br>Increase engagement

	Develop critical thinking skills
Rationale for Using the Arts in Data Literacy Context Choices: Increase accessibility<br>*Increase engagement*<br>*Develop critical thinking skills*<br>*Other*	Notes importance of engagement for critical learning
Methodology	**What methodological design was utilized for the study?**<br>The development of a theoretical framework and two case study examples
**Key findings relating to the role of the arts in data literacy**	
**Methods**	**What specific methods (e.g. qualitative, quantitative, mixed methods) were utilized?**<br>Qualitative – literature review and case study description<br>Which art forms were used?<br>Storytelling, data visualisation
	**Was data literacy defined and, if so, how?**<br>No definition is provided for data literacy. However there is a definition for "authorship practices" as follows:<br>"By authorship practices, we mean students' direct involvement in the design and construction (or reconstruction) of a data set as a text—including but not limited to its structure, decisions about what to include in the data set, methods of quantification or categorizing, sampling, generation and recording of data, and data cleaning" (p.667).
	**Was data literacy measured and, if so, how?**<br>No – the paper was more descriptive than evaluative
	**Was there an evaluation of the role of the arts in enhancing data literacy and, if so, how?**<br>No- the paper was more descriptive than evaluative

**Study Population**	**What population groups are being studied in the literature?**<br><br>Two population groups were mentioned in the case studies – one was and one was middle school<br><br>Case study one: "Over many years, Lee has developed a program of research and development to engage elementary school students in data analysis through their collection of physical activity data during recess using commercially manufactured wearable devices (Lee, 2019)" (p. 667).<br><br>Case study two:<br><br>"Wilkerson, Lanouette, and colleagues have been studying middle school youths' use of publicly sourced scientific data sets to explore and share stories about how key issues such as nutrition and climate change impact themselves and their communities (Lopez et al., 2021)" (p.669).
	**What was the sample size?**<br><br>Not mentioned
**Research Setting**	Was it a community-based setting?<br><br>Was it an educational setting? Yes<br><br>Was it a healthcare setting?
**Findings/results**	**How have the arts have been used to enhance data literacy?**<br><br>The arts support a more humanistic approach to data literacy, combining personal, cultural and sociopolitical layers of data engagement, as well as more artistic interaction with data:<br><br>"to explore more artistic visualization methods that emphasize trajectories of experience, outliers, and storytelling (e.g., Lupi & Posavec,2016)" (p.666).
Was the evaluation of the role of the arts positive, negative or mixed?	**Was the evaluation of the role of the arts positive, negative or mixed?**<br><br>No explicit evaluation but data storytelling and visualization seen to have the potential to enhance a humanistic approach to data literacy.<br><br>*[End of Lee et al. 2021]*

Lesser et al. 2019

**Charting Elements**	**Associated Questions**
**Publication Details**	Lesser, L.M., Pearl, D.K., Weber lll, J.J., Dousa, D.M., Carey, R.P. and Haddad, S.A. (2019) 'Developing Interactive Educational Songs for Introductory Statistics', *Journal of Statistics Education*, 27:3, 238-252. https://doi.org/10.1080/10691898.2019.1677533
**Study ID**	Lesser et al. 2019
**Title**	"Developing Interactive Educational Songs for Introductory Statistics"
Author(s)	**Who are the authors of the publication?**<br><br>Lesser, L.M., Pearl, D.K., Weber lll, J.J., Dousa, D.M., Carey, R.P. and Haddad, S.A. (2019)
Year of Publication	When was the paper/study published? 2019
Origin/Country of origin	**Where was the study carried out?**<br><br>USA<br><br>Southeastern and midwestern regions of the US
Publication Type	**Is the publication a journal article, book or book chapter, review, opinion paper, grey literature, other?**<br><br>Journal article<br><br>*Journal of Statistics Education*, 27:3, 238-252. https://doi.org/10.1080/10691898.2019.1677533
**General Overview of Study**	
Aims/purpose	**What were the aims or purpose of the study?**<br><br>"The main goal of our project is to develop interactive song resources that require little instructor time or expertise, but that reduce statistics anxiety (Chew and

	Dillon 2014) and have high impact on developing statistical literacy and reasoning (delMas2002)" (p.238).<br><br>"A key purpose of this article is to illustrate the process of developing this distinctive intervention of interactive songs for teaching statistics" (p.238).<br><br>**What was the rationale for using the arts?**<br><br>"the use of song in higher education spans a wide variety of disciplines within and beyond STEM and its possible benefits include **reduced stress or anxiety, improved recall**, and **increased motivation or engagement**. A well-written jingle or song with rhymed lyrics can help content make **a more memorable impression** on students than that same content delivered in prose form (p.238).<br><br>*Increase accessibility<br><br>*Increase engagement<br><br>*Reduce stress or anxiety
Rationale for Using the Arts in Data Literacy Context Choices: Increase accessibility<br><br>*Increase accessibility*<br><br>*Increase engagement*<br><br>*Develop critical thinking skills*<br><br>*Other*	
Methodology	**What methodological design was utilized for the study?**<br><br>Description of educational tool development and two pilot studies using interactive educational songs
**Key findings relating to the role of the arts in data literacy**	
**Methods**	**What specific methods (e.g. qualitative, quantitative, mixed methods) were utilized?**<br><br>Mixed methods (e.g. survey used both a Likert scale and open -ended questions.<br><br>"Data were also collected on student actions with the interface (e.g., clicking on a button or entering a response) and on their responses to a survey. These were recorded in a log file built using xAPI statements (https://xapi.com) implemented using LearningLocker software by ht2labs and analyzed to evaluate student responses. Data rows in the log file contained session IDs

	along with each action the user made together with its time of occurrence" (p.244).<br><br>**Which art forms were used?**<br><br>Music: Songwriting and singing
	**Was data literacy defined and, if so, how?**<br><br>No
	**Was data literacy measured and, if so, how?**<br><br>Two pilot studies evaluated the development of interactive educational songs for introductory statistics.<br><br>"Students self-reported the tool was helpful in **reducing anxiety**, **increasing engagement** with the material, being r**elevant** to their learning, and having a **user-friendly** interface" (p.244).
	**Was there an evaluation of the role of the arts in enhancing data literacy and, if so, how?**<br><br>The response was mostly positive with some suggestions for improvement:<br><br>"The Likert scale data from the two institutions were reinforced by the positive student comments at both institutions that were also collected as part of the survey. The most common comments by far were some versions of "helpful in learning" and "easy to use interface," while the two most common suggestions were to "make songs more fun (e.g., catchier beat)" and "improve sound quality" (e.g., the synthetic voice)" (p. 247).
**Study Population**	**What population groups are being studied in the literature?**<br><br>College/university students
	**What was the sample size?**<br><br>Two pilot studies were conducted:<br><br>One at a majority Black two-year college in the Southeastern region of the US consisted of 13 students<br><br>A second pilot study was conducted at a research university in the midwestern region of the US and consisted of 77 students.

**Research Setting**	Was it a community-based setting?<br><br>Was it an educational setting? Yes<br><br>Was it a healthcare setting?
**Findings/results**	**How have the arts have been used to enhance data literacy?**<br><br>Interactive educational songs were developed to support students in learning introductory statistics
Was the evaluation of the role of the arts positive, negative or mixed?	**Was the evaluation of the role of the arts positive, negative or mixed?**<br><br>The evaluation was largely positive, though a number of ways in which the project good be improved going forward were also described.<br><br>“students found the innovation to be a good tool to help their learning, reduce their anxiety about statistics, have an easy to follow interface, and use high quality songs” (Abstract, p.238).<br><br>Suggestions for improvement include:<br><br>“faster tempos (which is tricky with synthetic voice inserts),<br><br>• more contemporary genres,<br><br>• more varied singers (e.g., including a male vocalist on some songs),<br><br>• shorter length, or<br><br>• familiar melodies (e.g., a parody) to facilitate learning or recalling the song.” (P.247)<br><br>*[End of Lesser et al. 2019]*

Lesser 2025

**Charting Elements**	**Associated Questions**
**Publication Details**	Lesser, L.M., 2025. Poetry projects and activities help make students well-versed in statistics. *Teaching Statistics*. (DOI: 10.111/test.12400).
**Study ID**	Lesser 2025

**Title**	Poetry projects and activities help make students well-versed in statistics
Author(s)	**Who are the authors of the publication?**<br><br>Lesser, L.M. (Lawrence M).
Year of Publication	**When was the paper/study published?**<br><br>2025
Origin/Country of origin	**Where was the study carried out?**<br><br>USA
Publication Type	**Is the publication a journal article, book or book chapter, review, opinion paper, grey literature, other?**<br><br>Journal article
**General Overview of Study**	
Aims/purpose	**What were the aims or purpose of the study?**<br><br>The author discusses the potential benefits for students of using poetry projects and activities in statistics courses and offers examples and precedents that can be utilized as a foundation for teaching statistics. He states that if using poetry in statistics classes, no prior experience is required to use the materials presented in the paper.<br><br>**What was the rationale for using the arts?**<br><br>The author argues that poetry is a means for educators to “engage and connect with statistics students” (Lesser 2025, p. 139).<br><br>He suggests that the idea that numbers don’t speak for themselves can be applied to poetry. “A rich data set or poem can have nuance or complexity that may not be immediately fully appreciated” (Lesser 2025, p. 140). He suggests also that poetry can be used to humanize statistics “where the ideas or language of statistics explores an event in one’s personal life” (Lesser 2025, p. 146). The author cites

	Maspul (2024) to point to the many ways that poetry might be used to “empower students through a poetry-rich classroom: digital poetry platforms, poetry podcasts, community poetry events, multimodal poetry projects (incorporating melody or film), and programs partnering students with local writers” (Lesser 2025, p. 146).
Increase accessibility<br>*Increase engagement*<br>*Develop critical thinking skills*<br>*Other*	Increase engagement<br>Develop critical thinking skills<br>Humanize the data
Methodology	**What methodological design was utilized for the study?**<br>The author shares and discusses “a project and activities used in workshops or a college statistical literacy course” (Lesser 2025, p. 139).
**Key findings relating to the role of the arts in data literacy**	
**Methods**	**What specific methods (e.g. qualitative, quantitative, mixed methods) were utilized?**<br>The paper sets out workshop and activity suggestions for teaching statistics.<br>**Which art forms were used?**<br>Poetry
	**Was data literacy defined and, if so, how?**<br>Data literacy was not defined. Statistical literacy is the focus. No definition is provided.
	**Was data literacy measured and, if so, how?**<br>Data literacy was not measured.
	**Was there an evaluation of the role of the arts in enhancing data literacy and, if so, how?**<br>No formal evaluation. The paper provides a reflection on years of integrating poetry in the

	author’s statistical literacy course and shares insights and templates with the aim of supporting statistical literacy educators.
**Study Population**	**What population groups are being studied in the literature?**<br><br>College students
	**What was the sample size?**<br><br>Not reported.
**Research Setting**	Was it a community-based setting?<br><br>**Was it an educational setting? Yes**<br><br>Was it a healthcare setting?
**Findings/results**	**How have the arts have been used to enhance data literacy?**<br><br>In this study, poetry has been used over a number of years as a means to enhance college students’ statistical literacy. The author shares resources and ideas based on this teaching experience.
Was the evaluation of the role of the arts positive, negative or mixed?	**Was the evaluation of the role of the arts positive, negative or mixed?**<br><br>Evaluation is not overtly discussed. Throughout the paper, the author reflects on poetry as one of many means that can be used in teaching statistics in workshops or classes, based on experience. The author suggests that poetry “can support metacognition as students use the poem to reflect upon their learning and any struggles or misconceptions they face” (Lesser 2025, p. 146).<br><br>Overall, the reflections on the integration of poetry in STEM contexts is positive.<br><br>*End of Lesser 2025*

Li et al. 2023

Charting Elements	Associated Questions
**Publication Details**	Li, Y., Wang, Y., Lee, Y., Chen, H., Petri, A.N., and Cha, T. (2023) 'Teaching Data Science through Storytelling: Improving Undergraduate Data Literacy', *Thinking Skills and Creativity* 2023 Vol. 48, 1-15. https://doi.org/10.1016/j.tsc.2023.101311
**Study ID**	Li et al. 2023
**Title**	'Teaching Data Science through Storytelling: Improving Undergraduate Data Literacy',
Author(s)	**Who are the authors of the publication?**<br>Li, Y., Wang, Y., Lee, Y., Chen, H., Petri, A.N., and Cha, T.
Year of Publication	**When was the paper/study published?**<br>2023
Origin/Country of origin	**Where was the study carried out?**<br>In the USA:<br>" two of the universities were in the Midwest, one in the Northeast, and one in the Southeast of the United States" (p.6)
Publication Type	**Is the publication a journal article, book or book chapter, review, opinion paper, grey literature, other?**<br>Journal article
**General Overview of Study**	
Aims/purpose	**What were the aims or purpose of the study?**<br>The study:<br>"proposes and evaluates the OCEL.AI (Open Collaborative Experiential Learning. AI) paradigm that aims at broadening participation in data science education and enhancing undergraduate students' data literacy " (Abstract, p.1).<br>**What was the rationale for using the arts?**<br>To address challenges in data literacy, the authors suggest that innovative, transdisciplinary approaches

	are needed and propose storytelling as one such approach:<br><br>"…data science education must bridge the gaps in data literacy among the population since data have become a currency of power (D'lgnazio, 2017) in the 21st century. Data science educators need to broaden the participation and reach the widest learners, including undergraduate or graduate students in the STEM fields as well as the non-science fields such as liberal arts, social sciences, and humanity programs (Betz et al., 2020; Cardenas-Navia & Fitzgerald, 2015; Kross et al., 2020). The challenge, however, is to create a transdisciplinary language to communicate with users and learners of diverse backgrounds and perspectives (Flensburg & Lomborg, 2021). The solution presented in this study is the Open Collaborative Experiential Learning (OCEL.AI) and its **"Tell Stories" _approach."** (pp.1-2)<br><br>"We propose that "Tell Stories" can be used for presenting creative ideas (Yang & Wu, 2012) as well as **building strong critical thinking** and problem-solving & abilities in data science education." (p.13)<br><br>*Develop critical thinking skills
Rationale for Using the Arts in Data Literacy Context Choices: Increase accessibility<br><br>*Increase accessibility*<br><br>*Increase engagement*<br><br>*Develop critical thinking skills*<br><br>*Other*	
Methodology	**What methodological design was utilized for the study?**<br><br>"The study consisted of two parts: classroom observations and a field experiment" (p.6).
**Key findings relating to the role of the arts in data literacy**	
**Methods**	**What specific methods (e.g. qualitative, quantitative, mixed methods) were utilized?**<br><br>Mixed method – utilized questionnaire, classroom observation<br><br>**Which art forms were used?**<br><br>Literature/storytelling

	**Was data literacy defined and, if so, how?**<br><br>It was "described" as:<br><br>"Data literacy is a set of important student learning outcomes for data science education. It broadly describes the ability to use data as part of everyday thinking and reasoning for solving real-world problems (Wolff et al., 2016)" (p.2).
	**Was data literacy measured and, if so, how?**<br><br>Yes, using mixed methods including a field experiment, classroom evaluation and a questionnaire (more qualitative data collected but not included in this paper):<br><br>"Qualitative data from interviews and focus groups have already been collected to provide further insights into how students learn data science through OCEL.AI and how they perceive the value of storytelling in data science. Due to the page limit, those findings will be presented in another paper" (p.13).
	**Was there an evaluation of the role of the arts in enhancing data literacy and, if so, how?**<br><br>Yes – the primary purpose of the paper was to propose and evaluate the OCEL.AI (Open Collaborative Experiential Learning. AI) paradigm which uses storytelling.
**Study Population**	**What population groups are being studied in the literature?**<br><br>Undergraduate students in US colleges. Population details collected included gender, ethnicity and STEM/nonSTEM majors.
	**What was the sample size?**<br><br>221 undergraduate students<br><br>" A total of 221 undergraduates at four universities participated in the study in Fall 2020 and Spring 2021" (p.6)
**Research Setting**	Was it a community-based setting?<br><br>Was it an educational setting? Yes<br><br>Was it a healthcare setting?

**Findings/results**	**How have the arts have been used to enhance data literacy?**<br><br>"The analysis compared the means of the control group and the treatment group in terms of students' learning outcomes in data literacy: data science competence, self-efficacy, career motivation, appreciation, life-long will-ingness to learn, **the importance of storytelling, and relevance of storytelling**" (p.8).<br><br>Classroom observation noted three shifts in approach:<br><br>"A major shift for students involved understanding data **not as abstract numbers but as contextualized resources that have real-world meanings and implications**. For instance, a team of journalism students analyzed the descriptive statistics of insurance status and COVID-19 cases in New York State and uncovered that the underserved communities in need of medical help during the pandemic were often those without health insurance. Those uninsured people are more likely to be non-Caucasian, female, without a higher level of education, and jobless" (P.10).<br><br>"The second major shift for students, especially those in the journalism course, involved transforming from a problem-centric storytelling mindset to a solution-oriented engagement. For instance, the journalism students proposed to resolve the health insurance gap by helping uninsured people find alternative and affordable insurance resources in New York" (p.10).<br><br>"The third major shift for both STEM and non-STEM students involved in interdisciplinary collaboration" (p.10).
Was the evaluation of the role of the arts positive, negative or mixed?	**Was the evaluation of the role of the arts positive, negative or mixed?**<br><br>Largely positive:<br><br>"The results showed that the effect of OCEL.AI exposure was significant" (p.8)<br><br>"First, we assessed the effectiveness of the storytelling approach in aiding students' data literacy learning outcomes. Students in the treatment group reported significantly higher average scores on their competence in data science skills and motivation to pursue a career in data science than students in the control group" (p.9).<br><br>*[End of Li et al. 2023]*

McDowell & Turk 2024

Charting Elements	Associated Questions
**Publication Details**	McDowell, K. and Turk, M.J., 2024. Teaching data storytelling as data literacy. *Information and Learning Sciences*, *125*(5/6), pp.321-345.
**Study ID**	McDowell & Turk 2024
**Title**	Teaching data storytelling as data literacy
Author(s)	**Who are the authors of the publication?**<br><br>McDowell, K. and Turk, M.J.,
Year of Publication	**When was the paper/study published?**<br><br>2024
Origin/Country of origin	**Where was the study carried out?**<br><br>USA
Publication Type	**Is the publication a journal article, book or book chapter, review, opinion paper, grey literature, other?**<br><br>Journal article
**General Overview of Study**	
Aims/purpose	**What were the aims or purpose of the study?**<br><br>The purpose of the study is to explore two research questions in the context of data storytelling classes: 1. What themes characterized students' iterative development of data story topics?<br><br>2. Looking back at six years of iterative feedback, what categories of data literacy pedagogy did instructors engage for these themes? (McDowell & Turk 2024, p. 321).<br><br>**What was the rationale for using the arts?**<br><br>The authors argue that data storytelling is a powerful way for students to "stand up and speak

	out about what matters to them with evidence" (McDowell & Turk 2024, p. 339).<br><br>The authors note the long tradition of storytelling in information science but argue that while "[s]tory is a fundamental information form", it has been "overlooked" (McDowell & Turk 2024, p. 325) in this context, often neglecting the importance of agency, narrative, lived experience and audience interpretation. They define storytelling as "telling a story within the dynamic triangle of the story, the teller and the audience" (McDowell & Turk 2024, p. 325).<br><br>"Data storytelling teaches data literacy with agency, placing interpretive power where it belongs: in the hands of storytelling citizens and future information professionals who will use data to advocate for greater justice and inclusion" (McDowell & Turk 2024, p. 326).<br><br>The authors argue that personal and creative approaches like data storytelling are an important part of data literacy pedagogies, and "[d]ata literacy instruction must consider data about current events and contexts that impact students' emotions and everyday lives, not only at the level of personal data risk" (McDowell & Turk 2024, p. 337).
Increase accessibility<br><br>*Increase engagement*<br><br>*Develop critical thinking skills*<br><br>*Other*	Increase engagement<br><br>Develop critical thinking skills<br><br>Expand data storytelling pedagogy
Methodology	**What methodological design was utilized for the study?**<br><br>A case study of two data storytelling courses with a focus on critical data literacy (McDowell and Turk 2024, p. 322)
**Key findings relating to the role of the arts in data literacy**	

**Methods**	**What specific methods (e.g. qualitative, quantitative, mixed methods) were utilized?**<br><br>The researchers analyzed data gathered from 8 semesters of teaching and development on the two data storytelling courses. This included "133 project topics and 73 completed rubrics with instructor feedback" (McDowell & Turk 2024, p. 331).<br><br>**Which art forms were used?**<br><br>Storytelling: categorized as formal, informal and folk.
	**Was data literacy defined and, if so, how?**<br><br>Yes. Drawing on Getz & Brodsky, 2022, data literacy is defined as "…a subset of information literacy that teaches students to access, interpret, critically assess and "ethically use data" (McDowell & Turk 2024, p. 324). They refer to Risdale et al. (2015), Gummer and Mandinach, (2015) and Bhargava et al. 2015, saying that the study "draws on all of these definitions of data literacy as a fundamental and evolving 21st century information skill (McDowell & Turk 2024, p. 324). They also suggest that "data storytelling is more than data literacy; it is a kind of data fluency" (McDowell & Turk 2024, p. 325).
	**Was data literacy measured and, if so, how?**<br><br>Data literacy was not measured.
	**Was there an evaluation of the role of the arts in enhancing data literacy and, if so, how?**<br><br>The researchers use thematic analysis to address the research questions around the students' development of data story topics and the categories of data literacy pedagogy that the instructors engaged in for those themes. They emphasize that the analysis uses social constructionism as "it understands reality to be constructed by human social interaction" (McDowell & Turk 2024, p. 331). They also note that the analysis is 'contextualist' because the "researcher's values were part of shaping the

	knowledge that became the final data story topics" (McDowell & Turk 2024, p. 331).
**Study Population**	**What population groups are being studied in the literature?**<br><br>College students and their instructors.
	**What was the sample size?**<br><br>Not reported.
**Research Setting**	Was it a community-based setting?<br><br>**Was it an educational setting? Yes**<br><br>Was it a healthcare setting?
**Findings/results**	**How have the arts have been used to enhance data literacy?**<br><br>The focus throughout is on analyzing data from two data literacy courses in order to formulate key themes and draw conclusions regarding data storytelling in the context of data literacy pedagogy, and to make recommendations for future data storytelling education based on the findings.
	**Was the evaluation of the role of the arts positive, negative or mixed?**<br><br>Positive.<br><br>"Based on the instructor's reflections, drawing from three categories of data literacy pedagogy (formal, personal and folk)" (p. 339) the authors suggest that the 'formal' category is limited and they argue that the 'informal' and 'folk' categories should be expanded: "Personal data literacy pedagogy should be expanded to include not only one's own data but also data about one's own experiences, demographic categories to which one belongs and personally impactful social injustices that affect social groups. Expanding definitions of folk pedagogies can encourage creativity in data-informed projects like crafting data stories for the purpose of calling audiences to take action, not

	only as individuals but as members of groups affected by social injustices" (p. 339).<br><br>The authors conclude: "Storytelling is a powerful way to reframe data literacy that has potential to address current challenges, in data literacy pedagogy and in teaching data literacy" (McDowell & Turk 2024, p. 339).<br><br>*End of McDowell and Turk 2024*

Markham 2020

**Charting Elements**	**Associated Questions**
**Publication Details**	Markham, A.N. (2020) 'Taking Data Literacy to the Streets: Critical Pedagogy in the Public Sphere', *Qualitative Inquiry*, Vol. 26 Issue 2: 227-237. https://doi.org/10.1177/1077800419859024
**Study ID**	Markham 2020
**Title**	Taking Data Literacy to the Streets: Critical Pedagogy in the Public Sphere
Author(s)	**Who are the authors of the publication?**<br><br>Markham, Annette, N.
Year of Publication	**When was the paper/study published?**<br><br>2020
Origin/Country of origin	**Where was the study carried out?**<br><br>Denmark
Publication Type	**Is the publication a journal article, book or book chapter, review, opinion paper, grey literature, other?**<br><br>Journal article
**General Overview of Study**	
Aims/purpose	**What were the aims or purpose of the study?**<br><br>*The purpose of the study was to provide a "reflexive meta-analysis" (p.227) (abstract)* of a data literacy

	project entitled the Museum of Random Memory (MoRM)<br><br>“The *Museum of Random Memory* (MoRM) is an ongoing series of performative arts–based public interventions designed to spark deep reflection about the underlying complexities of datafication in everyday digital media usage” (p.227).<br><br>**What was the rationale for using the arts?**<br><br>The focus is on describing the processes of the intervention as a whole and what it is designed to do, rather than discussing a rationale for using the arts.<br><br>“This article describes an ongoing series of public arts-based experiments that build curiosity and develop data literacy via self-reflexive public interventions” (abstract, p. 227).<br><br>“Participants at each MoRM are engaged in an interactive museum experience, where researchers act as “uncurators,” visitors are memory donors, the collective donations of memories are displayed. The practice is framed as an exercise in developing memory archives for future archeologists [sic]. We focus on how the stuff of memory is transformed as it is digitalized and what this might mean on micro and macro scales. Through this playful experience, donor/participants are prompted to reflect on digitalization, datafication, and their own production of big data. The goal of such an intervention is to spark curiosity whereby participants will over time and after their experience at MoRM, seek out and gain stronger digital or data literacy” (p.227).<br><br>The original group aimed to "think about methods for engaging the public in around issues of digitalization, social media, data mining, automation of personal memory, and the future of cultural or civic memory" drawing on their experiences in "activism, art, computer science, museum curation, architecture, filmmaking, printmaking, university administration, law, photography, and computational art" (p.227).
Rationale for Using the Arts in Data Literacy Context Choices: Increase accessibility	

Increase accessibility Yes<br><br>*Increase engagement Yes*<br><br>*Develop critical thinking skills*<br><br>*Other*	
Methodology	**What methodological design was utilized for the study?**<br><br>"Experimentation, Remix Methods, and an Iterative Research Design" (p. 230)<br><br>The MoRM was created using "remix methods" approach (Markham, 2013) (p.230) (building meaning through varied repetition) while this article focused on reflexivity as a form of self and collective-analysis.<br><br>"MoRM aimed to ignite data literacy and critical thinking in the public through participatory experimental interventions. A key element of effective **participatory research design** in the public environment is that each iteration is designed specifically for that situation. In MoRM, this could be described as a "remix methods" approach (Markham, 2013) to enable more playful experimentation than might be otherwise encouraged in traditional academic research environments" (p.230).
**Key findings relating to the role of the arts in data literacy**	
**Methods**	**What specific methods (e.g. qualitative, quantitative, mixed methods) were utilized?**<br><br>"Within an overall framework of experimentation and critical pedagogy, we mix perspectives and techniques from situational analysis, ethnographic interviewing, museum curation, speculative fabulation, participatory design and critical making, user experience studies, qualitative social science, performance art, theater, rhetorical criticism, computational art, big data analytics, and pedagogy" (pp. 230-231).<br><br>**Which art forms were used?**<br><br>Visual arts, music, "architecture, filmmaking, printmaking, photography, computational art (p. 227).

	**Was data literacy defined and, if so, how?**<br><br>"Data literacy is a type of awareness and curiosity that leads to developing competencies needed to grapple with the complex impacts of digital transformation on individual and cultural wellbeing. A critical data literacy is built on the premise that to be critical in any effective or sustained way requires deep understanding of the contexts within which digitalization or datafication is occurring. Data literacy falls into the same concept pool as multi-literacy, digital literacy, information literacy, digital media literacy, and media literacy" (p. 229). [this seems to be her own definition. There's no other citation].
	**Was data literacy measured and, if so, how?**<br><br>"I also recognize that my impulse to lay the burden for effectiveness on the measurable outcome of the experiment is the wrong way about it. Because that is not how art, art-based research endeavors, public interventions, or activism work. In all the installations, the initial prompts change, and the **outcomes can't be measured**. This is the deliberate design. Strong interest, curiosity, and participation result in excellent conversations between social media experts and lay public and between teachers and people interested in researching their own lived experience. But **to reverse engineer these to find any certain causal chain of input and output is impossible"** (p.236).
	**Was there an evaluation of the role of the arts in enhancing data literacy and, if so, how?**<br><br>There was no explicit evaluation of the role of the arts but there was an acknowledgement that the multidisciplinary team (which included arts disciplines) engaged in a process of reflexive adaptation and iterative change.<br><br>The role of the arts in enhancing data literacy is not discussed. The focus is on collecting layers of data and the retrospective assessments and reflections of the research team:<br><br>"Retrospection is key to analytically strengthening the format of the exhibitions and the micro interactions with participants. We don't have access to most of the participants after the fact and although we have gathered contact information from them as a part of

	collecting personal materials from them, we have not found it necessary, yet, to engage in in-depth follow-up with individuals. Rather, we use ourselves and our own reactions to help assess the quality of the prompts. We test it among ourselves as a design and research team, which is generally around 20 people from different countries of origin, ages, ethnicities, level of education, professions, and interests. After each event, we have half or full day meetings to discuss the process, recapture important moments and encounters, and to decompress. Recording these sessions in audio/video, and collecting sketches and notes, we now have layers and layers of data about the process. Whether or not these are ever collated, annotated, or analyzed later is less important than our faith that this documentation is available for us to access later if we need to" (p. 235).
**Study Population**	**What population groups are being studied in the literature?**<br><br>No population groups are specified in terms of participants outside of the research team.
	**What was the sample size?**<br><br>No sample size specified. The exhibition/installation was open to the public in multiple countries: Denmark, Italy, Spain, Canada, and the UK (p. 227).
**Research Setting**	Was it a community-based setting?<br><br>Community-based in multiple locations and countries.<br><br>Was it an educational setting?<br><br>Was it a healthcare setting?
**Findings/results**	**How have the arts have been used to enhance data literacy?**<br><br>"One strong outcome of our experiments, experienced at each of the MoRM events in the past 2 years, is consciousness raising, in the classic sense we might associate with the use of this phrase in the late 1960s as a part of the U.S. women's feminist/liberation movement. The interactions between researchers and participants made possible by the design of the exhibition spark engagement, critical thinking, and curiosity. Participants become more aware of their own *digital media use, such as the scope and size of their production of personal "big data" (p. 235).*

Was the evaluation of the role of the arts positive, negative or mixed?	**Was the evaluation of the role of the arts positive, negative or mixed?**<br><br>The author specifically states that the methodology is not designed to evaluate longterm change:<br><br>“The methodology is not interested in a longterm evaluation on change, but in the momentary and embodied interaction between researcher and participant, and the moments among researchers” (p. 236).<br><br>“The case of MoRM emphasizes how effective public engagement, intended to build digital and data literacy, requires dexterity in finding and continually adjusting statements, provocations, questions, material and digital props, and other prompts that best fits the person, the content of the interaction, and the context of the conversation” (p. 236).<br><br>Markham notes that the goal is not to evaluate, but rather pragmatically to create something that works: "We’re not trying to create best practices for digital or data literacy, but to engage with the public in ways that work" (p. 235).<br><br>*[End of Markham 2020]*

Matuk et al. 2021

**Charting Elements**	**Associated Questions**
**Publication Details**	Matuk, C., DesPortes, K., Amato, A., Silander, M., Vacca, R., Vasudevan, V. and Woods, P.J. (2021) ‘Challenges and opportunities in teaching and learning data literacy through art’<br>*Proceedings of the 15th International Conference of the Learning Sciences-ICLS 2021*.<br>Publisher: International Society of the Learning Sciences.<br>https://repository.isls.org/bitstream/1/7556/1/681-684.pdf
**Study ID**	Matuk et al. 2021
**Title**	‘Challenges and opportunities in teaching and learning data literacy through art’
Author(s)	Who are the authors of the publication?

	Matuk, C., DesPortes, K., Amato, A., Silander, M., Vacca, R., Vasudevan, V. and Woods, P.J.
Year of Publication	When was the paper/study published? 2021
Origin/Country of origin	Where was the study carried out?<br><br>USA – *"private school in the United States" (p.681*
Publication Type	Is the publication a journal article, book or book chapter, review, opinion paper, grey literature, other?<br><br>Conference paper
**General Overview of Study**	
Aims/purpose	**What were the aims or purpose of the study?**<br><br>The purpose was to design and implement an intervention and to use this to explore pedagogical considerations for this approach to data literacy:<br><br>"We designed and implemented a 2-week long arts-integrated unit in a grade 7 classroom. Interviews with two teachers and two students following the unit, and analysis of students' artworks and pre/post survey responses, reveal opportunities and challenges at the intersection of data science and art. We discuss pedagogical considerations for other interdisciplinary approaches to data literacy" (Abstract, p. 681).<br><br>**What was the rationale for using the arts?**<br><br>Several reasons were articulated for using an arts-based approach:<br><br>"Cultivating data literacy is challenging because **school mathematics tends to narrowly focus** on statistical reasoning, and to **present data as objective and devoid of context** (Franklin et al., 2015).<br><br>We explore an approach to **broadening participation** in data science by integrating art and mathematics in a middle school classroom-based unit. This project builds on existing efforts to tie school-based data literacy instruction more closely to **personal and social contexts** (e.g. Kahn, 2020) by incorporating an interdisciplinary focus on art (c.f. Lupi & Posavec, 2016). Interdisciplinary approaches to mathematics and data literacy can be valuable for **engaging students** who are disengaged in mathematics and can support deeper data literacy learning (Stornaiuolo, 2020). It allows learning through the making process through which learners can engage

	in meaningful reflection (Turkle & Papert, 1990). Moreover, data art is a distinct art form that engages audiences with both emotional and intellectual properties of data (D'Ignazio & Klein, 2020; Hall, 2008). In negotiating these priorities, the artist represents the situated nature of data, a fundamental principle of **critical perspectives**" (p. 681).<br><br>*Increase accessibility<br><br>*Increase engagement<br><br>*Develop critical thinking skills<br><br>*Other: highlights personal and social contexts of data
Rationale for Using the Arts in Data Literacy Context Choices: Increase accessibility<br><br>*Increase engagement*<br><br>*Develop critical thinking skills*<br><br>*Other*	
Methodology	**What methodological design was utilized for the study?**<br><br>Co-design and evaluation of an intervention:<br><br>"we co-designed and implemented a unit" (p.681).
**Key findings relating to the role of the arts in data literacy**	
**Methods**	**What specific methods (e.g. qualitative, quantitative, mixed methods) were utilized?**<br><br>Mixed methods:<br><br>"Our data consist of (1) a post-unit **group interview with two students** (whom we name Ariel (female) and Yamil (male)), which asked them to reflect on their experiences during the unit and to elaborate on the decisions they made in creating their artwork; (2) **individual post-implementation interviews with each teacher**, in which we ask them to reflect on how the unit met or failed to meet their learning goals, and on notable observations of student learning; (3) **student artifacts** (artwork, artist statements, and written reflections on their processes); (4) **responses to a pre and post survey** that probed students' engagement and self-perceived competencies in math and art, and their abilities to critique existing

	data-based art; and (5) **researchers' reflections** on our experiences co-designing the unit" (p.682).<br><br>**Which art forms were used?**<br><br>Data drawing and data sculpture. This paper focused on data sculpture:<br><br>"For the purposes of this paper, we focus on students' data sculptures" (P.682).<br><br>Data drawing and data sculpture
	**Was data literacy defined and, if so, how?**<br><br>No explicit definition of data literacy but data art is described:<br><br>"… data art is a distinct art form that engages audiences with both emotional and intellectual properties of data (D'Ignazio & Klein, 2020; Hall, 2008)" (p.681).
	**Was data literacy measured and, if so, how?**<br><br>Data literacy was not measured but the opportunities and challenges of the data art intervention were explored.
	**Was there an evaluation of the role of the arts in enhancing data literacy and, if so, how?**<br><br>Yes = the opportunities and challenges of the data art intervention were explored.
**Study Population**	**What population groups are being studied in the literature?**<br><br>Middle school students and their teachers in a US school. Two students interviewed with implication that one was male and one female.
	**What was the sample size?**<br><br>one art teacher (Kelly, female) and one math teacher (Bruce, male) and their 25 seventh grade students.
**Research Setting**	Was it a community-based setting?<br><br>Was it an educational setting? Yes<br><br>Was it a healthcare setting?

**Findings/results**	**How have the arts have been used to enhance data literacy?**<br><br>Data sculpture was used to engage with and present data related to a specific data set.
Was the evaluation of the role of the arts positive, negative or mixed?	**Was the evaluation of the role of the arts positive, negative or mixed?**<br><br>Mixed<br><br>The use of the arts was seen to have both opportunities and challenges:<br><br>"…we found that students generated unique questions, negotiated relationships between data and materials, and made claims that attempted to go beyond the data." (*p.684)<br><br>"students struggled to communicate personal perspectives that were also grounded in data" (p.284).<br><br>"This shows the difficulties of working with data as an artistic material, that is, in negotiating its affordances and constraints for communicating a perspective to an audience" (p.284).<br><br>"This study highlighted the opportunities in an arts-based approach for allowing students to make inferences that give data personal and social meaning. It also emphasized the need to ensure that students are grounding their interpretations in evidence" (p.284.)<br><br>*[End of Matuk et al. 2021]*

Matuk, Amato et al. 2022a

**Charting Elements**	**Associated Questions**
**Publication Details**	Matuk, C., Amato, A., Davidesco, I., Rubel, L., Stornaiuolo, A., Blikstein, P., Bumbacher, E., Chance, B., DesPortes, K., Eloy, A., Fagan, E., Fuhrmann, T., Gebre, E., Herbel-Eisenmann, B., Jiang, S., Kahn, J., Lim, V., Louie, J., Peralta, L.M., Roy, S., Silander M., St Clair, N., Stephens, L., Stiles, J., Tes, M., Vacca, R., Wagh, A., Wei, X., McBride, C., Wilkerson, M., Wolf, J., and Woods, P.J. (2022a) 'Data Storytelling in the Classroom', *Proceedings of International Conference of the Learning Sciences, ICLS 2022,* pp. 1779-1786.<br><br>https://doi.org/10.22318/icls2022.1779

	https://repository.isls.org/handle/1/8584
**Study ID**	Matuk et al. 2022a
**Title**	'Data Storytelling in the Classroom'
Author(s)	Who are the authors of the publication?<br><br>Matuk, C., Amato, A., Davidesco, I., Rubel, L., Stornaiuolo, A., Blikstein, P., Bumbacher, E., Chance, B., DesPortes, K., Eloy, A., Fagan, E., Fuhrmann, T., Gebre, E., Herbel-Eisenmann, B., Jiang, S., Kahn, J., Lim, V., Louie, J., Peralta, L.M., Roy, S., Silander M., St Clair, N., Stephens, L., Stiles, J., Tes, M., Vacca, R., Wagh, A., Wei, X., McBride, C., Wilkerson, M., Wolf, J., and Woods, P.J.
Year of Publication	When was the paper/study published? 2022
Origin/Country of origin	**Where was the study carried out?**<br><br>USA<br><br>*In the US "in contexts that span K-12 to university" (Abstract, p1779) [just country needed]*
Publication Type	Is the publication a journal article, book or book chapter, review, opinion paper, grey literature, other?<br><br>Conference paper
**General Overview of Study**	
Aims/purpose	What were the aims or purpose of the study?<br><br>It will " examine opportunities to **promote students' data literacy through storytelling** and will **critique the value added** of stories over more conventional approaches to data literacy instruction." (p.1780)<br><br>**What was the rationale for using the arts?**<br><br>With the growing prevalence and importance of data, "it is critical **to find new, more effective ways to develop students' data literacies**. **Stories can be an accessible way** for students to **personally connect** to, and **think critically** about, data and its implications"(Abstract p. 1779)<br><br>"Stories have long been recognized as **tools for sensemaking, identity building** and **empathy** (Bruner, 1985, Cleto & Warman, 2019; Lind & Thomsen, 2018)" (p. 1780).

	*Increase accessibility<br>*Increase engagement<br>*Develop critical thinking skills
Rationale for Using the Arts in Data Literacy Context Choices: Increase accessibility<br>*Increase engagement*<br>*Develop critical thinking skills*<br>*Other*	
Methodology	**What methodological design was utilized for the study?**<br>This is a symposium consisting of, "8 posters in this symposium demonstrate the various roles of storytelling in data literacy education." (p.1780). A short description of each project is included. Each explores the use of stories in supporting data literacy in different educational contexts ranging from K-12 to university.
**Key findings relating to the role of the arts in data literacy**	
**Methods**	**What specific methods (e.g. qualitative, quantitative, mixed methods) were utilized?**<br>In the symposium, the following format was used:<br>"Following introductory remarks by the session chairs, we will provide the audience with guiding questions, and will facilitate two rounds (4 posters each) of interaction among presenters and attendees. By allowing co-presenters to also visit and interact with co-presenters, we seek to encourage rich discussions that cut across issues raised by their own and the audience's projects and experiences" (p.1780).<br>**Which art forms were used?**<br>Storytelling<br>The focus of the symposium is on storytelling

	**Was data literacy defined and, if so, how?**<br><br>Data literacy was not defined but data story telling was:"the term "data storytelling" has been defined as a narrative that offers a causal inference that counters audiences' assumptions (Matei & Hunter, 2021). Others write that good visual data storytelling draws an audience in, and helps to make data more persuasive, easier to understand, and to remember (Ma et al., 2011)" (p. 1780).
	**Was data literacy measured and, if so, how?**<br><br>Aspects of evaluation are mentioned in the project summaries but this was not the focus of this symposium.
	**Was there an evaluation of the role of the arts in enhancing data literacy and, if so, how?**<br><br>Aspects of evaluation are mentioned in the project summaries but this was not the focus of this symposium.
**Study Population**	**What population groups are being studied in the literature?**<br><br>The posters include project examples ranging from K-12 to university settings in the US.
	**What was the sample size?**<br><br>Not all the poster examples mention sample size but some of them do, for example, the project Grappling with data stories of social groups and social justice notes that its data included:<br><br>"Pre- and post-survey data from over 200 students in non-AP statistics classes from seven high schools with high proportions of Blacks and Latinos/as indicate that students who used the modules showed statistically significant growth in their interests in statistics and data analysis" (p.1783).
**Research Setting**	Was it a community-based setting?<br><br>Was it an educational setting? Yes<br><br>Was it a healthcare setting?
**Findings/results**	**How have the arts have been used to enhance data literacy?**<br><br>"the 8 posters in this symposium demonstrate **the various roles of storytelling in data literacy** education. They show that stories can be used to **contextualize and**

	**communicate** about data in everyday life (Gebre); integrate different perspectives to represent and **make sense of environmental events** (Wagh et al.); critically **engage with social inequities** and stereotypes (Louie et al.); **drive scientific exploration** (St. Clair & Stephens); **make sense of aberrant or unexpected data** (Davidesco et al.), **reflect on personal implications** of data (Tes et al.); **reframe sociopolitical crises** (Rubel et al.); and **identify personal connections** to broader data patterns (Wei et al.)" (p.1780).
Was the evaluation of the role of the arts positive, negative or mixed?	**Was the evaluation of the role of the arts positive, negative or mixed?**<br><br>The goal of the symposium was to spark questions about using storytelling in data literacy rather than evaluate it. Some of the questions suggested include the following:<br><br>"What makes a data story a story? How is a data story different from an argument or an explanation? How can stories be used to promote data practices in meaningful and accessible ways to a broad range of learners? What challenges, opportunities, and strategies are relevant when incorporating data storytelling in classroom contexts? What kinds of support—technology based or otherwise—benefit students and teachers in navigating their use of stories for data literacy? How can educators and learning designers leverage the affordances of stories to promote specific data literacies and practices? How can we support students' in moving from exploring data to producing a data story? Likewise, how can we support them in moving from an understanding of data situated in a story's context, to an understanding of the broader implications of data?" (p.1781).<br><br>*[End of Matuk et al. 2022a]*

Matuk, DesPortes et al. 2022b

**Charting Elements**	**Associated Questions**
**Publication Details**	Matuk, C., DesPortes, K., Amato, A., Vacca, R., Silander, M., Woods, P.J. and Tes, M. (2022b) 'Tensions and synergies in arts-integrated data literacy instruction: Reflections on four classroom implementations', *British Journal of Educational Technology*, 53(5): 1159-1178. https://doi.org/10.1111/bjet.13257
**Study ID**	Matuk et al. 2022b

**Title**	'Tensions and synergies in arts-integrated data literacy instruction: Reflections on four classroom implementations'
Author(s)	**Who are the authors of the publication?**<br><br>Matuk, C., DesPortes, K., Amato, A., Vacca, R., Silander, M., Woods, P.J. and Tes, M.
Year of Publication	When was the paper/study published?<br><br>2022
Origin/Country of origin	**Where was the study carried out?**<br><br>USA<br><br>"in four middle schools across three U.S. states" (p.1162)
Publication Type	Is the publication a journal article, book or book chapter, review, opinion paper, grey literature, other?<br><br>Journal article
**General Overview of Study**	
Aims/purpose	**What were the aims or purpose of the study?**<br><br>The aim of this study is to "describe a discipline-integrated approach to data literacy education called **data-art inquiry**, and explore how the intersections between data literacy, technology and the arts create **synergies** that support learning, and **tensions** that hinder it.p.1161<br><br>**What was the rationale for using the arts?**<br><br>The arts have the potential to support data literacy in a number of important ways:<br><br>"Data-art inquiry can support students' informal inference-making by **revealing the role of context** in shaping the meaning of data, and encouraging **consideration of the personal and social relevance** of data. Data-art inquiry additionally **creates alternative entry point**s into data literacy by building on learners' non-STEM interests. Supported by technology, it can **provide accessible tools** for students to reflect on and communicate about data in ways that can **impact broader audiences** (Abstract p.1159).<br><br>"Drawing on arts-based approaches (Blumenfeld-Jones, 2016), data-art inquiry engages creators and audiences in **thinking critically** about context, and in revealing

	personal or social implications that may otherwise be invisible" (p. 1161).<br><br>*Increase accessibility<br><br>*Increase engagement<br><br>*Develop critical thinking skills<br><br>*Other: reveal context, personal/social relevance
Rationale for Using the Arts in Data Literacy Context Choices: Increase accessibility<br><br>*Increase accessibility*<br><br>*Increase engagement*<br><br>*Develop critical thinking skills*<br><br>*Other*	
Methodology	**What methodological design was utilized for the study?**<br><br>"We worked with 10 teacher partners in four middle schools across three U.S. states to co-design and implement four data-art inquiry curriculum units: *Dance, Photoessays, Comics*, and *Collages.*" *(*p.1162)
**Key findings relating to the role of the arts in data literacy**	
**Methods**	**What specific methods (e.g. qualitative, quantitative, mixed methods) were utilized?**<br><br>Mixed methods: "To elicit our insights, we iterated on a shared narrative of our experiences by triangulating between **classroom data**, **participant interviews** and **co-design meeting notes"** (p.1164).<br><br>"Classroom data varied based on school-specific IRB permission, and included student **artefacts** (artworks, written responses to reflective prompts), **classroom observations**, **pre-post surveys**, and **student interviews**." (p.1164)<br><br>NOTE: "The implementations occurred during COVID-19 pandemic school restrictions. All classes had a varying mix of in-person and remote students joining via video conference" (p.1163).<br><br>**Which art forms were used?** Visual arts and Dance: Dance, photography, comics, collage

	**Was data literacy defined and, if so, how?**<br><br>Data literacy was not defined but a distinction was drawn between data science education and data literacy: “data science education prepares learners for careers that require specialized skills in generating, manipulating, and analysing data; data literacy education aims to prepare learners in all roles of an increasingly datafied society (Bargagliotti et al., 2020; Pangrazio & Sefton-Green, 2022; Wilkerson & Polman, 2020)” (pp. 1160-1161).
	**Was data literacy measured and, if so, how?**<br><br>The paper implies that evaluation of data literacy was carried out and published in their other papers but not in this paper, which focused on synergies and challenges in data-art inquiry.
	**Was there an evaluation of the role of the arts in enhancing data literacy and, if so, how?**<br><br>As above- The paper implies that evaluation of data literacy was carried out and published in their other papers but not in this paper, which focused on synergies and challenges in data-art inquiry.
**Study Population**	**What population groups are being studied in the literature?**<br><br>*“*10 teacher partners in four middle schools across three U.S. states” (p.1163).
	**What was the sample size?**<br><br>10 teachers in four schools. Exact numbers of students not supplied.
**Research Setting**	Was it a community-based setting?<br><br>Was it an educational setting? Yes<br><br>Was it a healthcare setting?
**Findings/results**	**How have the arts have been used to enhance data literacy?**<br><br>The arts were used in a number of different ways:<br><br>“In terms of synergies between data literacy and the arts, we found **different media to offer unique ways of supporting informal statistical reasoning** (Makar & Rubin, 2018). For example, narrative-**based media (comics and creative writing) supported students'**

	**introspection on their personal experiences** relative to broader data trends. **Movement-based art (dance) supported embodied sensemaking** of the shape and meaning of data. Meanwhile, **photography** highlighted how data should be interpreted within the limits of operationalization and sampling decisions, and **how images can reveal contextual details that tend to be hidden in statistics.** However, there were also **tensions** in using arts-based approaches to convey data claims as students struggled in making artistic choices based on **data vs. aesthetics**" (p 1174).<br><br>"Importantly, data-art is **more than presenting graphs in visually appealing ways**. It often involves physicalizing data in ways that invite the audience's critical attention to issues through datap" (p. 1162).
Was the evaluation of the role of the arts positive, negative or mixed?	**Was the evaluation of the role of the arts positive, negative or mixed?**<br><br>The article reports on both synergies and tensions in the data-technology-arts triangulation I data literacy. Regarding the arts, these were viewed as primarily positive with some tensions regarding balancing aesthetics with accuracy.<br><br>"In terms of synergies between data literacy and the arts, we found different media to offer unique ways of supporting informal statistical reasoning (Makar & Rubin, 2018)" (p.1174).<br><br>"These reflections highlight a tension between the goals of data literacy and the arts, and the need to guide students in finding disciplinary alignments that balance aesthetics with accuracy" (p.1167).<br><br>"Arts-based practices and routines furthermore encouraged students' engagement with critical issues about data" (p.1167)<br><br>*[End of Matuk, DesPortes et al. 2022b]*

Matuk et al. 2024

**Charting Elements**	**Associated Questions**
**Publication Details**	Matuk, C., Vacca, R., Amato, A., Silander, M., DesPortes, K., Woods, P.J., and Tes, M. (2024) 'Promoting students' informal inferential reasoning through arts-integrated data literacy education', *Information and Learning Sciences*, Emerald Insight. np https://doi.org/10.1108/ILS-07-2023-0088

	NOTE – new from search alert (2023)
**Study ID**	Matuk et al. 2024
**Title**	'Promoting students' informal inferential reasoning through arts-integrated data literacy education'
Author(s)	Who are the authors of the publication?<br><br>Matuk, C., Vacca, R., Amato, A., Silander, M., DesPortes, K., Woods, P.J., and Tes, M. (2023)
Year of Publication	When was the paper/study published? 2023
Origin/Country of origin	Where was the study carried out?<br><br>USA<br><br>In four schools (one public charter, one private and two public schools, across three states in the USA).
Publication Type	Is the publication a journal article, book or book chapter, review, opinion paper, grey literature, other?<br><br>Journal article
**General Overview of Study**	
Aims/purpose	**What were the aims or purpose of the study?**<br><br>"The purpose of this study is to describe synergies and tensions between data science and the arts, and how these can create or constrain opportunities for learners to engage in IIR" (Abstract, p.1 in PDF)<br><br>**What was the rationale for using the arts?**<br><br>"Integrating data science with arts has the potential to **foster students' IIR,** as these disciplines share practices of exploring, **sensemaking, critiquing** and **communicating** about issues" (p. 3 in PDF)<br><br>"The arts can support the three interrelated processes of IIR" (p. 4 in PDF). These include going beyond the data' using data as evidence for generalisations; expressing uncertainty about generalisations.<br><br>*Increase accessibility<br><br>*Increase engagement<br><br>*Other: communicating
Rationale for Using the Arts in Data Literacy Context Choices: Increase accessibility	

Increase accessibility<br><br>*Increase engagement*<br><br>*Develop critical thinking skills*<br><br>*Other*	
Methodology	**What methodological design was utilized for the study?**<br><br>Co-design and evaluation of data-art interventions
**Key findings relating to the role of the arts in data literacy**	
**Methods**	**What specific methods (e.g. qualitative, quantitative, mixed methods) were utilized?**<br><br>Mixed methods including:<br><br>**Post-implementation interviews** with each of the 10 teachers. These 1–2 h-long<br><br>**teacher-generated artifacts**, including presentation slides, exit tickets and written postclass reflections;<br><br>**student-generated artifacts**, including their data artworks and artist statements<br><br>**interviews with three to four focal students per implementation**, which asked students to reflect on their processes and artistic decisions, and to explain the connections between their artworks and their interpretations of the data (p.8 of PDF)<br><br>**Which art forms were used?**<br><br>*Dance, Photoessays, Comics and*<br><br>*Collages* (p.7 PDF)
	**Was data literacy defined and, if so, how?**<br><br>No but, informal, inferential reasoning is defined.<br><br>"IIR is defined as "the way in which students use their informal statistical knowledge to make arguments to support inferences about unknown populations based on observed samples" (Zieffler et al., 2008, p. 44). (p.2 on PDF)
	**Was data literacy measured and, if so, how?**<br><br>Aspects of evaluation were included in the individual interventions but these were not the focus of this paper.

	**Was there an evaluation of the role of the arts in enhancing data literacy and, if so, how?**<br><br>Aspects of evaluation were included in the individual interventions but these were not the focus of this paper.
**Study Population**	**What population groups are being studied in the literature?**<br><br>"The four schools served diverse student populations that varied from 2 to 65% White and 25 to 95% non-White students, and 40 to 86% in economic need as defined by eligibility for free and reduced price lunch. Each of the classrooms in which we implemented the units included 15–30 students" (p. 6 PDF).
	What was the sample size?<br><br>Exact sample size not specified: 10 teachers recruited to co-design the project. *Approximate* number of students specified: 15-30 students in each of four schools.
**Research Setting**	Was it a community-based setting?<br><br>Was it an educational setting? Yes<br><br>Was it a healthcare setting?
**Findings/results**	**How have the arts have been used to enhance data literacy?**<br><br>"we posit **four data–art inquiry practices observed to support or constrain students'** engagement in IIR" (p. 17 in PDF).<br><br>*"Use visual or performed narratives to contextualize data relationships" (p. 18 in PDF): narrative helpful in personalising data but can be challenging in presenting more generalised patterns or uncertainty.<br><br>* "Draw on personal experiences to engage with uncertainty in relating local to global data" (p. 18 in PDF): personal experience can support engagement with big ideas but may overemphasise the subjective<br><br>* "Use aesthetic representations to elevate emotion in communicating about data" (p. 19 in PDF): the arts can support and enhance the emotional communication concerning data but care must be taken that aesthetic and emotive representations remain evidence based.<br><br>* "Use audience considerations to reflect on the nonneutrality of data" (p. 20 of PDF). – considering how others might interpret the communication of data

	reinforces the non-neutrality and contextual nature of data.
Was the evaluation of the role of the arts positive, negative or mixed?	Was the evaluation of the role of the arts positive, negative or mixed?<br><br>See above – the arts are seen to both support and constrain students.<br><br>*[End of Matuk et al. 2023]*

Nguyen & Parameswaran 2023

**Charting Elements**	**Associated Questions**
**Publication Details**	Nguyen, H., and Parameswaran, P. (2023) 'Meaning making and relatedness: exploring critical data literacies on social media', *Information and Learning Sciences,* Vol. 124 No. 5/6, pp. 149-167. https://doi.org/10.1108/ILS-02-2023-0016
**Study ID**	Nguyen & Parameswaran 2023
**Title**	'Meaning making and relatedness: exploring critical data literacies on social media',
Author(s)	**Who are the authors of the publication?**<br><br>**Nguyen, H., and Parameswaran**
Year of Publication	**When was the paper/study published?**<br><br>2023
Origin/Country of origin	**Where was the study carried out?** Online (TikTok)<br><br>Authors are U.S.A.<br><br>"Most creators were based in the USA, with a few in Australia, Canada, Sweden, the UK, and the Philippines" (fifth page np).
Publication Type	**Is the publication a journal article, book or book chapter, review, opinion paper, grey literature, other?**

	Journal article
**General Overview of Study**	
Aims/purpose	**What were the aims or purpose of the study?**<br><br>"The goal of this study is to explore how content creators engage in critical data literacies on TikTok … This work focuses on how TikTok content creators repurpose data to construct their own narratives" (Abstract, p.149).<br><br>Also, to explore the technical features (auditory, textual, visual) of TikTok which support data practices:<br><br>"Researchers have studied how TikTok's **auditory, textual, and visual features** allow for expressing voices … Although scholars have mentioned the potential of TikTok to elevate experiences as valuable data points (Calabrese Barton et al., 2021), **there is limited research on how the platform's technical features invite for data practices**. We aim to address this gap (p.152).<br><br>The focus was on videos related to environmental and climate action.<br><br>**What was the rationale for using the arts?**<br><br>TikTok is a social platform which makes distinctive use of "**video** features (audio, footage, background images) that may support these practices" (Abstract, P.149).<br><br>*Access accessibility<br><br>*Increase engagement
Rationale for Using the Arts in Data Literacy Context Choices: Increase accessibility<br><br>*Increase accessibility*<br><br>*Increase engagement*<br><br>*Develop critical thinking skills*<br><br>*Other*	
Methodology	**What methodological design was utilized for the study?**<br><br>"Through **hashtag search**, the authors created a corpus of 410 TikTok videos focused on discussing environmental and climate action, and **qualitatively coded the videos** for data literacies practices and video features (audio, footage, background images) that may support these practices" (p.149 Abstract).

**Key findings relating to the role of the arts in data literacy**	
**Methods**	**What specific methods (e.g. qualitative, quantitative, mixed methods) were utilized?**<br>Qualitative analysis of TikTok videos<br>"We qualitatively coded the videos for data practices and video features. For data practices, we used thematic analysis (Braun and Clarke, 2006) to develop inductive codes" (p.154).<br>**Which art forms were used?**<br>Video, particularly the video features of audio, footage and background images.<br>• Video images / photography<br>• Video music / audio<br>• Video
	**Was data literacy defined and, if so, how?**<br>Yes:<br>"Data literacy encompasses the ability to "identify, collect, evaluate, analyze, interpret, present, and protect data" (Oceans of Data Institute, 2016, p. 4)" (p.150).<br>A definition / description of "critical data literacies" was also included:<br>"This dual position requires critical data literacies, which entail the ability to read and reason with data, critique the implications of data use, and communicate to external audiences in ways that contextualize data in personal and social settings (D'Ignazio and Bhargava, 2015; Pangrazio and Selwyn, 2019) " (p.149).
	**Was data literacy measured and, if so, how?**<br>No, the purpose of the study was to evaluate data *practices* in the video corpus (p.155).
	**Was there an evaluation of the role of the arts in enhancing data literacy and, if so, how?**<br>There was no explicit evaluation of the role of the arts but the analysis showed that creating **narratives** from the data was a common practice:

	“Overall, we found multiple overlapping data practices in the video corpus. **Compiling data to create narratives emerged as a common theme** (p.155).
**Study Population**	**What population groups are being studied in the literature?**<br><br>The videos analysed captured:<br><br>“ both individual content creators across age range (66% of profiles in our corpus are between 16 and 30 years old; 10% are older than 30) and institutions (24% of profiles), including environmental nonprofits, news, and eco-minded businesses” (p.150).
	**What was the sample size?**<br><br>The study included a sample of 410 TikTok videos<br><br>(p.150)
**Research Setting**	Was it a community-based setting?<br><br>Was it an educational setting?<br><br>Was it a healthcare setting?<br><br>**Other** – online setting (TikTok) *
**Findings/results**	**How have the arts have been used to enhance data literacy?**<br><br>Different features of video were used in different ways to enhance communication of data:<br><br>E.g. “TikTok allowed creators to record “original **audio**” (e.g. talking and singing), combine original audio with existing audio from others’ speeches (i.e. “existing or mixed audio” code), or just include a soundtrack (i.e. “no audio” code). These different choices allowed users to creatively layer video content with meanings (Literat and Kligler-Vilenchik, 2019). As an example, a video combined an instrumental soundtrack with the creator’s high-pitched scream to indicate their frustration, as they stood in a grocery aisle full of products with plastic packaging (p.157).<br><br>“Regarding **footage,** video creators employed different footage choices, namely recording original footage (n = 361; 88%), using existing footage from other videos (n = 16; 4%), and mixing original and existing footage (n = 33; 8%). For original footage, more than 90% of videos featured the creators talking or moving in front of the camera. Additionally, creators curated footage from

	different sources, such as putting different TikTok videos together or combining news reports with footage of themselves" (p.158).<br><br>"Creators further leveraged the platform's technical features to position themselves in front of different **images**. We focused on images relevant to environmental action and sustainability practices, including screenshots of news articles and policy reports (n = 41; 10% of total videos), depiction of nature through images of plants, animals, and sceneries (n = 70; 17%), and eco-friendly and sustainability artifacts (n = 86; 21%). The ability to add images provided another layer for meaning making" (p.158).<br><br>"In sum, we documented instances where the platform features provided opportunities for content creators to **express experiences**, **build on collective discourse**, and **contextualize the discussion** in a relatable space **for their audiences**"(p.158).
Was the evaluation of the role of the arts positive, negative or mixed?	**Was the evaluation of the role of the arts positive, negative or mixed?**<br><br>Broadly positive:<br><br>The arts (in the form of video features including audio, footage and images) were seen as having the potential to support critical data practices:<br><br>"The current research illustrates that certain technical features may facilitate critical data practices. TikTok's emphasis on reusing and transforming audio, footage, and images can provide authentic opportunities for combining, situating, and positioning data.(p.160)<br><br>"Insights from this research can inform how we might enrich critical data literacies in educational settings" (p.160)<br><br>*[End of Nguyen & Parameswaran 2023]*

Ortega et al. 2023

**Charting Elements**	**Associated Questions**
**Publication Details**	Ortega, A.G., Bourgeois, J. and Kortuem, G., 2023, October. Personal data comics: A data storytelling approach supporting personal data literacy.<br>In *Proceedings of the XI Latin American Conference on Human Computer Interaction* (pp. 1-8).

**Study ID**	Ortega et al. 2023
**Title**	Personal data comics: A data storytelling approach supporting personal data literacy
Author(s)	**Who are the authors of the publication?**<br><br>Gómez Ortega, A., Bourgeois, J. and Kortuem, G.
Year of Publication	**When was the paper/study published?**<br><br>2023
Origin/Country of origin	**Where was the study carried out?**<br><br>Netherlands
Publication Type	**Is the publication a journal article, book or book chapter, review, opinion paper, grey literature, other?**<br><br>Conference paper
**General Overview of Study**	
Aims/purpose	**What were the aims or purpose of the study?**<br><br>The study involved two complementary studies about pregnancy "to investigate (1) how independently creating data comics fosters personal data literacy among non-data experts and (2) how effective and engaging are data comics when compared to annotated data visualizations" (Ortega 2023, sec 5).<br><br>**What was the rationale for using the arts?**<br><br>The authors argue that promoting personal data literacy is critical for supporting "better-informed decisions around sharing and donating personal data" (Ortega et al. sec 1). They note that data visualizations have been successfully used, in multiple contexts, to develop personal data literacy, but they tend to draw on a single source of information and "fail to capture people's situated knowledge" (Ortega et al. 2023 abstract). In this study, three "*non-data experts*" (Ortega et al. 2023, sec 2) were invited to create personal data

	comics about their pregnancies. The authors note that data comics are traditionally created by data *experts.* The data comic approach is noted as "approaching personal data as a probe that elicits memories and reflections on lived experience" (Ortega et al. 2023, sec 2.3.2). The data comics enabled the women to create stories that merged different data types from different sources with their lived experiences. The data comics provided a visual story, and "[u]nlike data, which hides behind its complexity, data comics are clear and easy to digest" (Ortega et al. 2023 sec 2.3.2).
Increase accessibility<br><br>*Increase engagement*<br><br>*Develop critical thinking skills*<br><br>*Other*	Increase accessibility [participants had no previous experience of data visualizations]<br><br>Increase engagement<br><br>To foster greater understanding about how data is intertwined with lived experience
Methodology	What methodological design was utilized for the study?<br><br>Qualitative research design
**Key findings relating to the role of the arts in data literacy**	
**Methods**	**What specific methods (e.g. qualitative, quantitative, mixed methods) were utilized?**<br><br>Mixed methods: a qualitative exploration of the data comic creation process [effectiveness and engagement] and "a quantitative between-subject study with 34 participants" (Ortega et al. 2023, sec 1) using an online survey to ascertain how the data comics were perceived by members of the public.<br><br>Data comic creation sessions on Zoom platform using Miro [online whiteboard tool]: audio recording and participants' independent comic creations over three time periods: a day, a week, a month. Sessions were between 65 and 95 minutes long.<br><br>Qualitative analysis of recorded audio transcripts of comic creation sessions and data comics using reflexive thematic analysis combined with analysis

	of quantitative survey data measuring effectiveness and engagement with the data comics.<br><br>**Which art forms were used?**<br><br>Visual arts: digital art, data comics, storytelling.
	**Was data literacy defined and, if so, how?**<br><br>The authors draw on Gray et al. 2018 and Wolff et al. 2017) for their definition:<br><br>"This term [data literacy] has different meanings in different contexts, but broadly it is about the knowledge and skills a person must have to successfully manage and navigate (personal) data ecosystems of which she is part" (Ortega et al. 2023, sec 1).
	**Was data literacy measured and, if so, how?**<br><br>Data literacy was not measured.
	**Was there an evaluation of the role of the arts in enhancing data literacy and, if so, how?**<br><br>The personal data literacy aspect of study 1 (comic creation) was evaluated qualitatively using reflexive thematic analysis.<br><br>The evaluation (study 2) evaluated how the personal data comics were "perceived and understood by the general public" (Ortega 2023, sec 5).
**Study Population**	**What population groups are being studied in the literature?**<br><br>Women in their early 30s and 34 online participants [the general public] in the evaluation study, ranging in age from 21 to 59, 17 women and 17 men.
	**What was the sample size?**<br><br>Study 1 (creation of data comics) 3 women (the authors acknowledge the small sample size but

	specify that the women shared intimate rich personal data about their pregnancies).<br><br>Study 2: Evaluation survey – 34 participants<br><br>Total: 37
**Research Setting**	**Was it a community-based setting? Yes**<br><br>Was it an educational setting?<br><br>Was it a healthcare setting?
**Findings/results**	**How have the arts have been used to enhance data literacy?**<br><br>Participants in the project created data comics combining data with their lived experiences as a means to enhance personal data literacy.
	**Was the evaluation of the role of the arts positive, negative or mixed?**<br><br>**Mixed:**<br><br>Following the analysis of the survey data, the researchers "could not find evidence that data comics were more effective than annotated data visualizations" (Ortega et al. 2023, sec 3.4) but both were deemed to be efficient and accurate.<br><br>The data comics helped participants to "demystify the perceived complexity around personal data and understand the way data is intertwined with their lived experiences" (Ortega 2023, sec 2.4).<br><br>The authors conclude that the results show that "*non-data experts* can successfully create engaging personal data comics and develop their personal data literacy in the process" (Ortega 2023, sec 5).<br><br>*End of Ortega et al. 2023*

Otani 2022

**Charting Elements**	**Associated Questions**
**Publication Details**	Otani, M.E.S. (2022) '"Track-track: Let's follow the cat!" Reflecting on children's biometric data processing through a micro puppet show', *Proceedings of Interaction Design and Children, IDC 2022*: 629-632. https://doi.org/10.1145/3501712.3535275
**Study ID**	Otani 2022
**Title**	'"Track-track: Let's follow the cat!" Reflecting on children's biometric data processing through a micro puppet show'
Author(s)	**Who are the authors of the publication?**<br><br>Otani, M.E.S.
Year of Publication	**When was the paper/study published?**<br><br>2022
Origin/Country of origin	**Where was the study carried out?**<br><br>**Austria.** University of Art and Design Linz, Linz, Austria. In collaboration with Zurich University of the Arts, Zurich, Switzerland. (Presented in Linz and at 17th Athens Digital Arts Festival).
Publication Type	**Is the publication a journal article, book or book chapter, review, opinion paper, grey literature, other?** Conference paper
**General Overview of Study**	
Aims/purpose	**What were the aims or purpose of the study?**<br><br>Children do not always understand the implications of interacting with data. This paper reports on the DADA-TATA initiative which was developed to address this gap through the inclusion of a cultural /artistic dimension in the project:<br><br>"A UNICEF report asserts **the risks affecting children's rights, especially their right to privacy**, and identifies three main concerns: 1. Children's lack of knowledge about the handling of their personal data; 2. The online surveillance by governments; and 3. The use of biometrics" (p.629).

	“This interactive puppet show **explores how children can recognize themselves as a data source** and perceive **their own digital data as a resource”** (p.629).<br><br>**What was the rationale for using the arts?**<br><br>“The DADA-TATA project believes that a **cultural agenda can complement the educational efforts on data literacy** by making visible – through cultural actions – the changes, risks, and challenges children face in the digital age. This project is **artistic/scientific research** that reflects on children’s rights in the digital realm” (p.629).<br><br>*Increase accessibility*<br><br>*Increase engagement*<br><br>*Develop critical thinking skills*<br><br>* (more accessible to children)<br><br>* (puppet show provides an enjoyable entry point)<br><br>* develop understanding of one’s self as a data source and resource.
Rationale for Using the Arts in Data Literacy Context Choices: Increase accessibility<br><br>*Increase accessibility Yes*<br><br>*Increase engagement Yes*<br><br>*Develop critical thinking skills Yes*<br><br>*Other*	
Methodology	**What methodological design was utilized for the study?**<br><br>“Interaction Design” is mentioned as a concept (p. 629)<br><br>Design of a performance experience (puppet show):<br><br>“The micro puppet show was developed with three main questions in mind: How can children recognize themselves as data subjects? How to make children’s data visible to them creatively? And, how can children be shown that their biometric data is valuable?” (p.630).
**Key findings relating to the role of the arts in data literacy**	

**Methods**	**What specific methods (e.g. qualitative, quantitative, mixed methods) were utilized?**<br><br>Mixed.<br><br>The project is described as “artistic/scientific research that reflects on children’s rights in the digital realm” (p. 629).<br><br>“While seeing the [micro puppet] show inside a box through a peephole, the spectator activates sensors that take the data to make the stars shine, blow the wind, and move the waves” (p. 629).<br><br>Data collection via sensors e.g. heartbeat sensor. Hand-crafted artwork Qualitative reflection on the design process.<br><br>“This paper shares the design process and the audience’s experience of Track-track: Let’s follow the cat!, a micro puppet show where the spectator’s biometric data animate elements of the scenography. This puppet show explores the creation of interactive artworks that reflect on children’s data processing and their right to privacy. Track-track: Let’s follow the cat! is ongoing research with many points to continue working on” (p. 631).<br><br>**Which art forms were used?**<br><br>Puppetry<br><br>• Puppetry (as part of a theatrical show); ‘peep box’ a metaphor for privacy<br><br>• Narrative (story of the cat and mice)and Poetry: “Before the performance, the artist introduces each sensor to the spectator and mentions that the collected data will be used to animate elements of the scenography. This short poem expresses the essence and intention of the puppet show: My heart moves the ocean, my breathing is as strong as the wind, my eyes make the stars shine” (p.630).<br><br>• Handmade art work/handcrafted scenography (p. 631)
	**Was data literacy defined and, if so, how?**<br><br>No, but a description if given of data processes:<br><br>“data processing includes processes of data collection, recording, retention, analysis, dissemination and use” (p.630).

	**Was data literacy measured and, if so, how?**<br><br>Data literacy was not measured – the goal of the research was to present a project design.
	**Was there an evaluation of the role of the arts in enhancing data literacy and, if so, how?**<br><br>Not explicitly but reflections of audience response included some evaluation of the role of the arts including (p. 631)<br><br>-“appreciation of a hand-made art work” (p. 631).<br><br>-creation of an “immersive experience” (p. 631) (micro-world of peep box)<br><br>-appreciation of how the “spectator data” (p. 631) enhanced the experience:<br>“The use of the spectator data ‘improves’ the show’s experience, as evidenced by some comments: One spectator mentioned, “the data somehow personalizes your own puppet show”” (P.631).
**Study Population**	**What population groups are being studied in the literature?**<br><br>The project premiered at Arts Electronica 2020 Festival and subsequently presented at Arts Electronica Center Kids’ Research Laboratory,6 Kunstuniversität Linz, and the 17th Athens Digital Arts Festival” (p.631).<br><br>“Track-track: Let’s follow the cat! Mainly addresses **preschoolers and elementary school children;** however, it also seeks to **involve children’s adult companions**, such as **parents** and **teachers**” (p.632).
	**What was the sample size?**<br><br>“the puppet show reached 71 children and 158 adult spectators in 2020 and 2021” (p.631).
**Research Setting**	Was it a community-based setting? * (festival)<br><br>Was it an educational setting? * research laboratory<br><br>Was it a healthcare setting?
**Findings/results**	**How have the arts have been used to enhance data literacy?**<br><br>This project focused on the use of the arts in the design of a data project – further research needs to be done to explore questions of data literacy:

	“Further research will be conducted to explore the potential of Track-track: Let’s follow the cat! as a medium to foster children’s data literacy*”* (p.632).
Was the evaluation of the role of the arts positive, negative or mixed?	**Was the evaluation of the role of the arts positive, negative or mixed?**<br>Not evaluated as positive or negative but described as part of a project design to make children’s data more visible:<br>“In this preliminary stage, the answered question is “How to make children’s data visible to them creatively?” Track-track: Let’s follow the cat! achieves **making children’s data visible through a traditional artistic experience** such as a micro puppet show **revealing its potential** as a medium to reflect on children’s biometric data processing” (p.632).<br>*[End of Otani 2022]*

Otani 2024

**Charting Elements**	**Associated Questions**
**Publication Details**	Otani, M.E.S., 2024, June. “Beehive” Interactive Installation for Playgrounds: Reflecting on Children's Rights in the Context of Big Data Industry. In *Proceedings of the 23rd Annual ACM Interaction Design and Children Conference* (pp. 969-972).
**Study ID**	Otani 2024
**Title**	“Beehive” Interactive Installation for Playgrounds: Reflecting on Children's Rights in the Context of Big Data Industry
Author(s)	**Who are the authors of the publication?**<br>Otani, M.E.S.
Year of Publication	**When was the paper/study published?**<br>2024
Origin/Country of origin	**Where was the study carried out?**<br>Austria

Publication Type	**Is the publication a journal article, book or book chapter, review, opinion paper, grey literature, other?**<br><br>Conference paper
**General Overview of Study**	
Aims/purpose	**What were the aims or purpose of the study?**<br><br>The paper reports on an artistic playground installation prototype that uses the metaphor of bees and honey production to help improve children's understanding of data collection and processing. The project explored "how the big data sector, particularly YouTube, threatens children's rights during their free time and play" (Otani 2024, p. 969).<br><br>**What was the rationale for using the arts?**<br><br>The authors set out a context stating that the world is experiencing increased datafication, and that children "are subject to data collection practices that are a major concern as it could potentially infringe upon their rights in the digital realm" (Otani 2024, p. 969).<br><br>*Beehive* is part of the DADA-TATA project for which Track-*track: Let's follow the cat!* a micro-puppet show was the first output. *Beehive* is the second output from the DADA-TATA project's with the [educational] aim " to promote children's rights in the digital environment and data literacy by creating interactive artworks for young audiences (especially for those aged 5-8)" (Otani 2024, 969).
Increase accessibility<br><br>*Increase engagement*<br><br>*Develop critical thinking skills*<br><br>*Other*	Increase accessibility<br><br>Increase engagement
Methodology	What methodological design was utilized for the study?<br><br>Artistic installation prototype development

**Key findings relating to the role of the arts in data literacy**	
**Methods**	What specific methods (e.g. qualitative, quantitative, mixed methods) were utilized?<br><br>Qualitative<br><br>The interactive installation explored three research questions:<br><br>"How can children recognize themselves as data subjects?<br><br>How to make children's data visible to them creatively?<br><br>How can children be shown that their generated data is valuable?" (Otani 2024, p. 970).<br><br>The *Beehive* installation used sensors and motors to convert "children's physical movements and play into digital interactions […] transforming these playful activities into online quantitative data" (Otani 2024, p. 971).<br><br>**Which art forms were used?**<br><br>An interactive artistic playground installation
	**Was data literacy defined and, if so, how?**<br><br>Data literacy was not defined.
	**Was data literacy measured and, if so, how?**<br><br>Data literacy was not measured.
	**Was there an evaluation of the role of the arts in enhancing data literacy and, if so, how?**<br><br>The paper discusses the development and potential of the *Beehive* prototype.
**Study Population**	What population groups are being studied in the literature?<br><br>*Future* target group, children aged 3-5 years.

	**What was the sample size**<br><br>N/A Prototype development
**Research Setting**	**Was it a community-based setting?** Yes<br><br>Was it an educational setting?<br><br>Was it a healthcare setting?
**Findings/results**	**How have the arts have been used to enhance data literacy?**<br><br>The author describes the *Beehive* artistic playground installation prototype as a means to enhance digital, and consequently, data literacy. The installation incorporates three sensors disguised as bees in playground seesaws, swings, and slides. Sensors detect the movement of children who interact with the playground equipment "activating motors that randomly scroll content from the YouTube Kids app and play videos on mobile phones housed within a beehive shaped box" (Otani 2024, p. 970)
	**Was the evaluation of the role of the arts positive, negative or mixed?**<br><br>Positive in terms of the potential of the project.<br><br>The author notes that *Beehive* has the potential to raise awareness and "empower children to make informed choices, and enable them to take control of their personal data" (Otani 2024, p. 971).<br><br>"The approach of *Beehive* interactive installation ensures the project contributes to both artistic and educational conversations while advocating a more secure an informed digital landscape for children" (Otani 2024, p. 971). The author argues that art is a powerful medium to increase awareness of children's rights.<br><br>The author suggests that *Beehive* can foster digital literacy and improve awareness of children's rights.<br><br>The author notes that the installation is in its prototype stage and has yet to be formally tested. The next phase will incorporate design-based research.

	End of Otani 2024

Payne et al. 2021

**Charting Elements**	**Associated Questions**
**Publication Details**	Payne, W.C., Bergner, Y., West, M.E., Charp, C., Shapiro, R.B., Szafr, D.A., Taylor, E.V. and DesPortes, K. (2021) 'DanceOn: Culturally responsive creative computing', *Conference on Human Factors in Computing Systems - Proceedings 2021* https://doi.org/10.1145/3411764.3445149
**Study ID**	Payne et al. 2021
**Title**	DanceOn: Culturally responsive creative computing
Author(s)	**Who are the authors of the publication?**<br><br>Payne, W.C., Bergner, Y., West, M.E., Charp, C., Shapiro, R.B., Szafr, D.A., Taylor, E.V. and DesPortes, K.
Year of Publication	**When was the paper/study published?**<br><br>2021
Origin/Country of origin	**Where was the study carried out?**<br><br>USA
Publication Type	**Is the publication a journal article, book or book chapter, review, opinion paper, grey literature, other?**<br><br>Conference paper
**General Overview of Study**	
Aims/purpose	**What were the aims or purpose of the study?**<br><br>"danceON (dance Object Notation) is an educationally-focused programming system for creating visual animations that respond to data from body movement. The system was developed as part of an ongoing Design-Based Research collaboration [6, 83] with a community organization, STEM From Dance[1] (SFD). Our shared goal is to engage young women of color in creating artistic

	computational artifacts within culturally relevant dance learning experiences" (Section 1).<br><br>Core principles: "1. danceON should be personally and culturally relevant to learners, 2. it should situate art and code as mutually informing, and 3. it should deeply support embodied learning of computer science concepts" (Section 3.3).<br><br>**What was the rationale for using the arts?**<br><br>"Creative or artistic computing can promote reflection on the intent and perception of a computational art piece and generate shareable artifacts that embody learners' experiences, identities, and communities [46]. Data-driven artistic education has potential to expand our notion of sense-making and how it can occur [60]" (Section 1).<br><br>"danceON aims to foster computing literacy by bridging dance and technology in ways that empower users to create innovative and expressive pieces with minimal barriers to entry. To achieve this goal, we draw on innovations from culturally relevant education, embodied learning, dance and computing education, and work exploring the design of programming languages". [Section 2]<br><br>*increase accessibility<br><br>*develop critical thinking skills<br><br>*increase engagement
Rationale for Using the Arts in Data Literacy Context Choices: Increase accessibility<br><br>*Increase engagement*<br><br>*Develop critical thinking skills*<br><br>*Other*	
Methodology	**What methodological design was utilized for the study?**<br><br>"We developed danceON as part of a collaborative Design-Based Research investigation [6, 83] with STEM From Dance. SFD is a community organization that creates dance and computing experiences intended to introduce young women of color to STEM fields" (section 3.1).
**Key findings relating to the role of the arts in data literacy**	

**Methods**	**What specific methods (e.g. qualitative, quantitative, mixed methods) were utilized?**<br><br>Mixed: Design meetings, semi-structured interviews. Thematic analysis. Computer programming.<br><br>Which art forms were used?<br><br>Dance
	**Was data literacy defined and, if so, how?**<br><br>The authors explain data literacy in the context of the danceON project:<br><br>**"…**Data literacy -Make transparent the body position data captured by computers and/or sensors while providing an accessible interface to empower learners to understand, use, and manipulate their own data for the purposes of making art" (Section 3.3.3)
	**Was data literacy measured and, if so, how?**<br><br>No, the focus is on the design and development of the danceOn system in the context of innovative methods in data literacy education with a view to measurement and other goals in the future.<br><br>"The method provides an understanding of the complexities of the educational innovation in practice. In essence, work with youth and instructors was essential to our formation of danceON, and we consider insights gained from deploying early-stage tools both as part of our design process and as a way to assess the design. As demonstrated in prior DBR research, this work feeds into the iterative development of danceON ensuring that it becomes increasingly aligned with learning theory, design, measurement, and practice over time [38]" (Section 5).
	**Was there an evaluation of the role of the arts in enhancing data literacy and, if so, how?**<br><br>"We draw from observation notes, learner project videos, code, and working documents to identify successes and limitations of the current danceON prototype and deployment with respect to our initial four design goals" (Section 7).
**Study Population**	What population groups are being studied in the literature?<br><br>Students described as "young women of color" (Section 1)

	What was the sample size?<br><br>Total sample size unclear. Some details provided.<br><br>Re the design:<br><br>"We determined the design requirements of danceON through 11 formal interviews and 20 design meetings with SFD participants" (Section 3.2).<br><br>Re: two-week summer camp:<br><br>"11 learners enrolled in the first cohort, though 2 dropped out before submitting their final project contributions. 10 learners enrolled and completed the second camp" (Section 5.1).
**Research Setting**	Was it a community-based setting?<br><br>Was it an educational setting?<br><br>Educational. Online, due to Covid 19 pandemic. A Stem From Dance (SFD) summer camp.<br><br>Was it a healthcare setting?
**Findings/results**	**How have the arts have been used to enhance data literacy?**<br><br>"Our ongoing design-based research investigation with STEM From Dance (SFD) led us to create a tool to engage young women of color in computing and provide novices ease of entry into explorations at the boundaries of data and dance" (Section 9).
Was the evaluation of the role of the arts positive, negative or mixed?	**Was the evaluation of the role of the arts positive, negative or mixed?**<br><br>Mixed. Positive in terms of the value of the learning experience but less so in terms of errors and bugs in the computer programme.<br><br>"danceON clearly supports personally and culturally relevant engagement in a range of forms" (section 7.2)."Within a curriculum built upon reflective ideation in which young women generated themes and ideas to use as the basis of their dance, danceON succeeded at developing strong connections between the computational thought process and the artistic depiction of their ideas" (Section 8.2).<br><br>"However, learners struggled to independently realize their complex ideas under time constraints (§5.3.2), and experienced difficulties understanding the textual-language and debugging error messages (§5.2)" (Section 8.2)."While danceON successfully represented and

	exposed body position data, it could better facilitate interaction with and guide learner understanding of missing and incorrect data" (Section 8.3)<br><br>"Still, accurate data is a lesser priority than scaffolding an authentic learner experience of data wrangling" (Section 8.3).<br><br>"Our work sets a path for future) developments to explore the connections between dance and data-driven systems and to push the bounds of how learners can engage in meaningful embodied learning experiences" (Section 9). *[End of Payne et al. 2021]*

Sanei et al. 2023

**Charting Elements**	**Associated Questions**
**Publication Details**	Sanei, H., Kahn, J.B., Yalcinkaya, R., Jiang, S., and Wang, C. (2023) 'Examining How Students Code with Socioscientific Data to Tell Stories About Climate Change', *Journal of Science Education and Technology* 33, pp. 161-177 (2024). https://doi.org/10.1007/s10956-023-10054-z
**Study ID**	Sanei et al. 2023
**Title**	Examining How Students Code with Socioscientific Data to Tell Stories About Climate Change
Author(s)	**Who are the authors of the publication?**<br><br>Sanei, H., Kahn, J.B., Yalcinkaya, R., Jiang, S., and Wang, C.
Year of Publication	**When was the paper/study published?**<br><br>2023
Origin/Country of origin	**Where was the study carried out?**<br><br>USA
Publication Type	**Is the publication a journal article, book or book chapter, review, opinion paper, grey literature, other?**

	Journal article
**General Overview of Study**	
Aims/purpose	**What were the aims or purpose of the study?**<br><br>“In this paper, we report from a pilot design-based research project for high school youth in a virtual (synchronous) summer program in which we guided students to engage in programming to build dynamic data visualizations and to investigate and develop accompanying data stories about climate change. We examine how students developed computational data literacies through our learning design, guided by the following research question: How do students navigate across coding and storytelling activities to create data stories about climate change?” (p. 162).<br><br>**What was the rationale for using the arts?”**<br><br>The term ‘art’ or ‘arts’ is not used. The authors argue that, despite an increasingly data filled world with a demand for science across multiple fields and disciplines, opportunities “to learn how to wrangle data, build dynamic data visualizations, and tell compelling stories about important socioscientific issues (SSIs) are rare for youth” (p. 161).<br><br>They further argue that, in science education, designs for learning about data focus on either programming and coding or analyzing data trends. They state their position as:<br>“Our contribution is designing a holistic learning experience that combines coding and **storytelling practices** to support **computational data literacies**” (p. 162).
Rationale for Using the Arts in Data Literacy Context Choices: Increase accessibility<br><br>*Increase accessibility Yes*<br><br>*Increase engagement Yes*<br><br>*Develop critical thinking skills Yes*<br><br>*Other: develop computational literacy*	
Methodology	**What methodological design was utilized for the study?**<br><br>The study is described as a [pilot] “design-based research project” (p. 162).

Key findings relating to the role of the arts in data literacy	
**Methods**	**What specific methods (e.g. qualitative, quantitative, mixed methods) were utilized?**<br><br>Mixed. (The terms quantitative or qualitative are not used).<br><br>"Our design-based research study (Cobb et al., 2003), *Data VIZion: Telling the world with data*, was a free summer program to introduce diverse high school youth to both coding and storytelling practices associated with data visualizations about climate change. In design-based research, each round of the implementation and research results will directly inform the design and improve the understanding and research of the next round (Cobb et al., 2003). This study is the first round of implementation but builds on our (the research team's) prior research in designing and studying learning environments to support interdisciplinary modeling and storytelling with open, large datasets (Jiang & Kahn, 2020; Kahn & Jiang, 2020)" (p. 163).<br>Data gathering methods included recordings of the Zoom sessions, breakout rooms and learning activities, surveys and individual interviews.<br><br>Analysis:<br>"To better understand how students navigated coding and storytelling activities, we primarily used the **interaction analysis methodology** (Jordan & Henderson, 1995) to examine students' multimodal interactions with the coding platform, instructors, and other participants. Interaction analysis is a collaborative methodology for analyzing video records of activities to understand how participation in those activities, with the available tools or objects in the environment, is organized. It involves a micro-analysis or deep analysis of the verbal and nonverbal interactions among participants in short snippet (typically 3–5 min) of video records" (p. 165).<br><br>**Which art forms were used?**<br><br>Storytelling [not referred to as an art form in the article]
	**Was data literacy defined and, if so, how?**<br><br>The focus is on computational data literacy.<br><br>"We introduce *computational data literacies* as the collective group of practices that are increasingly necessary for social and scientific inquiry in our increasingly datafied and technology-driven society (Ryan, 2016).

	**Was data literacy measured and, if so, how?**<br><br>Researchers used "a pre-survey to gauge their [the students] prior experiences with coding, data, and data storytelling and a post-survey to see if students' attitudes toward and understanding of coding and data storytelling changed. We also conducted individual exit interviews (Appendix) where students talked about their data stories, their overall experiences with the program, challenges they encountered, and aspects they enjoyed" (p. 164).
	**Was there an evaluation of the role of the arts in enhancing data literacy and, if so, how?**<br><br>Analysing the data stories was not the focus:<br><br>"While the content of students' data stories was not the focus of our current analysis, tracking their development supported our understanding of the relationship between coding and storytelling for students and how data technologies can support SSI inquiry" (p. 165).
**Study Population**	**What population groups are being studied in the literature?**<br><br>"[F]ive teens (2 male, 3 female, ages 14–17, identifying as White, Asian, Latinx, and African American) with little to no coding background, from different high schools across two states in the Southeastern USA, through our team's network" (p. 163).
	**What was the sample size?** 5
**Research Setting**	Was it a community-based setting?<br><br>Was it an educational setting? It was an online setting with an educational focus. A summer project.<br><br>Was it a healthcare setting?
**Findings/results**	**How have the arts have been used to enhance data literacy?**<br><br>The project was designed to support the teenage students to develop computational literacies drawing on personal stories. The analysis showed that "students drew on personal experiences across both cycles [coding and storytelling], particularly in conversations with others. Our analysis suggests that the SSI context

	contributed to students' conceptual movement, perhaps by affording greater personalization of the data" (p. 171).
Was the evaluation of the role of the arts positive, negative or mixed?	**Was the evaluation of the role of the arts positive, negative or mixed?**<br><br>Mixed, in general, with storytelling as a component.<br><br>"This study examines how students engaged in coding and storytelling about climate change in a computational data learning environment. While students encountered challenges in remixing, our design for learning with a programming and data visualization platform afforded participation<br>in coding, data visualization, storytelling, and scientific reasoning simultaneously and in personally meaningful ways" (p. 174).<br><br>*[End of Sanei et al. 2023]*

Spence et al. 2021

**Charting Elements**	**Associated Questions**
**Publication Details**	Spence, J., Schachter, E., Saleem, A., Jia, B. (2021). Increasing Data-Knowledge Through Artistic Representation. In: Stephanidis, C., Antona, M., Ntoa, S. (eds) HCI International 2021 - Posters. HCII 2021. *Communications in*<br>*Computer and Information Science*, vol. 1419. Springer, Cham. https://doi.org/10.1007/978-3-030-78635-9_78
**Study ID**	Spence et al. (2021).
**Title**	Increasing Data-Knowledge Through Artistic Representation
Author(s)	**Who are the authors of the publication?**<br><br>Spence, J., Schachter, E., Saleem, A., Jia, B.
Year of Publication	**When was the paper/study published?**<br><br>2021
Origin/Country of origin	**Where was the study carried out?**<br><br>USA

Publication Type	**Is the publication a journal article, book or book chapter, review, opinion paper, grey literature, other?**<br><br>Book chapter
**General Overview of Study**	
Aims/purpose	**What were the aims or purpose of the study?**<br><br>A "user experience case study was conducted to develop a method and tool to empower users to artfully tell stories with data" (abstract p. 609).<br><br>What was the rationale for using the arts?<br><br>The authors state the need for data literacy education in, and access to, data visualization/storytelling. They argue that art creates a pathway to making complex data more accessible, and relatable to personal experience.<br><br>"Data visualization requires application of familiar subjects, Math, Art, and Psychology (Aparicio and Costa 2015), but the barrier of entry remains high because it is not a part of early education. Art is a natural medium for increasing data knowledge and practicing data visualization because it is accessible. Currently the skills for creating data-driven storytelling reside with professionals, but data-knowledge skills can be more widely shared through the medium of art" (p. 609).
Rationale for Using the Arts in Data Literacy Context Choices: Increase accessibility<br><br>*Increase accessibility* ***Yes***<br><br>*Increase engagement* ***Yes***<br><br>*Develop critical thinking skills* ***Yes***<br><br>*Other*	
Methodology	**What methodological design was utilized for the study?**<br><br>Described as a "user experience case study" (p. 609) and refers to "Human-centered design methodologies" (p. 610).<br><br>"When increasing data-knowledge and practicing data visualization through art, an obvious tension exists between creating traditional and accurate data representations and creating artistic and abstract data representations. Through this study, **human-centered design methodologies** were applied to first identify target users, second to understand the prevalence of this

	tension for them, and third to design a tangible solution to relieve<br>it" (p. 609).
**Key findings relating to the role of the arts in data literacy**	
**Methods**	**What specific methods (e.g. qualitative, quantitative, mixed methods) were utilized?**<br><br>Mixed methods: qualitative and quantitative.<br><br>"There are six phases of the human-centered design process: Understand, Empathize, Define, Ideate, Prototype, and Test. These phases are not intended to be conducted strictly linearly, but instead cyclically" (p. 610).<br><br>All methodologies: "Empathy Interviews, Task Analysis, Ideation, Prototype Art, and Test Interactive Prototype" (p. 610).<br><br>"After completing initial prototyping, an online usability test tool (UsabilityHub, Collingwood,<br>Victoria) was utilized to conduct user testing. UsabilityHub allowed researchers<br>to conduct remote and unmoderated user tests to gain further insight into the prototype. Researchers set up a 32-question usability test that consisted of both **quantitative** and **qualitative** questions. The system usability scale (SUS)was used to quantify the usability of the prototype" (p. 617).<br><br>**Which art forms were used?**<br><br>Visual art
	**Was data literacy defined and, if so, how?**<br><br>Data literacy is not formally defined but the authors explain their perspective throughout, e.g.<br><br>"In this new connected world, it's vital to not only understand data concepts but also leverage data to skilfully communicate" (p. 609).
	**Was data literacy measured and, if so, how?**<br><br>The online tool was tested at the final stage of the research project. A survey was used to assess user interaction with the tool, a prototype developed for future use so not a measurement of data literacy.<br><br>A 7-point Likert scale was used to assess user interaction with the online tool (pp. 617-618).

	**Was there an evaluation of the role of the arts in enhancing data literacy and, if so, how?**<br><br>The project was evaluated by the researchers based on insights that were gleaned from each stage of the research process [outlined previously] which informed the development of the creative online data visualization tool. These were: analysis of interviews, a task analysis to define key moments in the art creation process, creation of pilot art pieces to assess how users would create art derived from raw data, and a user survey to assess the online prototype tool designed to enhance data literacy through visual art.
**Study Population**	**What population groups are being studied in the literature?**<br><br>Empathy interviews: “one was the founder of a data-literacy start-up, three were data scientists with professional data visualization experience,<br>one was an art teacher, one was a STEAM parent, and four were professional artists” (p. 611).
	**What was the sample size?**<br><br>“Remote empathy interviews were conducted with **ten** subject-matter experts to understand<br>key aspects and challenges for creating traditional data visualizations and for creating art” (p. 611). No number was stated for the user prototype user survey but appendix D shows **23** participants. The number of researchers is not specifically stated [researchers created art work].
**Research Setting**	Was it a community-based setting?<br><br>Was it an educational setting? Unclear. The focus is educational.<br><br>Was it a healthcare setting?
**Findings/results**	**How have the arts have been used to enhance data literacy?**<br><br>Art pieces were created as data representations to support the development of the online data visualization tool.
Was the evaluation of the role of the arts positive, negative or mixed?	**Was the evaluation of the role of the arts positive, negative or mixed?**<br><br>Mixed.<br><br>“By creating a tool for data visualization through artistic representation researchers were able to relieve the tension between accurate data representations and

	abstract data representations. The tool allowed users to create meaningful art pieces that can be translated to represent a the foundational data set or left as an artistic piece. The tool created is an effective way to increase data knowledge using humanized data visualization (p. 618).<br><br>"Despite the achievements in current study, a few limitations should also be acknowledged.<br>First, because this is a new space and product, researchers were able to define the minimum viable concept with very limited time and resources. Researchers were only able to test this new creative visualization tool concept as a low-fidelity interaction prototype, leveraging wizard-of-oz techniques to demonstrate to participants the art creation method. Second, users could not actually create their own piece of art from data of their choosing using the current prototype, instead, all pieces of artwork were created by researchers rather than real users. Ideally researchers would have been able to give the assignment to unbiased users meeting target user criteria to help inform the art creation method and user interface design. Third, researchers' initial intent was to develop a tangible solution for users between the ages of ten and eighteen, grades fifth through twelfth, to increase their data-knowledge and further promote STEAM education. However, due to special IRB concerns and pandemic circumstances, no young (end of 618) participants were recruited in this study" (p. 619).<br><br>*[End of Spence et al. 2021]*

Stornaiuolo 2020

**Charting Elements**	**Associated Questions**
**Publication Details**	Stornaiuolo, A. (2020) 'Authoring Data Stories in a Media Makerspace: Adolescents Developing Critical Data Literacies', *Journal of the Learning Sciences*, 29(1), pp. 81-103<br>https://doi.org/10.1080/10508406.2019.1689365
**Study ID**	
**Title**	'Authoring Data Stories in a Media Makerspace: Adolescents Developing Critical Data Literacies'
Author(s)	Who are the authors of the publication?<br><br>Stornaiuolo, A.

Year of Publication	**When was the paper/study published?**<br><br>2020
Origin/Country of origin	**Where was the study carried out?**<br><br>USA
Publication Type	**Is the publication a journal article, book or book chapter, review, opinion paper, grey literature, other?**<br><br>Journal article
**General Overview of Study**	
Aims/purpose	**What were the aims or purpose of the study?**<br><br>"This paper reports on a study of high school students' data literacy practices from the perspective of critical data literacies (e.g., Hautea, Dasgupta, & Hill, 2017; Tygel & Kirsch, 2015), a framework that emphasizes the importance of youth perspectives about data and foregrounds issues of race and power" (p. 82).<br><br>"The author/researcher and the media arts teacher collaborated to design an educational intervention that framed data as situated, ideological, and racialized, positioning youth as authors, architects, and interpreters of their own data stories" (p. 87). A "data unit" was created, building on the media arts students' particular interest in fashion design.<br><br>"One of the central goals of the unit was to encourage students to engage in critical analysis through their stories, particularly in illuminating data's role in reproducing educational injustice" (p.87).<br><br>A key argument is that students should have the opportunity to author their own data stories with a view to challenging dominant narratives about youth.<br><br>What was the rationale for using the arts?<br><br>The intervention was founded on the media students' interest in design, particularly fashion design. The "study was conducted in the media production makerspace (MPM)" (p. 87).<br><br>The author draws on data literacy literature to make a case for centralizing "youth perspectives in understanding the role of data in young people's digitally connected lives, highlighting especially the importance of expanding what "counts" as data to include personal narratives, art, and everyday activities" (p. 83).

Rationale for Using the Arts in Data Literacy Context Choices: Increase accessibility<br><br>*Increase engagement yes*<br><br>*Develop critical thinking skills yes*<br><br>*Other: express lived experience through artistic representations of the students' own stories –*	
Methodology	**What methodological design was utilized for the study?**<br><br>The study is described as a "social design research study".
**Key findings relating to the role of the arts in data literacy**	
**Methods**	**What specific methods (e.g. qualitative, quantitative, mixed methods) were utilized?**<br><br>Qualitative:<br><br>"One member of the research team participated in the MPM for at least one period each schoolday, acting as participant-observers and collecting observational, artifactual, and interview data. Observational data included 28 field notes, 20 audio recordings of class sessions (which were logged and tagged to field notes), and audio (end of p. 88) recordings of informal conversations with students and teachers" (p.89).<br><br>"Artifactual data included photos of students' work as well as all student projects. Pre- and post- survey data included information about how students conceived of data and its role in their lives. Also, 21 semi-structured interviews with students were conducted individually and in small groups at the end of the semester" (p. 89). "Data were analyzed iteratively using qualitative data analysis software" (p. 89).<br><br>**Which art forms were used?**<br><br>Visual arts: drawings, T-shirt design for exhibition.
	**Was data literacy defined and, if so, how?**<br><br>"While data literacy is often defined as the capacity of "individuals to access, interpret, critically assess, manage, handle and ethically use data" (Calzada Prado & Marzal, 2013, p. 126), such definitions foreground the individual skills, abilities, and technologies involved in working with data. More critical perspectives on data literacy are needed to challenge

	approaches that position data as objective and neutral measures of the social world rather than highlighting their situated, ideological, and racialized nature" (p.82).
	**Was data literacy measured and, if so, how?**<br><br>Data literacy is not measured. The project ran for one semester. Data literacy is analyzed utilizing the methods described, and findings are critically discussed by the author in terms of how the project addressed its original goals.
	**Was there an evaluation of the role of the arts I n enhancing data literacy and, if so, how?**<br><br>The arts are discussed throughout as tools to enhance understanding and communication of lived experience in the students' socio-cultural context.<br><br>"The personal nature of students' inquiries expanded what they thought "counted" as data to include seeing their lives as data: their narratives, art, interactions with others (p.91)".
**Study Population**	**What population groups are being studied in the literature?**<br><br>Adolescents in an urban public high school students. "CDS drew its population from neighborhoods across the city; in the 2017–2018 school year, the school's 252 students identified primarily as African American (75%) and Latinx (17%), as well as mixed race (3%), White (3%), Asian (1%), and Native American (1%).
	**What was the sample size?**<br><br>31
**Research Setting**	Was it a community-based setting?<br><br>Was it an educational setting? Yes<br><br>Was it a healthcare setting?
**Findings/results**	**How have the arts have been used to enhance data literacy?**<br><br>The students drew their personal data visualizations and subsequently designed T-shirts to represent and communicate the data stories they chose to tell. The art work represented data that the students had collected and analyzed.

	"Building on students' interests in fashion in other school spaces, particularly the design of personalized T-shirts and other clothing, the data unit foregrounded art and design activities that centered students' stories and esthetics as they designed T-shirts with their data art" (p.87).<br><br>The findings describe how the young people developed an understanding of their ability to use data in multiple ways and gained an understanding of "data as socially situated resources for meaning-making" (p. 81).
Was the evaluation of the role of the arts positive, negative or mixed?	**Was the evaluation of the role of the arts positive, negative or mixed?**<br><br>Mixed. Generally positive regarding the role of the arts in representing and communicating personal data stories but less so in terms of reaching the goal of enhancing critical data literacy and providing future opportunities to facilitate this.<br><br>"The study demonstrates the importance of youth seeing data as (end of p.97) personally relevant, as they brought their identities into their learning in meaningful ways. Certainly, students….came to see themselves as people who could use data for their own purposes, with data representing a powerful tool for telling their personal stories and data visualization an esthetically powerful communicative medium (p. 98).<br><br>The original aim of developing critical data literacy from a social, political and cultural perspective was "less visible in the study" (p.98).<br><br>The author argues that "the reality was that other goals, such as using data to understand oneself in new ways and engaging the esthetic dimensions of storytelling with data visualizations, became foregrounded in instruction and<br>students' subsequent inquiries" (p.98).<br><br>The author suggests that students need more opportunities and supportive teaching and learning infrastructure "to create critical and sustained engagement with culturally relevant data practices" (p. 98).<br><br>*[End of Stornaiuolo 2020]*

Sylvan 2018

**Charting Elements**	**Associated Questions**
**Publication Details**	Sylvan, E. (2018) 'Data literacy as storytelling practice in the open data/open minds project', *CEUR Workshop Proceedings 2018.* https://ceur-ws.org/Vol-2128/industrial7.pdf
**Study ID**	Sylvan (2018)
**Title**	'Data literacy as storytelling practice in the open data/open minds project', *CEUR Workshop Proceedings 2018.*
Author(s)	**Who are the authors of the publication?**<br><br>Sylvan, E.
Year of Publication	**When was the paper/study published?**<br><br>2018
Origin/Country of origin	**Where was the study carried out?**<br><br>USA
Publication Type	Is the publication a journal article, book or book chapter, review, opinion paper, grey literature, other?<br><br>Conference paper
**General Overview of Study**	
Aims/purpose	**What were the aims or purpose of the study?**<br><br>The paper describes a series of educational initiatives entitled "Open Data/Open Minds" aimed at engaging young students and their educators in "telling stories of their local concerns using science journaling, crafted visualization, and data investigation" (abstract). The cohort involved comprises STEM and English Language Arts educators.<br><br>What was the rationale for using the arts?<br><br>The author argues that storytelling is an important component in facilitating data literacy as it enables

	better understanding and communication of community data.<br>“A set of numbers, whether 100 or 1 billion does not describe a phenomenon or a relationship. It is when people build an understanding with data that they create meaning. It is the stories that we tell with data– nonfiction stories, stories based on facts, ones that lead us to<br>argumentation and understanding” (first page).<br>The author also notes the importance of using new ways to communicate knowledge and to have fun with data using, for example, “narrative argumentation<br>and the creation of crafted physical objects” (second page).
Rationale for Using the Arts in Data Literacy Context Choices: Increase accessibility<br><br>*Choices: Increase accessibility yes*<br><br>*Increase engagement Yes*<br><br>*Develop critical thinking skills Yes*<br><br>*Other*	
Methodology	**What methodological design was utilized for the study?**<br><br>The article outlines an educational approach.
**Key findings relating to the role of the arts in data literacy**	
**Methods**	**What specific methods (e.g. qualitative, quantitative, mixed methods) were utilized?**<br><br>Mixed methods. “This project combines<br>traditional scientific research techniques and innovative new technology with its use of paper and electronics” (first page).<br><br>**Which art forms were used?**<br><br>Visual arts, English Language Arts - narrative/storytelling.
	**Was data literacy defined and, if so, how?**<br><br>The author does not provide a specific definition of data literacy, but outlines the importance of combining STEM and arts tools as a pathway to understanding through “multimodal representations of data” (third page).<br><br>“…when we provide people with the tools to create representations of their understandings,

	we open up possibilities for them to participate in the public dialogue of ideas (first page).
	**Was data literacy measured and, if so, how?**<br><br>The paper refers to an organizational educational partnership *aimed* at enhancing data literacy by combining science, technology and the creation of physical artefacts and stories.
	**Was there an evaluation of the role of the arts in enhancing data literacy and, if so, how?**<br><br>In the context of the *Open Data/Open Minds* project, the author discusses how the arts (storytelling/narrative and physicality) render engagement with data fun and playful, thus making the data more accessible to students and their educators by enhancing meaning-making and understanding of data.
**Study Population**	**What population groups are being studied in the literature?**<br><br>Young students and their educators:<br><br>"While we focus on the 10-14 age group and their educators, the approach and materials support learners and learning spaces of all kinds" (first page)
	**What was the sample size?**<br><br>No sample size outlined.
**Research Setting**	Was it a community-based setting?<br><br>Was it an educational setting? Yes<br><br>Was it a healthcare setting?
**Findings/results**	How have the arts have been used to enhance data literacy?
Was the evaluation of the role of the arts positive, negative or mixed?	Was the evaluation of the role of the arts positive, negative or mixed?<br><br>Positive. "When people learn about science through the ODOM program, they leave knowing how to identify problems that are important to their communities, how to find and study existing scientific research, how to identify new issues within those problems, how to collect data related to those problems, how to analyze

	that data, and how to share it in a way that can impact civic engagement" (second page).<br><br>Through collaboratively working with and training educators: "We have all come to understand more deeply the ways in which inexpensive crafted experiences with technology can support youth to develop and [share] their voices and understand the power of data-driven argumentation" (third page) .<br><br>*[End of Sylvan 2018]*

Tylosky et al. 2021

**Charting Elements**	**Associated Questions**
**Publication Details**	Tylosky, N., Van Even, P., Claes, S., Pässilä, A., Knutas, A. and Wolff, A. 'Facilitating Data Inclusion and Empowerment through Arts-Based, Creative and Playful Approaches', *ACM International Conference Proceeding Series 2021*<br>Pages: 336-339.<br>https://doi.org/10.1145/3461564.3468164
**Study ID**	Tylosky et al. 2021
**Title**	'Facilitating Data Inclusion and Empowerment through Arts-Based, Creative and Playful Approaches', *ACM International Conference Proceeding Series 2021*
Author(s)	Who are the authors of the publication?<br><br>Tylosky, N., Van Even, P., Claes, S., Pässilä, A., Knutas, A. and Wolff, A.
Year of Publication	When was the paper/study published?<br><br>2021
Origin/Country of origin	Where was the study carried out?<br><br>Researchers are based in Finland and Belgium. The paper is about a potential workshop. The conference presentation was in the USA.
Publication Type	Is the publication a journal article, book or book chapter, review, opinion paper, grey literature, other?<br><br>Conference paper

**General Overview of Study**	
Aims/purpose	**What were the aims or purpose of the study?**<br>“The goal of this workshop is to explore how to improve data inclusivity and empowerment by minimizing the barriers that people may face in their encounters with data, whilst at the same time making data more fun to use” (p. 338).<br>**What was the rationale for using the arts?**<br>The authors argue that the general public experience barriers to data engagement and that arts-based methods enable people to communicate non-verbally and in ways that incorporate fun and creativity. “Combining arts-based approaches with data exploration and sensemaking – especially participatory sensemaking– has good potential for data inclusivity” (p.338). “Arts-based methods may also support empathy building through data” (p. 338).
Rationale for Using the Arts in Data Literacy Context Choices:<br>*Increase accessibility Yes*<br>*Increase engagement Yes*<br>*Develop critical thinking skills Yes*<br>*Other*	
Methodology	**What methodological design was utilized for the study?**<br>Qualitative. Description of proposed arts-based and data literacy workshop
**Key findings relating to the role of the arts in data literacy**	
**Methods**	**What specific methods (e.g. qualitative, quantitative, mixed methods) were utilized?**<br>Arts-based methods are described for the proposed potentially hybrid [online/in-person] workshop. The workshop also incorporates a research exchange at the start of the workshop [researchers share their research].<br>**Which art forms were used?**<br>“Participants will have the possibility to try out using a range of arts-based, creative and playful approaches for making sense of civic data sets, including roleplaying,

	improvisation, embodiment, sonification, physicalisation, narrativity, and so on" (p. 338).<br><br>"They could make sketches, short videos, clickable mockups or similar to convey their ideas. The organisers will suggest free and (where possible) online tools for these activities" (p. 338) [to communicate their ideas].
	**Was data literacy defined and, if so, how?**<br><br>Data literacy is not formally defined. However, throughout the paper, the authors note the importance of increasing community engagement with data and enhancing inclusivity through the arts and data.
	**Was data literacy measured and, if so, how?**<br><br>Data literacy was not measured as the paper describes a workshop design for future use.
	**Was there an evaluation of the role of the arts in enhancing data literacy and, if so, how?**<br><br>**Not applicable** as the paper discusses a workshop design for future use. "In this workshop, we will explore a number of arts-based, creative and playful approaches to support data interpretation of civic data sets. Participants will deploy these approaches to overcome data literacy issues and build empathy through data" (p. 339).
**Study Population**	**What population groups are being studied in the literature?**<br><br>Academics and professionals.<br><br>"Participants are welcome from a variety of backgrounds, we welcome artists, researchers in humanities and HCI, and practitioners" (p. 338).
	**What was the sample size?**<br><br>The authors stipulate that the workshop should have no more than 30 participants. "The maximum number of participants is 30, this would apply in both an online, virtual and hybrid situation" (p. 338).
**Research Setting**	Was it a community-based setting?<br><br>Was it an educational setting? Yes<br><br>Was it a healthcare setting?

**Findings/results**	**How have the arts have been used to enhance data literacy?**<br><br>N/A The discussion is about how the arts can help overcome barriers to data literacy and increase empathy through data.
Was the evaluation of the role of the arts positive, negative or mixed?	**Was the evaluation of the role of the arts positive, negative or mixed?**<br><br>N/A but positive in terms of potential.<br><br>*[End of Tylosky et al. 2021]*

Vacca et al. 2022a – I happen to be one of 47.8%

**Charting Elements**	**Associated Questions**
**Publication Details**	Vacca, R., DesPortes, K., Tes, M., Silander, M., Matuk, C., Amato, A. and Woods, P.J. (2022a) '"I happen to be one of 47.8%": Social-Emotional and Data Reasoning in Middle School Students' Comics about Friendship', *Conference on Human Factors in Computing Systems - Proceedings 2022.* https://doi.org/10.1145/3491102.3502086
**Study ID**	Vacca et al. 2022a
**Title**	'"I happen to be one of 47.8%": Social-Emotional and Data Reasoning in Middle School Students' Comics about Friendship'
Author(s)	**Who are the authors of the publication?**<br><br>Vacca, R., DesPortes, K., Tes, M., Silander, M., Matuk, C., Amato, A. and Woods, P.J.
Year of Publication	**When was the paper/study published?**<br><br>2022
Origin/Country of origin	**Where was the study carried out?**<br><br>USA
Publication Type	**Is the publication a journal article, book or book chapter, review, opinion paper, grey literature, other?**

	Conference paper
**General Overview of Study**	
Aims/purpose	**What were the aims or purpose of the study?**<br><br>“This study is part of a larger project called Data Literacy Through Art (DLTA), which brings together university researchers and cross subject middle school teachers to co-design curriculum and resources around art and data literacy” (p.2 np).<br>The authors “investigate the potential of narrative-based arts, such as comics, to help students situate their personal stories in a broader social context through data and engage students in informal inference making….[and] identify how their artifacts were indicative of their engagement in social-emotional learning” (pp. 2-3). [focussed on friendships]<br><br>**What was the rationale for using the arts?**<br><br>“Given their potential relevance to youth’s interests and their promise for making information more accessible (ex.[67], data comics offer an under explored opportunity for students to engage in reasoning about data and social-emotional issues” (p. 1).<br><br>“The unique features of comics have led researchers to examine the ways they can be connected to data reasoning and data-driven storytelling through capitalizing on the spatial layout and linear narrative to present information, charts, and data, that the reader can traverse through the comic panels [4]” (p. 3)<br><br>“Research has identified the potential for data comics to improve understanding and engagement with data over other visual presentations such as infographics [67] while providing access to complex information such as explaining how quantified-self apps handle data [57], or explaining results from HCI research studies [66]” (pp. 3-4).
Rationale for Using the Arts in Data Literacy Context Choices:<br><br>*Increase engagement Yes*<br><br>*Develop critical thinking skills Yes*<br><br>*Other*	
Methodology	**What methodological design was utilized for the study?**

	The paper outlines the research project and methods but does not specify a research design. It appears to be qualitative.
**Key findings relating to the role of the arts in data literacy**	
**Methods**	**What specific methods (e.g. qualitative, quantitative, mixed methods) were utilized?**<br><br>Mixed?? Analysis data comic artifacts and interviews<br><br>"They practiced data analysis skills, like graph reading and statistical reasoning, while creating digital comics to communicate stories about and with the data" (p. 2).<br>**Which art forms were used?**<br><br>Visual arts, data storytelling<br><br>Comics were developed using Pixton, an online data storytelling tool. Data comics with visual and textual components were used to convey data stories.
	**Was data literacy defined and, if so, how?**<br><br>Yes, they use D'Ignazio and Bhargava's (2016) definition:<br><br>"Data literacy can be understood as, "the ability to read, work with, analyse and argue with data as part of a larger inquiry process"" [17]. (first page)
	Was data literacy measured and, if so, how?<br><br>Qualitative analysis:<br><br>"Data Reasoning. To answer the question, what kinds of reasoning do students engage in through their creation of data comics, we pulled from three different data sources: (1) data comic artifacts, (2) group interviews with four students, and (3) individual interviews after the implementation with each of the art and the math teacher. We also triangulated this data through cross-referencing and contextualizing with our co-design meeting notes and artifacts that we draw on". (p. 7)
	**Was there an evaluation of the role of the arts in enhancing data literacy and, if so, how?**<br><br>The analysis throughout the paper outlines how the development of the data comics helped students to engage with data in various ways.<br><br>"Our analyses demonstrate that students were able to engage in various types of data reasoning within their comics" (p. 8)

**Study Population**	What population groups are being studied in the literature?<br><br>Students – with input from teachers.
	What was the sample size?<br><br>"In this paper, we present findings from the implementation of the data comic unit with 33 seventh graders, who examined data about themselves and their friendships". (p. 2)<br><br>"Through an analysis of 33 data comic artifacts, interviews with two middle school teachers, and interviews with four student participants" (p. 2)
**Research Setting**	Was it a community-based setting?<br><br>Was it an educational setting? Yes<br><br>Was it a healthcare setting?
**Findings/results**	**How have the arts have been used to enhance data literacy?**<br><br>"These findings contribute an understanding of how students make sense of data about personal, everyday experiences; and how an arts-integrated curriculum can be designed to support their mutual engagement in both data and social-emotional reasoning" (Abstract first page)
Was the evaluation of the role of the arts positive, negative or mixed?	**Was the evaluation of the role of the arts positive, negative or mixed?**<br><br>Positive:<br><br>"This study explores the role that comics can play in supporting data reasoning and social-emotional learning. Our results demonstrate that students created rich narratives that reflected a broad variety of contexts, characters and emotions. Moreover, comic-making engaged students in identifying what a data point means and connect it to context through describing and illustrating the data within their narratives" (p. 13)<br><br>"As a form of expressive construction [56], art-making can fuse a personally and culturally relevant discipline to data literacy, and invite learners to bring their own interests, experiences and skills to making meaning. Integrating such an art-based approach that centers data on relationships, can connect data reasoning to social-emotional learning in ways we hope will be further explored in future work" (p. 17).

	[End of Vacca et al. 2022a – I happen to be one of 47.8%]

Vacca et al. 2022b – What Do You Meme?

**Charting Elements**	**Associated Questions**
**Publication Details**	Vacca, R., DesPortes, K., Tes, M., Silander, M., Amato, A., Matuk, C., and Woods, P.J. (2022b) 'What Do You Meme? Students Communicating their Experiences, Intuitions, and Biases Surrounding Data Through Memes', *Proceedings of Interaction Design and Children, IDC 2022* pp. 212-224. https://doi.org/10.1145/3501712.3529739
**Study ID**	Vacca et al. 2022b
**Title**	
Author(s)	Who are the authors of the publication?<br><br>Vacca, R., DesPortes, K., Tes, M., Silander, M., Amato, A., Matuk, C., and Woods, P.J.
Year of Publication	When was the paper/study published? 2022
Origin/Country of origin	**Where was the study carried out?**<br><br>USA
Publication Type	**Is the publication a journal article, book or book chapter, review, opinion paper, grey literature, other?**<br><br>Conference paper
**General Overview of Study**	
Aims/purpose	**What were the aims or purpose of the study?**<br><br>"We explore the ways the students engaged in data reasoning and their argumentation practices as they communicate through their memes" (p. 212).<br><br>"We worked with two 7th grade art teachers at two different schools, to implement and explore a lesson in which students engaged in creative expression

	with DataMeme to communicate about graphs of existing<br>data" (p. 213).<br><br>[DataMeme is a tool]<br><br>**What was the rationale for using the arts?**<br><br>They note that "Typical approaches to promoting data literacy focus narrowly on mathematics and statistics concepts with little relation to students' own experiences" (p. 213). They argue that "Such approaches often fail to engage students in reasoning meaningfully about data and what they represent" (p. 213)<br>The data memes enabled the students to engage creatively with data, drawing on their own experiences. The authors refer to humanizing the data.
Rationale for Using the Arts in Data Literacy Context Choices:<br><br>*Increase accessibility*<br><br>*Increase engagement*<br><br>*Develop critical thinking skills*<br><br>*Other*	
Methodology	**What methodological design was utilized for the study?**<br><br>No specific mention of either qualitative or quantitative. The methods fit with qualitative, i.e. iterative inductive analysis.<br><br>"This work is part of a larger cross-curricular co-design project called Data Literacy Through the Arts (DLTA) [13]. Within the project researchers work collaboratively with middle school teachers to co-design curriculum and resources to support students in engaging in artistic data literacy practices and projects" (p. 213).
**Key findings relating to the role of the arts in data literacy**	
**Methods**	**What specific methods (e.g. qualitative, quantitative, mixed methods) were utilized?**<br><br>"The DataMeme tool is a web-based application designed as part of a larger cross-curricular co-design project called Data Literacy Through the Arts (DLTA)" (p. 214) [as above]<br><br>"The goal of the tool is to use narrative construction to engage in meaning making about data. Within the tool, users create data memes by selecting imagery and writing text" (p. 214).

	The findings emerged from data lessons with 7th graders. Their 56 memes were analysed.<br>Two research questions were used:<br>“RQ1. In what kinds of data reasoning do students engage<br>through their creation of data memes?<br>• RQ2. In what kinds of argumentation do students engage through their creation of data memes?” (p. 213).<br><br>“The 56 data memes created by the students were coded by two researchers through an iterative, inductive coding analysis. The analysis of the data memes included the memes themselves (i.e. GIF they used, text they overlaid, and the graph they used), and any accompanying written explanations from the student that sought to explain their arguments and reasoning process”. 215<br>The data was collaboratively coded, discussed, and iteratively re-coded by two researchers, then reviewed. “The final two groups of codes were around<br>Data Reasoning (see Table 2) and Argumentation (see Table 3)” (p. 216).<br><br>**Which art forms were used?**<br><br>Visual arts, storytelling (memes).
	**Was data literacy defined and, if so, how?**<br><br>Yes, they draw on Woolf et al. 2016 and D’Ignazio & Bhargava 2016<br><br>“Data literacy can be understood as “the ability to ask and answer real-world questions from large and small data sets through an inquiry process, with consideration of ethical use of data” [62]” (p. 213) Woolf ref.<br><br>“Data literacy goes beyond mathematical and statistical literacy, and encompasses an inquiry process in which learners explore the contextual components of data as they engage in reading, analyzing, making decisions around, communicating about, forming arguments with, and being critical of data [10, 62]” (p. 212).
	**Was data literacy measured and, if so, how?**<br><br>No measurement tool is mentioned. The researchers used inductive iterative analysis to draw out the results and show how the students argued and reasoned with data. All of this is placed in the context of literature in the field. They discuss their own case within this context.

	**Was there an evaluation of the role of the arts in enhancing data literacy and, if so, how?**<br><br>“Through the construction of data memes, students moved from aggregated data points to narratives that sought to express a point of view or argument. In line with efforts to move data literacy towards a more humanistic stance, the kinds of data reasoning students engaged in through their creation of data memes, are the kinds that “humanize the personal narratives behind the numbers” (p. 221).
**Study Population**	**What population groups are being studied in the literature?**<br><br>7th grade middle school students
	**What was the sample size?**<br><br>“Fifty-six middle school students and two teachers across two schools participated in the study. All participants were proficient in English, and the study was conducted in English. In one school, the student population was: 33% White, 32% Black, 16% Hispanic, and 16% Asian. 46% of the student population qualifies for free or reduced lunch, 18% of students have disabilities, and 4% are English Language Learners. The other school was a private Catholic middle school located in a large urban area with a predominantly Latinx and Black or African American population (85%)” (p. 214).
**Research Setting**	**Was it a community-based setting?**<br><br>Was it an educational setting? **Yes.** 7th graders in two middle-schools.<br><br>Was it a healthcare setting?
**Findings/results**	**How have the arts have been used to enhance data literacy?**<br><br>*The DataMeme tool helped the students to argue and reason with data that held meaning for them. They created meaning also.*
Was the evaluation of the role of the arts positive, negative or mixed?	**Was the evaluation of the role of the arts positive, negative or mixed?**<br><br>Positive<br><br>“Our results demonstrate that students created arguments that reflected different kinds of data reasoning<br>such as considering contexts, implications, and relevant experiences. Moreover, data meme construction leveraged the rhetorical affordances of memes to

	support the conveying of emotions and values alongside cultural stereotypes and personal biases" (p. 221).<br><br>"The construction of data memes enabled learners to explore their own narratives and funds of knowledge that the data represent and hypothesize about other narratives implicit in the data. Using informal inference, students used the memes as forms of theorizing to account for the data in relation to the context" (p. 222).<br><br>*[End of Vacca et al. 2022b – What Do You Meme?]*

Van Den Bosch et al. 2022

**Charting Elements**	**Associated Questions**
**Publication Details**	Van Den Bosch, C., Peeters, N. and Claes, S. (2022) 'More Weather Tomorrow. Engaging Families with Data through a Personalised Weather Forecast', *IMX 2022 - Proceedings of the 2022 ACM International Conference on Interactive Media Experiences 2022,* pp. 1-10. https://doi.org/10.1145/3505284.3529972
**Study ID**	Van Den Bosch et al. (2022)
**Title**	'More Weather Tomorrow. Engaging Families with Data through a Personalised Weather Forecast'
Author(s)	Who are the authors of the publication?<br><br>Van Den Bosch, C., Peeters, N. and Claes, S.
Year of Publication	**When was the paper/study published?**<br><br>2022
Origin/Country of origin	**Where was the study carried out?**<br><br>Belgium
Publication Type	**Is the publication a journal article, book or book chapter, review, opinion paper, grey literature, other?**<br><br>Conference paper
**General Overview of Study**	

Aims/purpose	**What were the aims or purpose of the study?**<br><br>“In this paper, we present the design of a video-based data storytelling application that prompts children and their families to explore and interpret historical weather data through a personalised weather forecast” (Abstract p. 1).<br><br>What was the rationale for using the arts?<br><br>“..audiences are not as data literate as media producers expect them to be, causing misconceptions that might lead to a decrease of trust [22] or the spread of misinformation [9].<br>Data-driven storytelling techniques offer a reliable and engaging approach to enhance data literacy skills [4, 12, 29], but media organisations are not widely adopting these approaches” (p.2).<br><br>“Another advantage of video is the presence of story characters that viewers can relate to, which makes them empathise with the shared thoughts, and triggers critical reflections on those experiences [21]. These feelings of empathy and consideration can be stimulated even more when you personalise stories based on the viewer’s situation. [8]” (p. 2).<br><br>“Personalised data videos thus offer an entry point to engage both media producer and viewer with data. Despite this, their design and particularly the relation to their context are still relatively under-explored” (p. 2).<br><br>“Storytelling emphasises the performance it entails, such as a social experience that involves retelling with slightly different interpretations [21]. However, few studies have explored how different settings, such as a screen at home or a physical installation at an exhibition; and related social aspects, such as interacting with children, family or friends, facilitate engagement with personalised data stories, and in particular, how this affects critical refection” (p. 2)
Rationale for Using the Arts in Data Literacy Context Choices:<br><br>*Increase accessibility*<br><br>*Increase engagement*<br><br>*Develop critical thinking skills: “enhance critical reflection”*<br><br>*Other*	
Methodology	**What methodological design was utilized for the study?**

	"Empirical study" mentioned in keywords but not elsewhere.
**Key findings relating to the role of the arts in data literacy**	
**Methods**	**What specific methods (e.g. qualitative, quantitative, mixed methods) were utilized?**<br>"In this paper, we present the design of a video-based data storytelling application that prompts children and their families to explore and interpret historical weather data through a personalised weather forecast. The application was displayed at a 2-month summer exhibition of a popular television channel. In a **controlled comparative study,** we investigated how the application triggered reflection, as well as emotional and narrative engagement of families at home and at the exhibition. **We combined this approach with an in-the-wild study,** in which we observed spontaneous interactions of visitors" (p. 1 – abstract).<br>"We conducted two studies, following a mixed-method approach:<br>1) a comparative study of the experience at the exhibition versus at home, and 2) an in-the-wild study at the exhibition" (p. 4).<br>"We adapted the application to be experienced online, via the web browser, and changed the story accordingly (i.e. no reference to the exhibition was made)" (p. 4).<br>**Pre-study evaluation** "the performance of the personalised weather forecast in a pilot study to identify potential issues with story flow and data interpretation. Semi-structured interviews…allowed us to refine the graphic design" (p. 4).<br>A Likert scale questionnaire was used for the application's exhibition engagement feedback and the online engagement feedback.<br>Which art forms were used?<br>"Personalised video storytelling"
	**Was data literacy defined and, if so, how?**<br>Data literacy was not defined. "Data literacy skills" were referred to throughout.

	**Was data literacy measured and, if so, how?**<br><br>"After consuming the data video story, participants completed a 7-point Likert questionnaire that included engagement measures commonly used in the domain of film and television to reflect on the role of storytelling on emotion, narrative engagement and contemplation [3]" (p. 4).<br>The questions in the survey intended to measure reflective thinking, emotional engagement, and narrative engagement involved in interacting with the application,
	**Was there an evaluation of the role of the arts in enhancing data literacy and, if so, how?**<br><br>Following quantitative and qualitative data analysis, the authors note "Our findings reveal how external and internal story design elements support data engagement" (p. 6).<br><br>"We therefore believe linking data to memories is a strong narrative device to trigger data refection" (p.8).<br><br>"….data should not only be considered as an end point, they are a medium to share personal or social experiences [26]" (p.8).<br><br>Often in media design, effects are topic-related, causing them to be difficult to generalize [28]. However, our study demonstrated how personal and social connections to data, such as important event dates, memories and affection for particular characters, can form powerful entry points for data engagement" (pp. 8-9).
**Study Population**	What population groups are being studied in the literature?
	**What was the sample size?**<br><br>"For the condition at the exhibition (CE), we recruited 19 adult participants at the entrance of the exhibition. The sample existed of an adult with at least one child younger than 10 years (N = 10), at least one child between 10 and 16 years old (N = 4), with children older than 16 (N = 4), or alone (N = 1).We asked them to test the full application after which they were given a tablet computer to fill in the post-survey" (p. 5)<br><br>In the wild study: [observing people's engagement with the exhibition and then interviewing them]

	"The actions and conversations of 30 groups and 8 individuals were described in detail. The groups were mainly parents and children (N = 15) and parents and grandchildren (N = 9). There were also groups of children (N = 3) and other compositions (N = 3). The researcher present approached 10 of those groups that had spontaneously engaged with the application, to report on what they had discovered, in order to collect their open and spontaneous responses" (p. 5).<br><br>Pilot: 8 participants (all younger than 23 y.o., with 4 participants younger than 14 y.o.) (p. 4)<br><br>Comparative study:<br><br>"We executed a comparative, between-subject design study with 19 participants per condition, i.e. at the exhibition (CE) or at home" (CH) (p. 4) [condition exhibition/condition home] *It's not clear if these were the same participants.*
**Research Setting**	Was it a community-based setting? Yes. A public exhibition and people's homes (online).<br><br>Was it an educational setting?<br><br>Was it a healthcare setting?
**Findings/results**	**How have the arts have been used to enhance data literacy?**<br><br>The researchers used personalized video storytelling techniques via an interactive weather forecast application to foster engagement with data.<br><br>"…we present the design of an interactive weather fore-cast that reveals historical data of temperature and precipitation by means of personalised video storytelling techniques" (p. 9).
Was the evaluation of the role of the arts positive, negative or mixed?	**Was the evaluation of the role of the arts positive, negative or mixed?**<br><br>Positive:<br><br>"…we present the design of an interactive weather fore-cast that reveals historical data of temperature and precipitation by means of personalised video storytelling techniques" (p. 9).<br><br>"we learned that family interaction supports social reflection on the data presented, and that environmental conditions affect data engagement. Furthermore, **our findings suggest that establishing a connection between data and memories is a compelling**

	**device to foster engagement with data**, and support retelling of data stories, and that a trusted, familiar presenter can help overcome reticence with more complex types of data representations" (p. 9 – conclusion).<br><br>*[End of Van Den Bosch et al. 2022]*

Wei 2024

**Charting Elements**	**Associated Questions**
**Publication Details**	Wei, Y., 2024. Data Visualization in Art Education Research and Practice. *Art Education*, *77*(2), pp.30-38.
**Study ID**	Wei 2024
**Title**	Data Visualization in Art Education Research and Practice
Author(s)	**Who are the authors of the publication?**<br><br>Wei, Y.
Year of Publication	**When was the paper/study published?**<br><br>2024
Origin/Country of origin	**Where was the study carried out?**<br><br>USA
Publication Type	**Is the publication a journal article, book or book chapter, review, opinion paper, grey literature, other?**<br><br>Journal article
**General Overview of Study**	
Aims/purpose	**What were the aims or purpose of the study?**<br><br>The stated purpose is to "examine data visualization examples in business, marketing, social studies, and art education to understand the

	similarities and differences between scientific and artistic perspectives" (p. 30).<br><br>**What was the rationale for using the arts?**<br><br>The author notes that there is great potential for art educators to include data visualization in K-12 art classrooms as a means to foster data literacy, emphasizing the communicative power of art.
Increase accessibility<br><br>*Increase engagement*<br><br>*Develop critical thinking skills*<br><br>*Other*	Increase engagement<br><br>Develop critical thinking skills
Methodology	**What methodological design was utilized for the study?**<br><br>The author reviews data visualization as a method in art education research and practice.
**Key findings relating to the role of the arts in data literacy**	
**Methods**	**What specific methods (e.g. qualitative, quantitative, mixed methods) were utilized?**<br><br>An examination of "examples of data visualization in business, marketing, social studies, and art education to understand the similarities and differences between scientific and artistic perspectives" (Wei 2024, p. 30).<br><br>**Which art forms were used?**<br><br>Visual art
	**Was data literacy defined and, if so, how?**<br><br>Data literacy is not defined.
	**Was data literacy measured and, if so, how?**<br><br>Data literacy is not measured.

	**Was there an evaluation of the role of the arts in enhancing data literacy and, if so, how?**<br><br>The author evaluates the role of the arts through an examination of examples of data visualization projects. This includes literature on the topic, software programmes, art works, and art projects with the author's students.
**Study Population**	**What population groups are being studied in the literature?**<br><br>The paper refers to art scholars and students in general
	**What was the sample size?** N/A
**Research Setting**	Was it a community-based setting?<br><br>**Was it an educational setting?** Yes<br><br>Was it a healthcare setting?
**Findings/results**	**How have the arts have been used to enhance data literacy?**<br><br>The author examines literature and software programmes in the field, highlighting the benefits and potential of integrating data visualization in, for example, "in K-12 art classrooms, fostering students' data literacy, innovative thinking, and visual narrative skills" (Wei 2024, p. 34).
	**Was the evaluation of the role of the arts positive, negative or mixed?**<br><br>Positive in terms of recommendations and implications for merging data visualizations and art education research and practice. The author concludes by suggesting that "[d]ata visualization holds tremendous potential in both art education research and practice" (Wei 2024, p. 37).<br><br>*End of Wei 2024*

Wolff et al. 2019

Charting Elements	Associated Questions
**Publication Details**	Wolff, A., Gooch, D., Cavero, J., Rashid, U., Kortuem, G. (2019). 'Removing Barriers for Citizen Participation to Urban Innovation'. In: de Lange, M., de Waal, M. (eds) *The Hackable City*. Springer: Singapore, pp.153-168 https://doi.org/10.1007/978-981-13-2694-3_8
**Study ID**	Wolff et al. 2019
**Title**	'Removing Barriers for Citizen Participation to Urban Innovation'.
Author(s)	**Who are the authors of the publication?**<br>Wolff, A., Gooch, D., Cavero, J., Rashid U., Kortuem, G.<br>[In M. de Lange and M. de Waal (eds.), *The Hackable City*]
Year of Publication	**When was the paper/study published?**<br>2019
Origin/Country of origin	**Where was the study carried out?**<br>UK [Milton Keynes]
Publication Type	**Is the publication a journal article, book or book chapter, review, opinion paper, grey literature, other?**<br>Book chapter
**General Overview of Study**	
Aims/purpose	**What were the aims or purpose of the study?**<br>The Urban Data School (UDS) is the third of three initiatives aimed at removing barriers for citizen participation to urban innovation within a project named MK Smart. [Milton Keynes]<br>"The third initiative is the Urban Data School (UDS) which is a school engagement programme, teaching data skills in schools using some real Milton Keynes data sets in the domain of energy" (p.154).<br>"The Urban Data School (UDS) is an initiative designed to improve data literacy amongst 8–18-year-old school students. The UDS aims to create a next generation of

	(end of 160) school leavers who are comfortable in asking and answering questions from data, who can critique data, use it as evidence to tell stories and who can recognise opportunities for using data to their own benefit or the benefit of their community" (p.161).<br><br>"The eventual aim is to integrate additional data to make the UDS a national, or possibly international, resource" (Fig. 1). (p.161)<br><br>"The goal of the UDS is just not to educate students but to engage the teachers themselves in learning more about working with and from these types of data sets" (p.164).<br><br>**What was the rationale for using the arts?**<br><br>[explore storytelling and new ways to visualise data e.g. energy tree]<br><br>"Students worked in groups and were tasked with thinking how a collective data set across a number of homes could be used as part of the app design.<br>These design sessions reveal that, without prompting any ideas, students find difficulties in creating novel data visualisations that are beyond their normal experience with graphs and charts. However, with support students can begin to imagine new ways to create visualisations. One example of energy visualisation is shown in Fig. 3" (p 163).<br>(fig. 3 shows visualising energy as a tree –hand drawn).
Rationale for Using the Arts in Data Literacy Context Choices:<br><br>*Increase accessibility*<br><br>*Increase engagement*<br><br>*Develop critical thinking skills*<br><br>*Other: Exploring new ways to visualise data seems to be the most appropriate rationale*	
Methodology	**What methodological design was utilized for the study?**<br><br>The chapter reports on a project. No specific methodological design is referred to.
**Key findings relating to the role of the arts in data literacy**	

**Methods**	**What specific methods (e.g. qualitative, quantitative, mixed methods) were utilized?**<br><br>Mixed<br><br>Which art forms were used?<br><br>Visual arts – hand drawings/visualisations
	**Was data literacy defined and, if so, how?**<br><br>Yes:<br><br>“Data literacy is typically defined as the ability to explore, interpret, analyse and contextualise data. It may include a wide and diverse range of skills such as ‘the ability to: formulate and answer questions using data as part of evidence-based thinking; use appropriate data, tools and representations to support this thinking; interpret information from data; develop and evaluate data-based inferences and explanations; and use data to solve real problems and communicate their solutions (Vahey et al. 2006). This implies that teaching and improving data literacy would require a cross-disciplinary Approach” (p. 160).
	**Was data literacy measured and, if so, how?**<br><br>No specific measurement or measurement tool discussed.
	**Was there an evaluation of the role of the arts in enhancing data literacy and, if so, how?**<br><br>The evaluation is more about the project in general. Innovativeness and creativity are mentioned as part of that but the arts are not specified as a separate entity in terms of evaluation.<br><br>[Following a list of barriers to participation in innovation] “We do not want to conclude with a statement of doom and gloom. Early classroom trials have demonstrated the effectiveness of the UDS approach in eliciting novel questions and developing data literate students. Similarly, the Our MK initiative has highlighted the innovativeness and creativity of the citizens of Milton<br>Keynes in developing ideas to address the sustainability challenges the city faces” (p.166).<br><br>“This chapter and the work reported highlight the importance of researching how to overcome barriers to citizen innovation to ensure that citizens are fully aware

	of their environment and the possibilities they have to shape the cities they live in" (p. 166).
**Study Population**	**What population groups are being studied in the literature?**<br><br>"Lesson plans based on these data sets have been trialled in four schools—one primary school (year 5–9/10 years) and three secondary schools (2 with year 9–13/14 years,1 with year 7–11/12 years)—in Milton Keynes" (p. 162).
	**What was the sample size?**<br><br>Not specified.
**Research Setting**	Was it a community-based setting?<br><br>Was it an educational setting? Yes. Primary and secondary schools.<br><br>Was it a healthcare setting?
**Findings/results**	**How have the arts have been used to enhance data literacy?**<br><br>"The majority of citizens are not data literate. We have proposed the Urban Data School as a solution for ensuring that the next generation are more data literate. However, it will be many years before they form the bedrock of a city's citizens and we must continue to explore mechanisms to educate older generations about how to use data effectively" (p.165).
Was the evaluation of the role of the arts positive, negative or mixed?	**Was the evaluation of the role of the arts positive, negative or mixed?**<br><br>The arts were not separately evaluated in terms of enhancing data literacy but creativity and innovation are discussed in positive terms.<br><br>"The majority of citizens are not data literate. We have proposed the Urban Data School as a solution for ensuring that the next generation are more data literate. However, it will be many years before they form the bedrock of a city's citizens and we must continue to explore mechanisms to educate older generations about how to use data effectively" (p.165).<br><br>[End of Wolff et al. 2019]

Wolff et al. 2021

**Charting Elements**	**Associated Questions**
**Publication Details**	Wolff, A., Knutas, A., Pässilä, A., Lautala, J., Kantola, L., and Vainio, T. (2021)<br>'Designing SciberPunks as Future Personas for More than Human Design', *Conference on Human Factors in Computing Systems - Proceedings 2021*<br>https://doi.org/10.1145/3411763.3443443
**Study ID**	Wolff et al. 2021
**Title**	'Designing SciberPunks as Future Personas for More than Human Design'
Author(s)	**Who are the authors of the publication?**<br><br>Wolff, A., Knutas, A., Pässilä, A., Lautala, J., Kantola, L., and Vainio, T.
Year of Publication	**When was the paper/study published?**<br><br>2021
Origin/Country of origin	**Where was the study carried out?**<br><br>Finland presumably – all authors located there. It doesn't specifically say. The project was run online.
Publication Type	**Is the publication a journal article, book or book chapter, review, opinion paper, grey literature, other?**<br><br>Conference paper
**General Overview of Study**	
Aims/purpose	**What were the aims or purpose of the study?**<br><br>"The aim was to examine how environmental data could act as a bridge between people and nature, to encourage empathy towards 'more-than-human' perspectives" (first page).<br><br>"This work explores one approach for building empathy [referring to: towards nature] through indirect interactions mediated by environmental data, to either supplement direct experiences or for use when these are not possible" (Section 2-second page).

	**What was the rationale for using the arts?**<br><br>"In SciberPunk, we aim to utilise both arts-based methods and storytelling and narrative techniques to structure empathic experiences around data in the process of defining characteristics of new **future personas"** (Section 2.2 second page).<br><br>"The activities utilised arts-based methods as we were interested in the **experiential aspects of engaging with data** and how we **might foster creative and sensory experiences** with it." (abstract – first page)<br><br>"Essentially, SciberPunk is a future persona that experiences data as a seventh sense. It does not yet exist, but the question it allows us to ask is, if such a SciberPunk did exist with all these senses, then how might this change our relationship to the world around us?" (Section 3 – second page).<br><br>"The project collaboratively produced a series of sketches, props, photographs and videos that brought this concept to life (for one example of data curation, see fg.1)".<br>(Section 3 – second page) *[note: Figure 1 shows images and props from the project, including digital art]*<br><br>*Increase accessibility<br><br>*Increase engagement<br><br>*Develop critical thinking skills<br><br>*Develop empathy
Rationale for Using the Arts in Data Literacy Context Choices:<br><br>*Increase accessibility*<br><br>*Increase engagement*<br><br>*Develop critical thinking skills*<br><br>*Other*	
Methodology	**What methodological design was utilized for the study?**<br><br>Case study
**Key findings relating to the role of the arts in data literacy**	

**Methods**	**What specific methods (e.g. qualitative, quantitative, mixed methods) were utilized?**<br><br>Qualitative. They mention a ‘multiple methods approach’ within the arts-based methods section. They explain this as “combining different art genres” (section 2.1 – second page).<br><br>A reported limitation of the study was the **inability to conduct quantitative analysis** of questionnaires due to running the project online (because of COVID 19 restrictions). This also led to a reduction in the number of participants.<br><br>Qualitative: arts-based activities and reflexivity on process, ethnographic interviews; inductive thematic analysis; deductive in terms of analyzing broader literature.<br><br>**Which art forms were used?**<br><br>Sketches, props, photographs and videos<br><br>Table 1: visual and literary art, creating characters and playing the role, creating comic strips, creating sketches, communicating characters through writing and poetry [top of fourth page]
	**Was data literacy defined and, if so, how?**<br><br>Data literacy is not defined but when mentioned they reference their previous work Wolff et al. 2019<br><br>Annika Wolf, Michel Wermelinger, and Marian Petre. 2019. Exploring design principles for data literacy activities to support children’s inquiries from complex data. *International Journal of Human-Computer Studies* 129 (2019), 41–54.
	**Was data literacy measured and, if so, how?**<br><br>No specific measurement was used. They state that they couldn’t use quantitative methods such as questionnaires as the project was online due to the COVID 19 pandemic.
	**Was there an evaluation of the role of the arts in enhancing data literacy and, if so, how?**<br><br>“We present here the fndings and main insights gained from SciberPunk, related to 1) assessing the impact of the online move 2) empathy building 3) engagement with persona building activities 4) SciberPunk persona outputs. In each case, we outline the evaluation strategy

	that was used and draw out the key lessons learned" (Section 5 – fourth page).<br><br>Section 5.2: Empathy: (fifth page)<br><br>"To understand and evaluate the process of using art-based methods for empathy-building, we planned to conduct attitude assessments before and after the activities" (). They couldn't do this the programme had to move online. Instead, to assess the arts-based element of the programme [empathy-building], they used deductive thematic analysis. " Instead, on this occasion, we aimed to understand empathy via the application of deductive thematic analysis [8] on the answers the participant wrote down in their worksheets during the S2 to S4 workshop activities. The thematic codes were informed by empathy constructs [34, 35], and basic emotions as defined by Plutchik [31] (anger, fear, sadness, disgust, surprise, anticipation, acceptance, joy). Additionally, we analyzed any relevant nature-related sentiments that occurred in the responses" (Section 5.2 – fifth page).
**Study Population**	**What population groups are being studied in the literature?**<br><br>Youth education students
	**What was the sample size?**<br><br>5
**Research Setting**	Was it a community-based setting?<br><br>Was it an educational setting? Educational *context* - project participation was online in people's homes<br><br>Was it a healthcare setting?
**Findings/results**	**How have the arts have been used to enhance data literacy? (**See below)Hel
Was the evaluation of the role of the arts positive, negative or mixed?	**Was the evaluation of the role of the arts positive, negative or mixed?**<br><br>Mixed:<br><br>**Negative** in terms of the challenge of evaluating the **full project** [not necessarily the arts-based aspects] as it took place online during COVID 19. They state that this limited their ability to apply quantitative methods i.e. using questionnaires. In addition, they state that they would

	have had more participants if in-person workshops could have taken place.<br><br>**Positive:**<br><br>"The importance of this case study to the CHI community is in demonstrating how conversations about nature and environmental concerns can be developed, **utilsing an arts-based approach combined with real data**. This has potential to be extended and formalised into a co-design method especially for urban design scenarios as a way to provide an environmental voice to the process through the eyes of SciberPunk future personas" (Section 8 – seventh page).<br><br>*End of Wolff et al. 2021*

Woods et al. 2024

**Charting Elements**	**Associated Questions**
**Publication Details**	Woods, P.J., Matuk, C., DesPortes, K., Vacca, R., Tes, M., Vasudevan, V. and Amato, A., 2024. Reclaiming the right to look: making the case for critical visual literacy and data science education. *Critical Studies in Education*, *65*(5), pp.441-459.
**Study ID**	Woods et al. 2024
**Title**	Reclaiming the right to look: making the case for critical visual literacy and data science education
Author(s)	**Who are the authors of the publication?**<br><br>Woods, P.J., Matuk, C., DesPortes, K., Vacca, R., Tes, M., Vasudevan, V. and Amato, A.
Year of Publication	**When was the paper/study published?**<br><br>2024
Origin/Country of origin	**Where was the study carried out?**<br><br>UK (first author) Study conducted in the USA

Publication Type	**Is the publication a journal article, book or book chapter, review, opinion paper, grey literature, other?**<br><br>Journal article
**General Overview of Study**	
Aims/purpose	**What were the aims or purpose of the study?**<br><br>To examine the role of 'visuality' and aesthetics within the implementation of co-designed arts-infused data science projects in four US middle schools.<br><br>**What was the rationale for using the arts?**<br><br>The researchers grounded their work in the lived experience of students and teachers engaged in data-art inquiry.<br><br>The prevalence of data within schools and students' lives "represents a visualizing process.." (Woods et al. 2024, p. 442).<br><br>"The creation of data-art helps students to find their voice, ask personally relevant questions about data, and see themselves within data sets and data collection processes" (Woods et al. 2024, p. 448).
Increase accessibility<br><br>*Increase engagement*<br><br>*Develop critical thinking skills*<br><br>*Other*	Increase engagement<br><br>Develop critical thinking skills
Methodology	**What methodological design was utilized for the study?**<br><br>Co-design methodology, "working alongside four US based middle school math and arts teachers with the aim of developing 'data-art inquiry curriculum units' or projects that simultaneously engaged students in data science and art making practices" (Woods et al. 2024, p. 448).

**Key findings relating to the role of the arts in data literacy**	
**Methods**	**What specific methods (e.g. qualitative, quantitative, mixed methods) were utilized?**<br><br>Qualitative.<br><br>Student and teacher interviews.<br><br>Post implementation interviews – “all four groups of teachers and students from three schools” (Woods et al. 2024, p. 450)<br><br>**Which art forms were used?**<br><br>Digital collage, data comics, photography, dance
	**Was data literacy defined and, if so, how?**<br><br>Yes. “Data literacy […] includes skills related to gathering, constructing meaning from, and telling stories with data” (Matuk et al. 2022; Stornaiuolo, 2020) (Woods et al. 2024, p. 443).
	**Was data literacy measured and, if so, how?**<br><br>Data literacy was not measured.
	**Was there an evaluation of the role of the arts in enhancing data literacy and, if so, how?**<br><br>The researchers analyzed interviews with teachers and students to explore the concepts of ‘visuality’ and aesthetics in schools in the context of data literacy.
**Study Population**	**What population groups are being studied in the literature?**<br><br>US middle school teachers and students
	What was the sample size?<br><br>Not reported.
**Research Setting**	Was it a community-based setting?<br><br>**Was it an educational setting? Yes**

	Was it a healthcare setting?
**Findings/results**	**How have the arts have been used to enhance data literacy?**<br><br>The authors note that their work aligns with the work of Gil-Glazer (2020) and others who argue that “non-traditional data visualizations like data-art, can help develop a critical stance towards the visuality of schools […] giving the students the ‘right to look’” (Woods et al. 2024, p. 443) i.e. exploring what may normally be hidden in the context of visuality.<br><br>“The right to look, to demand access to the real and the agency to construct visuality for themselves, is therefore central to critical visual literacy” (Woods et al. 2024, p. 445).<br><br>The authors argue that critical visual literacy supports critical data literacy because students “develop the ability to ’read against’ rather than ‘reading with’ visual texts (including data visualizations)” (Woods et al. 2024, p. 448).
	**Was the evaluation of the role of the arts positive, negative or mixed?**<br><br>Positive. The authors argue that “as soon as the students in this study began to work with data, they began to claim the right to look […] the students rejected data that did not speak to their lived experience” (Woods et al. 2024, p. 450).<br><br>They recommend that “Within data science education specifically, critical data literacy curricula need to extend beyond merely critiquing methods of data collection and analysis to consider other ways of knowing and being outside of data science itself that, in conversation with data, allow students to construct a more holistic understanding of the world around them” (p. 456).<br><br>“…our study reveals that arts-infused pedagogies provide one (not necessarily guaranteed) avenue for students to reclaim this right to look” (p.456).<br><br>*End of Woods et al. 2024*

Zhao et al. 2024

**Charting Elements**	**Associated Questions**
**Publication Details**	Zhao, Y., Bertling, J., Hodge, L. and Dyer, E., 2024. Bridging Data and Art: Investigating Data-Art Connections in a Data-Art Inquiry Program. *Journal of Science Education and Technology*, pp.1-19
**Study ID**	Zhao et al. 2024
**Title**	Bridging Data and Art: Investigating Data-Art Connections in a Data-Art Inquiry Program
Author(s)	**Who are the authors of the publication?**<br>Zhao, Y., Bertling, J., Hodge, L. and Dyer, E.
Year of Publication	**When was the paper/study published?**<br>2024
Origin/Country of origin	**Where was the study carried out?**<br>USA
Publication Type	**Is the publication a journal article, book or book chapter, review, opinion paper, grey literature, other?**<br>Journal article
**General Overview of Study**	
Aims/purpose	**What were the aims or purpose of the study?**<br>The aim of the reported MVP programme was to implement “a transdisciplinary science education afterschool program” (Zhao et al. 2024, 3rd page). The data-art inquiry programme was designed to teach students the basics of data science, enabling the visualization of their data using artistic techniques.<br>**What was the rationale for using the arts?**<br>The data-art inquiry programme was designed to address the need for proficiency in data literacy due to the vast amounts of daily information people

	have to contend with in their daily lives. The authors note a lack of emphasis on meaningful engagement with data in current K-12 data science education in the USA. They argue that merging data and art practices shows promise as a method in data science education. Data-art inquiry can "enhance students' data literacy by engaging them in the processes of question generation, data collection and analysis, and using artistic methods to visualize an communicate data" (Zhao et al. 2024, 3rd page).
Increase accessibility<br>*Increase engagement*<br>*Develop critical thinking skills*<br>*Other*	Increase engagement<br>Develop critical thinking skills
Methodology	**What methodological design was utilized for the study?**<br>Design based research<br>"Design based research builds broad instructional models based on existing theory to investigate how people think, know, act, and learn to advance theoretical development" (Zhao et al. 2024, third page, n.p.).
**Key findings relating to the role of the arts in data literacy**	
**Methods**	**What specific methods (e.g. qualitative, quantitative, mixed methods) were utilized?**<br>Mixed<br>Design of a "13 week data-art inquiry programme called MVP (Mathematizing, Visualizing, and Power)" (Zhao et al. 2024, 3rd page, n.p.)<br>Post-programme interviews.<br>Epistemic network analysis (ENA) was used to investigate data-art connections (combines quantitative and qualitative analysis).

	**Which art forms were used?**<br><br>Visual art-artistic data visualizations
	**Was data literacy defined and, if so, how?**<br><br>No formal definition of data literacy.
	**Was data literacy measured and, if so, how?**<br><br>Data literacy was not measured.
	**Was there an evaluation of the role of the arts in enhancing data literacy and, if so, how?**<br><br>The primary analysis method was epistemic network analysis (ENA), deemed to be "an appropriate tool for investigating the co-occurrence and interconnections between qualitative codes" and "combines qualitative and quantitative" in addition to showing how concepts are connected in visual representations, capturing temporal changes, "provides comparative analysis and contextual insights, and is suitable for inter-disciplinary analysis" (Zhao et al. 2024, 5th page) but can also be technically and interpretively challenging when dealing with large or complex data sets.
**Study Population**	What population groups are being studied in the literature?<br><br>Student data-artists [after-school programme]<br><br>Age range: 13-17 years
	**What was the sample size?**<br><br>MVP Programme: 27 data artists<br><br>Six participated in interviews: 4 female 2 male<br><br>Described as: 1 Asian; 5 white
**Research Setting**	**Was it a community-based setting? Yes** (3 sessions: community learning events in various city locations).

	**Was it an educational setting?** Yes (12 sessions)<br><br>Was it a healthcare setting?
**Findings/results**	**How have the arts have been used to enhance data literacy?**<br><br>The MVP after-school data-art program was designed with the aim of enhancing data literacy by combining art and science.
	**Was the evaluation of the role of the arts positive, negative or mixed?**<br><br>Largely positive.<br><br>The researchers acknowledge that the results are based on three paired interviews by the six interviewees.<br><br>They offer practical suggestions aimed at inspiring future design of similar programs and "presented a model for implementing a long-term, afterschool, data-art inquiry program that offers students extensive experience in data practices, community topic exploration, and art production" Zhao et al. 2024, 15th page).<br><br>They conclude that data-art inquiry programs effectively merge art and science and "can be a notable example of STEAM education" (Zhao et al. 2024, 17th page). In addition, such programs enable meaningful exploration of data, creative expression, and data science learning experiences.<br><br>*End of Zhao et al. 2024*
